# Enabling the formation of native mAb, Fab′ and Fc-conjugates using a bis-disulfide bridging reagent to achieve tunable payload-to-antibody ratios (PARs)†

**DOI:** 10.1039/d2sc06318b

**Published:** 2023-03-09

**Authors:** Fabien Thoreau, Léa N. C. Rochet, James R. Baker, Vijay Chudasama

**Affiliations:** a Department of Chemistry, University College London 20 Gordon Street London WC1H 0AJ UK v.chudasama@ucl.ac.uk j.r.baker@ucl.ac.uk fabien.thoreau@outlook.fr

## Abstract

Either as full IgGs or as fragments (Fabs, Fc, *etc.*), antibodies have received tremendous attention in the development of new therapeutics such as antibody–drug conjugates (ADCs). The production of ADCs involves the grafting of active payloads onto an antibody, which is generally enabled by the site-selective modification of native or engineered antibodies *via* chemical or enzymatic methods. Whatever method is employed, controlling the payload–antibody ratio (PAR) is a challenge in terms of multiple aspects including: (i) obtaining homogeneous protein conjugates; (ii) obtaining unusual PARs (PAR is rarely other than 2, 4 or 8); (iii) using a single method to access a range of different PARs; (iv) applicability to various antibody formats; and (v) flexibility for the production of heterofunctional antibody-conjugates (*e.g.* attachment of multiple types of payloads). In this article, we report a single pyridazinedione-based trifunctional dual bridging linker that enables, in a two-step procedure (*re-bridging*/*click*), the generation of either mAb-, Fab′-, or Fc-conjugates from native mAb, (Fab′)_2_ or Fc formats, respectively. Fc and (Fab′)_2_ formats were generated *via* enzymatic digestion of native mAbs. Whilst the same reduction and re-bridging protocols were applied to all three of the protein formats, the subsequent click reaction(s) employed to graft payload(s) drove the generation of a range of PARs, including heterofunctional PARs. As such, exploiting click reactivity and/or orthogonality afforded mAb-conjugates with PARs of 6, 4, 2 or 4 + 2, and Fab′- and Fc-conjugates with a PAR of 3, 2, 1 or 2 + 1 on-demand. We believe that the homogeneity, novelty and variety in accessible PARs, as well as the applicability to various antibody-conjugate formats enabled by our non-recombinant method could be a suitable tool for antibody–drug conjugates optimisation (optimal PAR value, optimal payloads combination) and boost the development of new antibody therapeutics (Fab′- and Fc-conjugates).

## Abstract 2

Using a bis-pyridazinedione-based disulfide rebridging agent, we enable the formation of various mAb, Fab′ and Fc conjugates with the ability to tune payload loading on each construct.

## Graphical abstract

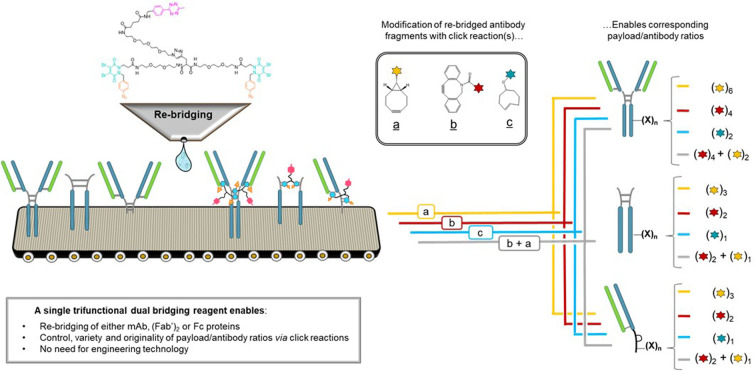

## 1 Introduction

Antibody–drug conjugates (ADCs) constitute a major class of therapeutics in the fight against cancer.^1^ Despite pioneering clinical evaluation in as early as 1983, they have only really emerged as a prominent therapeutic option in the early 2000s. There has been tremendous development in recent years with 11 ADCs being granted FDA approval in the last decade with 8 being approved since 2017.^2^ The success of ADCs lies in their capacity to combine, in a single biomolecule, the high selectivity of the antibody for its target with the strong cytotoxicity of small molecules attached to it. The field can even be extended to immunotoxins or immunocytokines, where a toxin or a cytokine is connected to the homing antibody, respectively.^2–4^ Antibody–drug conjugates are made of three main components – the antibody exerting selective binding for a cancer target, a highly cytotoxic payload, and a cleavable or non-cleavable linker connecting them.^1,5^ The number of cytotoxic payloads attached per antibody, known as the DAR (drug antibody ratio), is of crucial importance regarding pharmacokinetic and therapeutic activity of ADCs, as is their homogeneity. The first chemical methods to make ADCs relied on acylation of reactive lysine residues with activated esters of payloads. However, with dozens of lysine candidates at the antibody surface, this method led to highly heterogeneous ADC mixtures presenting a wide range of DAR and payload distribution when targeting average DARs of *ca.* 4.^6,7^ Modification of alternative, less abundant, nucleophilic amino acids such as tyrosine or reduced cysteine residues was also investigated, with the idea that less numerous reactive sites would improve homogeneity of modification.^8–10^ However, limited selectivity or reduced stability was observed. More recently, disulfide bridging reagents have been developed, relying on the reduction of accessible disulfide bridges (four in a IgG1 isoform) and their subsequent covalent reconnection *via* a small molecule.^11–14^ Homogenous ADCs with controlled DAR could be generated through these chemical procedures. These methods, as well as enzymatic modification of canonical amino acids or *N*-glycan region,^15^ can be directly applied to native antibodies. Recombinant technologies have also been developed to introduce additional cysteines, unnatural amino acids or tags for enzymatic reactions to enable selective antibody modification in subsequent steps.^16,17^ For a good overview on various site-selective modifications for production of homogenous ADCs, readers are directed to recent reviews.^8,18^

As mentioned, disulfide re-bridging is one of the most valuable approaches for the site-selective modification of native antibodies.^11,14,19–26^ Initially it was thought that the reaction of disulfide re-bridging reagents with the eight sulfhydryl reactive sites generated by the reduction of the four native interchain disulfides of an IgG1 typically offers a scope of DAR values that is limited to multiples of four. However, by introducing a chemical linker that has a single click handle and connects a pair of disulfide re-bridging reagents (*i.e.*, pyridazinediones (PDs)), to functionally re-bridge pairs of reduced disulfide bonds in an IgG, controlled access to a PAR (payload–antibody ratio) of 2 was enabled.^27^ Dannheim *et al.* also recently reported a disulfide re-bridging based method enabling access to PARs of 1, 2, 3 or 4, by employing linkers that connect four copies of divinylpyrimidine re-bridging agents (TetraDVP linkers), but have 1, 2, 3 or 4 terminal alkynes, respectively.^28^ Noteworthy in this case however is that the modulation of loading does not come from modification of the bridging agent itself, but from using different linkers connecting multiple bridging agents.

Despite numerous developments in the site-selective modification of antibodies to generate homogenous ADCs with controlled DARs, several key points still need to be addressed—(1) Antibodies being symmetrical biomolecules, either native inter-chain disulfides or engineered reactive functions are present in even numbers, and their modification generates DAR values almost restricted to one among 2, 4 or 8. Only a few exceptions enable alternative loading ratios such as 1, 3, or 5.^29–31^ Considering their pharmacokinetic and therapeutic influence, broadening the scope of accessible DAR values for a payload/antibody couple is of great interest; (2) The homogenous DAR enabled by optimised antibody modifications is typically limited to a single DAR. The few methods enabling a range of DARs all require incorporation of a different linker to access a different DAR, this constituting suboptimal modularity;^27,30,32^ (3) To improve anti-tumour efficacy, overcome tumour resistance, or enable theranostic approaches, growing evidence indicates that combining different types of payloads on an ADC can have a positive effect.^33,34^ However, apart from a few examples, methods reporting heterofunctionalisation of engineered,^31,33–35^ or native antibodies,^36–38^ are limited; (4) Antibody fragments are underexploited for therapeutic purpose. For instance, (Fab′)_2_ or Fab fragments conserve binding affinity of the parental antibody and benefit from a better tumour penetration due to their reduced molecular weight.^37,39–43^ Alternatively, Fc fragments can be conjugated to ligands to extend their half-life and/or immune capacity.^44,45^ Despite interesting attributes, the technologies for the selective modification of small antibody formats, except in rare examples,^46^ are not transferable from one format to another and do not enable the appendage of multiple and/or distinct payloads in a modular manner.

**Fig. 1:**
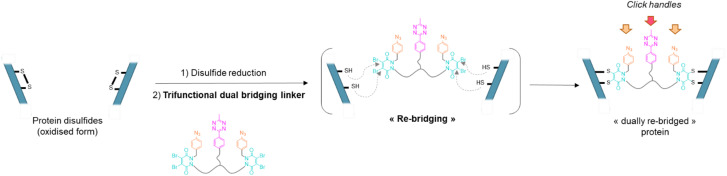
General principle of the dual re-bridging of two solvent accessible disulfides of a protein with a pyridazinedione-based trifunctional dual bridging linker, enabling introduction of corresponding click handles.

To address the above challenges, and building on previous work in our lab,^13,14,27^ we report a disulfide re-bridging method that exploits the use of a novel trifunctional dual disulfide re-bridging linker (see Fig. 1). Using this reagent in a two-step re-bridging/click procedure on various antibody fragments and exploiting click reaction orthogonality, this single disulfide re-bridging linker enables the generation of either antibody-, Fab′-, or Fc-conjugates, with on-demand access to a range of four different PARs for each species, being either mono- or bi-functional, and without engineering. A total of 15 new protein–payload conjugates have been synthesised to exemplify our method, demonstrating its high control and versatility regarding accessible PARs and compatible antibody formats, without requiring for recombinant technology.

## 2 Results and discussion

**Fig. 2:**
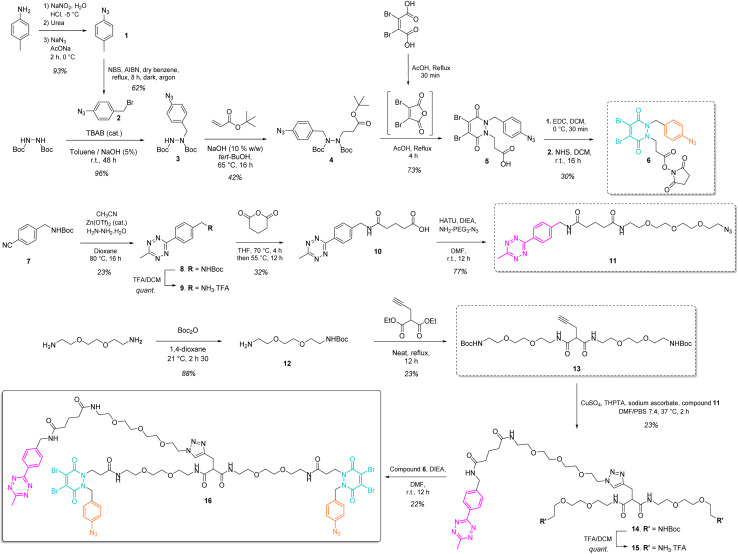
Convergent synthesis of trifunctional, dual bridging linker 16.

Our study began with the synthesis of a disulfide bridging linker that combines two phenyl azide-containing pyridazinedione bridging reagents (PD-PhN_3_) and a tetrazine-containing linker connecting them (compound 16, Fig. 2). The resulting trifunctional dual bridging linker is able to re-bridge two reduced disulfides to introduce two phenyl azide and one tetrazine moieties between them (Fig. 1). We envisioned that this *click handle*/*disulfide pair* ratio, coupled with exploitation of click reaction orthogonality, would allow us to apply this single bridging linker to various antibody formats (mAb, Fc, and (Fab′)_2_) to gain access to a range of controlled payload loadings for mAb-, Fc-, and Fab′-conjugates, including dual payload modification. It is noteworthy that the Tsuchikami group recently described the modification of an engineered antibody with a chemical platform bearing two alkyl azide and one methyl tetrazine handles enabling the grafting of MMAE and/or MMAF drugs to yield ADCs with various controlled DARs.^31^ Though, the described method was limited to a recombinant version of a mAb format, relied on enzymatic modification, and required the use of various linkers to yield different DARs.

## 3 Synthesis of the trifunctional dual bridging linker

The synthesis of the trifunctional bridging linker required prior synthesis of three building blocks – an activated ester of the clickable pyridazinedione (PD) bridging agent 6; the core linker 13 composed of a terminal alkyne-bearing malonate fragment flanked with two PEG chains each bearing a terminal amine function; and an azido-PEGylated methyl tetrazine fragment 11 (Fig. 2).

The NHS ester of PD 6 was synthesised based on previous reported procedures^13,27^ – a 1,2-diBoc protected hydrazine was first alkylated with azido benzyl bromide 2 in the presence of TBAB as a phase transfer catalyst in order to favour mono-alkylation and afford product 3. This was then reacted *via* a Michael addition with *tert*-butyl acrylate to yield protected hydrazine 4. Treatment of compound 4 with AcOH and dibromomaleic anhydride (formed *in situ* from dibromomaleic acid) enabled the one-pot deprotection and acylation of the hydrazine moiety to afford the PD 5. The corresponding NHS ester 6 was obtained *via* EDC mediated coupling with *N*-hydroxysuccinimide. This building block 6 constitutes a clickable, bridging reagent that can be grafted to an amine-bearing partner through the NHS ester reactivity.

The soluble and azide-bearing methyl tetrazine fragment 11 was synthesised based on described procedures.^47,48^ Boc protected cyanobenzyl 7 was engaged in reaction with hydrazine and CH_3_CN in the presence of a Zn(OTf)_2_ catalyst to afford Boc protected tetrazine 8, which was subsequently deprotected under acidic conditions to yield methyl tetrazine amine 9. Treatment with glutaric anhydride afforded methyl tetrazine acid 10, which was reacted in an amide coupling with NH_2_–PEG_3_–N_3_ to yield the methyl tetrazine azide 11. The core linker 13 was generated *via* reaction of two molecules of BocNH–PEG_2_–NH_2_12 with diethyl 2-(prop-2-yn-1-yl)malonate.

With building blocks 6, 11 and 13 in hand, we could realise the assembly of the final trifunctional dual bridging linker 16. A copper click reaction was employed to generate the Boc-protected methyl tetrazine-bearing core linker 14. Boc deprotection afforded methyl tetrazine-bearing core linker 15, which could be engaged in an amide coupling reaction with activated ester 6 to yield final compound 16 (Fig. 2).

## 4 Antibody & antibody fragments modification *via* trifunctional dual bridging linker and click reactions

Compound 16 is composed of two PD bridging agents (in blue) enabling the simultaneous re-bridging of two reduced cysteine bridges; two phenyl azide moieties (in orange) present on each of the PD motifs, allowing dual reactivity; three PEG motifs overall to improve solubility; and a central methyl tetrazine handle (in pink) to enable a third point of reactivity, possibly orthogonal to that of phenyl azide handles. Hence, compound 16 is a penta-reactive, trifunctional, dual bridging agent able to incorporate two phenyl azide (phenyl-N_3_) and one methyl tetrazine (MeTz) click handles between two cysteine bridges of a protein upon their reduction and re-bridging. It theoretically enables a ratio of two phenyl-N_3_ and one MeTz for each couple of accessible cysteine bridges insofar as they are close enough to be connected. We therefore evaluated linker 16 in the rebridging of various antibody formats (mAb, (Fab′)_2_ and Fc), using SDS-PAGE to approximate the relative amount of the products formed upon rebridging/conjugation and LC-MS for precise conjugate mass analysis.

### 4.1 Application to antibody–mAb-conjugates

**Fig. 3:**
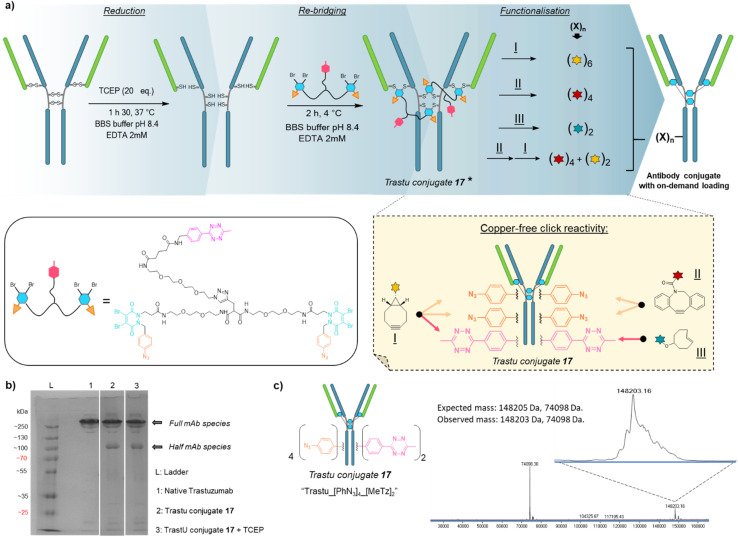
Application of trifunctional, dual bridging linker 16 to antibody format: (a) reduction, re-bridging and functionalisation of native trastuzumab antibody. Re-bridging with linker 16 allows introduction of four phenylazide and two tetrazine click handles, of which reactivity and orthogonality enables controlled access to various payload–antibody ratios (PARs) depending on the click handle present on reacting payload. * For clarity only the major full antibody species is drawn for Trastu conjugate 17 in part (a), but there is some minor half-antibody species present from intrachain rebridging in the Fc region, as seen in part (b) of the figure. (b) SDS-PAGE analysis of the re-bridging step. L: ladder, lane 1: native trastuzumab, lane 2: trastuzumab conjugate 17, generated *via* re-bridging of native trastuzumab with linker 16 (densitometry analysis with ImageJ software revealed a ratio full mAb/half mAb of *ca.* 80 : 20), lane 3: trastuzumab conjugate 17 under reducing condition (high excess TCEP) (densitometry analysis with ImageJ software revealed a ratio full mAb/half mAb of *ca.* 80 : 20). We also note that the antibody and antibody conjugates appear at higher masses than one would expect relative to the ladder, but that this is more-or-less consistent with the field. (c) Denaturing LC-MS analysis of trastuzumab conjugate 17. Expected mass: 148 205 Da, 74 098 Da (half-antibody); observed mass: 148 203 Da, 74 098 Da.

Based on previous work in our lab,^27^ we anticipated that the length and flexibility of dual bridging linker 16 would be convenient to connect any two cysteine bridges located in the Fab and/or the Fc hinge region of an IgG1 antibody. As a consequence, we hypothesised that a total of two copies of bridging linker 16 could be incorporated in an IgG1 antibody format possessing four inter-chain disulfides upon their reduction and re-bridging, resulting in incorporation of four phenyl-N_3_ and two MeTz handles into the antibody structure 17 (Trastu_[PhN_3_]_4__[MeTz]_2_). In a subsequent step, judicious selection of payloads bearing partner click handles could give a controlled access to a panel of homo- and bi-functional antibody–payload conjugates on-demand (Fig. 3a).

We selected trastuzumab antibody as an IgG1 model to evaluate our hypotheses. Re-bridging of this native antibody was realised in two steps, according reported procedure from our group^14^ – (1) reduction of four accessible disulfide bridges with excess TCEP, followed by removal of the remaining TCEP through ultrafiltration/buffer-exchange; (2) disulfide re-bridging with excess of dual bridging linker 16 before removal of unreacted linker *via* buffer exchange/ultrafiltration. Effective re-bridging was confirmed by SDS-PAGE analysis (Fig. 3b) since a main band was detected for re-bridged trastuzumab (lane 2) that indicated a molecular mass similar to that of unmodified trastuzumab (lane 1). A weaker band indicated the presence of a half antibody conjugate, resulting from an intrachain cross-linking of the cysteine residues in the hinge region during rebridging, as opposed to interchain cross-linking that covalently connects both heavy chains of the full mAb. We anticipate, by densitometry that relative amount of full antibody to half antibody is *ca.* 80 : 20. A very similar result/gel was obtained for the re-bridged trastuzumab conjugate despite using TCEP reducing conditions (lane 3), thus confirming that all four disulfides were re-bridged with TCEP-stable thiol-PD conjugates. Importantly, LC-MS analysis (Fig. 3c) confirmed that a trastuzumab with exactly two bridging linkers 16 incorporated was the main species generated, without need for further purification. Noteworthy, ionisation and denaturing LC conditions during analysis made half-antibody detection far more sensitive than full mAb. This is in accordance with a short study we carried out, which revealed that ∼50 kDa proteins can yield far higher mass spectrometry peak intensities than ∼150 kDa proteins, despite being the minor amount species (see ESI,† Section 4). However, SDS-PAGE demonstrated that full mAb is the main species (Fig. 3b). This result validated the hypothesis that reduced disulfides of trastuzumab can be connected with each other *via* a dual bridging linker 16, and that the four of them will be favourably re-bridged with only two linkers due to improved speed of intramolecular reactions. In addition, the dual bridging capacity of the linker is likely to reduce the occurrence of half-antibody resulting from disulfide scrambling during re-bridging, which is usually partially present with classical bridging agents.

**Fig. 4:**
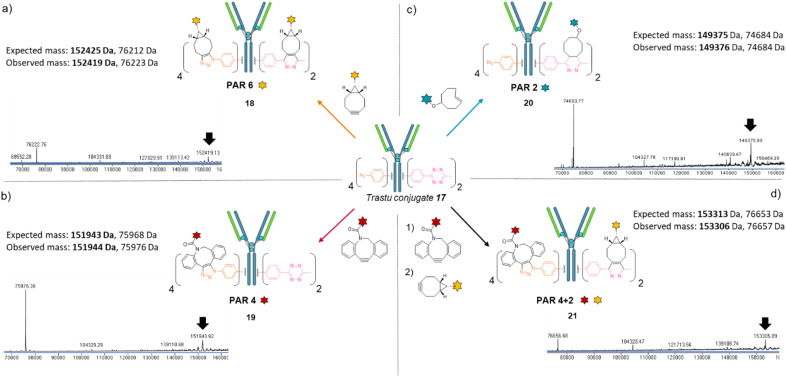
Denaturing LC-MS analysis after click reaction of Trastu_[PhN_3_]_4__[MeTz]_2_17 with (a) BCN-fluorescein to generate an antibody–payload conjugate with a PAR of 6 in fluorescein (Trastu_[fluorescein]_4__[fluorescein]_2_) *via* SPAAC and IEDDA reactions; (b) DBCO-rhodamine to generate an antibody–payload conjugate with a PAR of 4 in rhodamine (Trastu_[rhodamine]_4__[MeTz]_2_) *via* SPAAC reaction; (c) TCO–biotin to generate an antibody–payload conjugate with a PAR of 2 in biotin (Trastu_[PhN_3_]_4__[biotin]_2_) *via* IEDDA reaction; (d) DBCO-rhodamine followed by BCN-fluorescein to generate a heterofunctional antibody–payload conjugate with a PAR of 4 in rhodamine and 2 in fluorescein (PAR 4 + 2) (Trastu_[rhodamine]_4__[fluorescein]_2_) *via* sequential SPAAC and IEDDA reactions.

We next investigated the possibility that this single batch of re-bridged antibody conjugate 17 (Trastu_[PhN_3_]_4__[MeTz]_2_) could be modified to access, on-demand, various antibody-conjugates presenting homogeneous payload/antibody ratios (PARs) of 6, 4, or 2 and even a bi-functional PAR of 4 + 2. We anticipated that a BCN–payload would react with both MeTz (*via* inverse-electron demand Diels Alder) and phenyl-N_3_ (*via* strain-promoted cycloaddition) handles to result in the grafting of 6 payloads on the antibody (PAR 6). Hence, re-bridged trastuzumab conjugate 17 was incubated with an excess of model BCN-fluorescein for 3 h. After buffer exchange/ultrafiltration, the resulting conjugate was submitted to LC-MS analysis which confirmed selective formation of Trastu_[fluorescein]_4__[fluorescein]_2_ species 18 in good purity (Fig. 4a). This result demonstrated that the mAb modification procedure combining bridging linker 16 and a payload bearing a BCN click handle gives access to a homogeneous PAR of 6, which is rarely accessible with classical methods. Alternatively, trastuzumab conjugate 17 was incubated with a model DBCO-rhodamine, anticipating that electron-deficient DBCO would selectively react with phenyl-N_3_, leaving the MeTzs unreacted.^49^ LC-MS analysis of resulting conjugate confirmed the grafting of four DBCO-rhodamine on the antibody (Trastu_[rhodamine]_4__[MeTz]_2_19) with good purity and homogeneity (Fig. 4b), confirming that the mAb modification procedure combining bridging linker 16 and DBCO click handle gives access to a homogeneous PAR of 4. To further exploit phenyl-N_3_ and MeTz reactivity, trastuzumab conjugate 17 was incubated with excess of TCO-biotin or TCO-cyanine-5, expecting that TCO handle would react faster with MeTz handle to form a covalent link, while eliminating or leaving unreacted the phenyl-N_3_ groups, depending on the payload and reaction conditions.^50^ To our delight, both biotin (compound 20) and cyanine-5 (compound S20, see ESI†) could be grafted on the antibody as confirmed by LC-MS (Fig. 4c and ESI†). In both cases, exactly two copies of the payloads were attached to the antibody, accompanied with a loss of 0 to 4 methyl-phenyl azide fragments *via* TCO-triggered 1,6-elimination.^50^ Noteworthy, TCO-cyanine-5 demonstrated an improved tendency to induce methyl-phenyl-azide elimination over time when compared to TCO–biotin, and a reduced reaction time (30 min) allowed generation of a mAb–biotin conjugate with almost no trace of elimination (Fig. 4c). This result demonstrated that the antibody modification procedure combining bridging linker 16 and a payload bearing a TCO click handle gives access to a corresponding PAR of 2, while elimination mechanism can be either prevented or favoured through reaction condition tuning, to access homogeneous mixtures. Ultimately, we evaluated the possibility to exploit orthogonality to generate homogeneous bi-functional antibody-conjugates. To this end, we sequentially treated trastuzumab conjugate 17 with DBCO-rhodamine, followed by BCN-fluorescein. LC-MS analysis confirmed the formation of a homogeneous antibody-conjugate with a mass corresponding to exactly four DBCO-rhodamine and two BCN-fluorescein attached to trastuzumab 17*via* DBCO/phenyl-N_3_ and BCN/MeTz click reactions respectively, to generate Trastu_[rhodamine]_4__[fluorescein]_2_21 (Fig. 4d).

Altogether, we demonstrated that a single batch of IgG1 antibody re-bridged with two copies of linker 16, combined with a payload bearing either a BCN, a DBCO or a TCO click handle enabled the generation of corresponding antibody–payload conjugates with a PAR of either 6, 4 or 2, respectively, while a combination of two payloads each bearing a DBCO or a BCN click handles enabled a bi-functional PAR of 4 + 2. Such originality and modularity in accessing both mono- and bi-functional PARs, from a single type of bridging linker and a single batch of antibody, without requiring recombinant technology is, to the best of our knowledge, unprecedented. We anticipate the trifunctional dual bridging linker 16 and described method could be of great interest for antibody-conjugates optimisation, including dual drug and theranostic ADCs.

### 4.2 Application to (Fab′)_2_ format – Fab′-conjugates

Prompted by the successful application of our method to a full antibody (IgG1), we envisioned to extend its use to other antibody formats. Indeed, antibody-conjugates are very promising, but can suffer from low tumour penetration due to high molecular weight. For this reason, alternative formats such as Fab-conjugates and F(ab′)_2_-conjugates are also investigated. While keeping their binding ability *via* one or two Fab moieties, the lack of Fc fragment or both the Fc and a Fab fragments results in lower circulation time but potentially better tumour penetration.

**Fig. 5:**
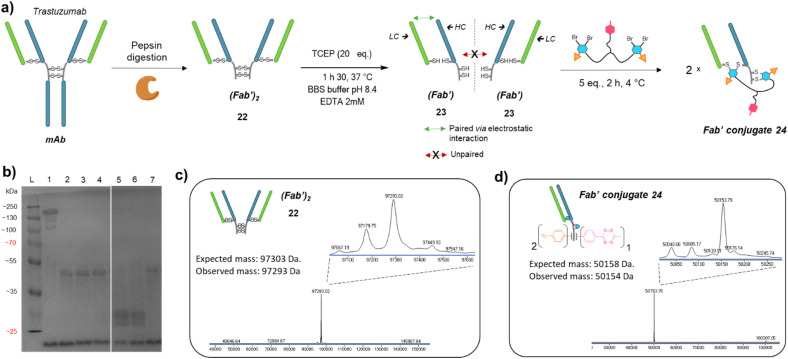
(a) Reaction scheme of enzymatic (Fab′)_2_ production from native mAb, followed by reduction and re-bridging steps. Low electrostatic HC–HC interactions after (Fab′)_2_ reduction with TCEP prevents the two Fab′ fragment to be maintained together, promoting intra-molecular re-bridging of single Fab′ formats. (b) SDS-PAGE analysis comparing unmodified (Fab′)_2_ and re-bridged (Fab′)_2_ under non-reducing or reducing conditions (excess TCEP). L: ladder; lane 1: (Fab′)_2_; lane 2: (Fab′)_2_ reduced and re-bridged with linker 16 at 4 °C; lane 3: reduced and re-bridged with linker 16 at 37 °C; lane 4: reduced and re-bridged with linker 16 at 60 °C; lane 5: (Fab′)_2_ crude after TCEP reduction step, yields HC and LC; lane 6: native (Fab′)_2_ analysed under reducing conditions (TCEP), yields HC and LC; lane 7: (Fab′)_2_ re-bridged with linker 16 at 4 °C, analysed under reducing conditions (excess TCEP). Densitometry analysis confirmed that no (Fab′)_2_ species remained on lanes 2 to 7, and neither HC nor LC were detected on lanes 2, 3, 4 and 7. (c) Denaturing LC-MS analysis of (Fab′)_2_ format produced *via* native mAb pepsin digestion. Even though not detrimental, two extra peaks are observed (97 179 Da and 97 067 Da) that correspond to loss of an extra Leucine on one or both HCs.^53^ (d) Denaturing LC-MS analysis of Fab′ format re-bridged with bridging linker 16. HC: heavy chain. LC: might chain.

Given that trifunctional dual bridging linker 16 successfully re-bridged the four disulfides of a trastuzumab antibody, we decided to first investigate the (Fab′)_2_ format, which also encompasses four disulfides. To this purpose we primarily produced a trastuzumab (Fab′)_2_ antibody fragment 22*via* pepsin digestion of native trastuzumab following described procedures (Fig. 5a and c).^51,52^ (Fab′)_2_ reduction with TCEP, buffer exchange/ultrafiltration, and re-bridging with trifunctional dual bridging linker 16 was realised in the same way as for trastuzumab mAb (Fig. 5a). However, SDS-PAGE analysis revealed that no (Fab′)_2_ format was recovered (no band of ∼100 kDa) after the re-bridging step (Fig. 5b). Instead, a protein with a mass of ∼50 kDa was obtained, that we hypothesised to be a re-bridged Fab′ fragment (half (Fab′)_2_). Indeed, reducing SDS-PAGE analysis of (Fab′)_2_ after the re-bridging step (Fig. 5b, lane 7), did not yield the HC (∼25 kDa) and LC (∼23 kDa) bands that were observed for the native (Fab′)_2_22 (Fig. 5b, lane 6), demonstrating that re-bridging was able to maintain the LC–HC pairing (effective intra-Fab′ re-bridging), but not effective to maintain HC–HC pairing of native (Fab′)_2_ (no inter-Fab′ re-bridging). We hypothesised that HC–HC pairing was actually lost as soon as the (Fab′)_2_ got reduced, as a consequence of limited electrostatic interactions resulting from the truncature of the Fc fragment from the parental mAb. These limited HC–HC interactions would in turn prevent the two Fab′ fragments from being in close vicinity upon reduction, while LC and HC of each Fab′ would still be maintained together through high electrostatic interactions (Fig. 5a). As a consequence, the re-bridging step would actually be realised on separate Fab′ fragments rather than on (Fab′)_2_ fragment, and yield to incorporation of a single bridging linker 16 in each Fab′. This hypothesis was validated by LC-MS analysis that confirmed that reduction and re-bridging of (Fab′)_2_ fragments generated proteins with a mass corresponding to conjugate 24, a Fab′ fragment re-bridged with a single bridging linker 16, with excellent purity (Fig. 5b and d). Hence, incorporation of a single bridging linker 16 in the Fab′ scaffold allowed controlled introduction of two phenyl-N_3_ and one MeTz handles per protein fragment.

**Fig. 6:**
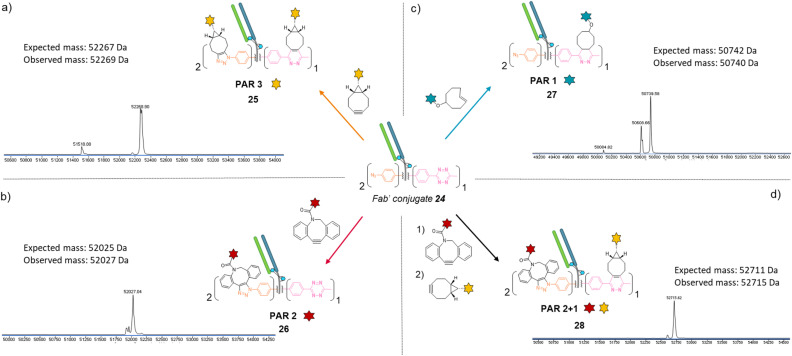
Denaturing LC-MS analysis after click reaction of re-bridged Fab′_[PhN_3_]_2__[MeTz]_1_24 with (a) BCN-fluorescein to generate a Fab′-payload conjugate with a PAR of 3 in fluorescein (Fab′_[fluorescein]_2__[fluorescein]_1_) *via* SPAAC and IEDDA reactions; (b) DBCO-rhodamine to generate a Fab′-payload conjugate with a PAR of 2 in rhodamine (Fab′_[rhodamine]_2__[MeTz]_1_) *via* SPAAC reaction; (c) TCO–biotin to generate a Fab′–payload conjugate with a PAR of 1 in biotin (Fab′_[PhN_3_]_2__[biotin]_1_) *via* IEDDA reaction; (d) DBCO-rhodamine followed by BCN-fluorescein to generate a hetero-functional Fab′-payload conjugate with a PAR of 2 in rhodamine and 1 in fluorescein (PAR 2 + 1) (Fab′_[rhodamine]_2__[fluorescein]_1_) *via* sequential SPAAC and IEDDA reactions.

Similar to previous antibody modification, we submitted the re-bridged Fab′ protein conjugate 24 (Fab′_[PhN_3_]_2__[MeTz]_1_) to various click reactions in order to access a range of homogenous PARs for Fab′–payload conjugates, in a controlled fashion. The click reaction between re-bridged Fab′ conjugate 24 and a BCN-fluorescein modified the three handles (MeTz and both phenyl-N_3_), resulting in a controlled payload/Fab′ ratio of 3 (Fig. 6a, Fab′_[fluorescein]_2__[fluorescein]_1_25). The click reaction with a DBCO-rhodamine selectively modified the two phenyl-N_3_ handles to generate a Fab′-rhodamine conjugate 26 with a PAR of 2 (Fig. 6b, Fab′_[rhodamine]_2__[MeTz]_1_), while the click reaction with a TCO–biotin selectively modified the MeTz handle to incorporate a single biotin per Fab′ (PAR 1), potentially accompanied with partial phenyl-azide elimination (Fig. 6c, Fab′_[PhN_3_]_1-2__[biotin]_1_27). Click reaction with TCO-Cy5 also led to a PAR 1, but with complete elimination of two phenyl-azide moieties (compound S27, see ESI†). The sequential addition of DBCO-rhodamine and BCN-fluorescein enabled selective modification of the phenyl-N_3_ handles and the MeTz handle, respectively, generating a bi-functional Fab′-conjugate presenting two rhodamine and one fluorescein fluorophores per Fab′, for a homogeneous PAR of 2 + 1 (Fig. 6d, Fab′_[rhodamine]_2__[fluorescein]_1_28).

Regarding their size and biological activity, Fab and Fab′ fragments are very similar in mass so methods employed to make corresponding protein-conjugates can be compared. To the best of our knowledge, in the context of Fab or Fab′ fragments, no other modification method enables a controlled access to a payload/antibody ratio of 3 without engineering; nor access to three different payload/antibody ratios from a single site-selective modification; and only few methods give access to bi-functional Fab or Fab′ formats. Given that our method combines all these features, we believe its modularity will be of great interest for optimisation and development of small antibody conjugates that rely on the exploitation of Fab binding sites.

### 4.3 Application to Fc format – Fc-conjugates

**Fig. 7:**
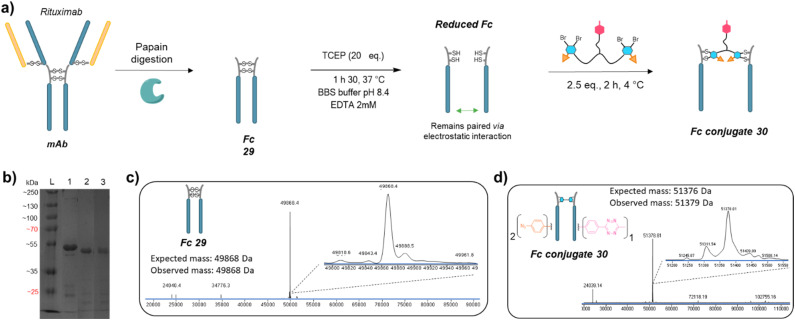
(a) Reaction scheme of enzymatic Fc production from native mAb, followed by reduction and re-bridging steps. (b) SDS-PAGE analysis comparing unmodified Fc (lane 1) and re-bridged Fc under non-reducing (lane 2) or reducing conditions (excess TCEP, lane 3). L: ladder. Densitometry analysis with ImageJ indicated that the band of Fc conjugate 30 accounted for 89% and 85% of total measured intensity on lanes 2 and 3, respectively. (c) Denaturing LC-MS analysis of Fc format produced *via* native mAb papain digestion. (d) Denaturing LC-MS analysis of Fc format re-bridged with bridging linker 16.

We next applied the bridging linker 16 to the re-bridging and functionalisation of an Fc fragment, which contains two disulfides in the hinge region. We expected that a single bridging linker 16 would be grafted per Fc fragment, *via* simultaneous re-bridging of two disulfide pairs (Fig. 7a). Papain digestion of rituximab antibody using described procedures afforded Fab and Fc fragments that could be separated *via* a protein A affinity column (Trastuzumab could not be used to isolate the Fc fragment since its Fab also has affinity for protein A, disabling the separation step).^37^ Fc fragment 29 was then submitted to the aforementioned reduction, buffer exchange/ultrafiltration, and re-bridging with trifunctional dual bridging linker 16. Both native and reducing SDS-PAGE indicated an effective re-bridging of the Fc fragment, while LC-MS analysis confirmed that only one bridging linker 16 was incorporated in the Fc fragment, with a very high purity and homogeneity. Fc fragment conjugate 30 thus encompassed two phenyl-N_3_ and one Me-Tz handles after re-bridging (Fc_[PhN_3_]_2__[MeTz]_1_). Conversely to the previous (Fab′)_2_ format, the electrostatic interactions between the two HCs of the (non-truncated) Fc enabled conservation of their pairing despite the disulfide reduction. The subsequent re-bridging thus reconnected the two HCs and no single HC was observed.

**Fig. 8:**
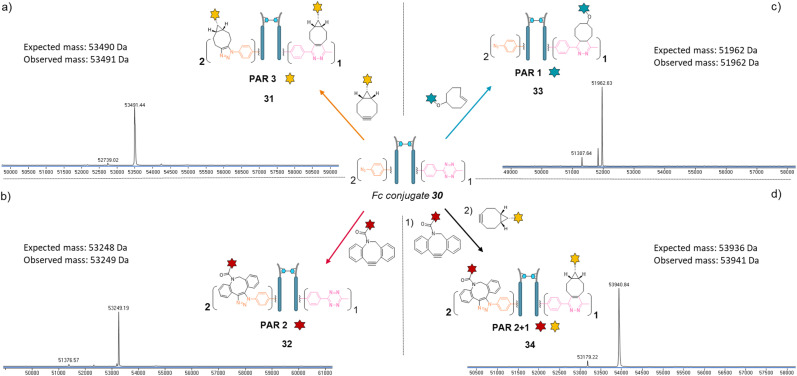
Denaturing LC-MS analysis after click reaction of re-bridged Fc_[PhN_3_]_2__[MeTz]_1_30 with (a) BCN-fluorescein to generate a Fc–payload conjugate with a PAR of 3 in fluorescein (Fc_[fluorescein]_2__[fluorescein]_1_) *via* SPAAC and IEDDA reactions; (b) DBCO-rhodamine to generate a Fc–payload conjugate with a PAR of 2 in rhodamine (Fc_[rhodamine]_2__[MeTz]_1_) *via* SPAAC reaction; (c) TCO–biotin to generate a Fc-payload conjugate with a PAR of 1 in biotin (Fc-[PhN_3_]_2__[biotin]_1_) *via* IEDDA reaction; (d) DBCO-rhodamine followed by BCN-fluorescein to generate a hetero-functional Fc–payload conjugate with a PAR of 2 in rhodamine and 1 in fluorescein (PAR 2 + 1) (Fc_[rhodamine]_2__[fluorescein]_1_) 34*via* sequential SPAAC and IEDDA reactions.

Copper-free click reactions with model payloads to generate a range of PARs on demand was then appraised. As expected, a BCN-fluorescein reacted on the two phenyl-N_3_ and the MeTz moieties through SPAAC and IEDDA, respectively, to yield an Fc-conjugate with a PAR of 3 (Fig. 8a, Fc_[fluorescein]_2__[fluorescein]_1_31) with excellent homogeneity and purity. Incubation of Fc conjugate 30 with a DBCO-rhodamine resulted in selective modification of the two phenyl-N_3_ to afford incorporation of two rhodamines per Fc protein (PAR 2) with high purity and homogeneity (Fig. 8b, Fc_[rhodamine]_2__[MeTz]_1_32). Incubation of Fc conjugate 30 with a TCO-biotin or a TCO-Cy5 selectively modified the MeTz handle to afford incorporation of a single biotin or a single Cy5 in the Fc scaffold (PAR 1), respectively, with almost no methyl-phenyl azide elimination in both cases (Fig. 8c, Fc_[PhN_3_]_2__[biotin]_1_33; and Fc_[PhN_3_]_2__[Cy5]_1_ S33, see ESI†). As for the mAb and Fab′ formats, three different mono-functional PARs could thus be obtained from a single pool of re-bridged Fc fragment and adequate click reaction selection. The sequential reactions of Fc conjugate 30 with DBCO-rhodamine and BCN-fluorescein afforded a bi-functional Fc-conjugate displaying two rhodamine and one fluorescein fluorophores, for a corresponding PAR of 2 + 1 (Fig. 8d, Fc_[rhodamine]_2__[fluorescein]_1_) with excellent homogeneity.

Only a few Fc-conjugates have recently been reported on literature thus far. They were generated either *via* recombinant methods,^54,55^ or after enzymatic modification of recombinant Fc.^56–58^ To the best of our knowledge, no site-selective chemical modification of a non-engineered Fc fragment has been described as yet. These pioneering reports confirm that the coupling of an Fc fragment to ligands can improve their half-life *in vivo*. One can anticipate that grafting an Fc fragment could also provide immune function to the resulting Fc–ligands conjugates, and that a similar strategy could be extended to Fc–protein conjugates. The re-bridging method we described here could be adapted/extended to the grafting of several copies of a single ligand or a combination of different ligands to an Fc scaffold to generate what we termed a “pseudo-antibody”, without requiring engineering. This approach could be coupled to a high-throughput process in order to provide numerous antibody biosimilars directed against targets for which no conventional antibody has been identified yet. We also highlight that PD-based linkers have been successfully applied on many antibodies with both cleavable and non-cleavable linkers being used to append various drugs (including relatively hydrophobic drugs) with success in various settings demonstrated, including serum stability and efficacy *in vitro* and *in vivo*.^59–65^ We are now investigating this strategy and believe that our re-bridging method applied to Fc fragments could boost the development and application of Fc-conjugates in the future.

## 5 Conclusion

In this article, we report a chemical method that enables the generation of either mAb-, Fab′- or Fc-conjugates with a controlled loading of mono or dual payloads, with high homogeneity and without requiring recombinant technologies. Based on the reduction and re-bridging of native proteins with a single trifunctional bridging linker, the method gives access to three different mono-functional PARs (1, 2, 3 for Fc and Fab′; and 2, 4 and 6 for a mAb) and a bi-functional PAR (2 + 1 for Fc and Fab′; 4 + 2 for mAb) for each protein scaffold it was applied to, only by varying the copper-free click reactions employed for model payload attachment. We also note that ELISA binding studies revealed that mAb conjugate 17 and Fab′ conjugate 24 displayed similar binding to HER2 when compared to native trastuzumab mAb and trastuzumab Fab controls (see ESI,† Section 5). The modularity afforded by the method regarding the type and the range of accessible PARs, as well as the range of antibody formats it is compatible with, has hitherto not been achieved. Not requiring recombinant proteins to apply the method is an additional asset regarding modularity and accessibility, but the method should be applicable to recombinant proteins as well. The homogeneity and number of payloads grafted on an antibody impacts pharmacokinetic and therapeutic effects, but no consensus exist regarding an optimal PAR – it seems that a case-by-case optimisation is required for each application. In this context, we believe the presented method holds a great potential to facilitate and/or enable the payload/antibody ratio optimisation of antibody-, Fab′- or Fc-conjugates before potential high-cost scale-up production.

## Conflicts of interest

There are no conflicts to declare.

## Data availability

The ESI† is available and it contains all the experimental data.

## Author contributions

F. T. prepared the antibody fragments. F. T. synthesized the small molecules. F. T. and L. N. C. R. generated the protein conjugates. F. T. and L. N. C. R. analysed the protein and/or protein conjugates by LC-MS. F. T. analysed the protein and/or protein conjugates by SDS-PAGE. F. T., J. R. B. and V. C. devised the study. F. T. and V. C. co-wrote the manuscript. All authors read and approved the final manuscript.

## Supplementary Material

SC-014-D2SC06318B-s001
