# Supplementary material for: Enabling the formation of native mAb, Fab′ and Fc-conjugates using a bis-disulfide bridging reagent to achieve tunable payload-to-antibody ratios (PARs)
Source: Chem Sci. 2023 Mar 9;14(14):3752–62. doi: 10.1039/d2sc06318b (PMC10074397; doi:10.1039/d2sc06318b)
Supplement: SC-014-D2SC06318B-s001 [file SC-014-D2SC06318B-s001.pdf]

Electronic Supplementary Material (ESI) for Chemical Science.
This journal is © The Royal Society of Chemistry 2023

ESI: Enabling the formation of native mAb, Fab' and Fc-conjugates using a bis-disulfide bridging reagent to achieve tunable payload-to-antibody ratios (PARs)

Fabien Thoreau,[a]* Léa N. C. Rochet,[a] James R. Baker,[a]* Vijay Chudasama[a]*

[a]: Department of Chemistry, University College London, 20 Gordon Street, London, WC1H 0AJ, UK

*: Correspondence: v.chudasama@ucl.ac.uk, j.r.baker@ucl.ac.uk, fabien.thoreau@outlook.fr

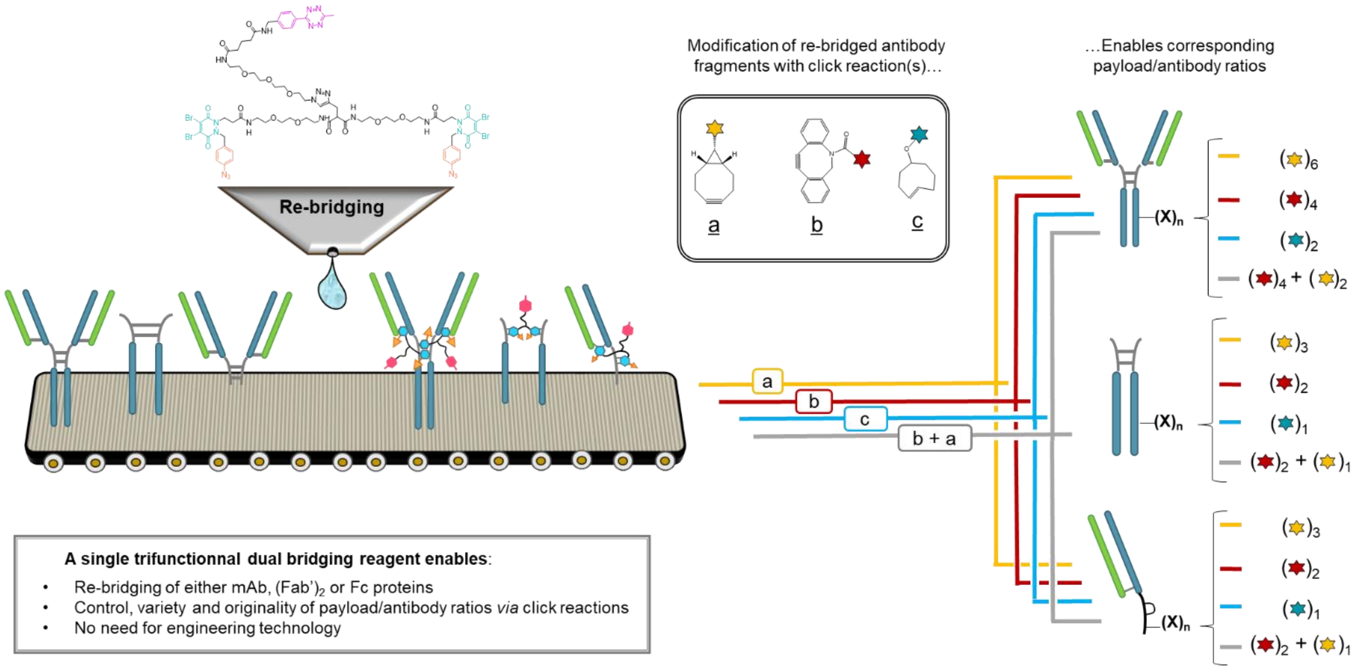

1. Synthetic chemistry section

1.1 General experimental details for synthetic chemistry

Chemicals were purchased from Sigma-Aldrich, Santa Cruz Biotechnology, AlfaAesar or Axispharm, and were used as received unless otherwise stated. Solvents were used as supplied. All reactions were monitored by thin-layer chromatography (TLC) on pre-coated silica gel plates. Flash column chromatography was carried out with either pre-loaded Biotage® SNAP column chromatography cartridges or pre-loaded GraceResolvTM flash cartridges on a Biotage® Isolera Spektra One flash chromatography system. All reaction mixtures were stirred magnetically unless stated otherwise. All reactions involving moisture sensitive compounds or procedures were carried out in flame-dried flask under an atmosphere of argon. Room temperature (RT) is defined as 16-23 °C. Reactions at 0 °C were cooled with an ice/water bath. Removal of solvent and concentration *in vacuo* was carried out on a Büchi rotary evaporator followed by evaporation under high vacuum.

^{1}H NMR spectra were obtained at 400, 500, 600 or 700 MHz. ^{13}C NMR spectra were obtained at 100, 125, 150 or 175 MHz. All results were obtained using Bruker NMR instruments, the models are as follows: Avance III 400, Avance 500, Avance III 600, Avance Neo 700. The chemical shifts (δ) for ^{1}H and ^{13}C are quoted relative to residual signals of the solvent on the ppm scale. ^{1}H NMR peaks are reported as singlet (s), doublet (d), triplet (t), quint. (quintet), m (multiplet), br. (broad), dd (doublet of doublet). Coupling constants (J values) are reported in Hertz (Hz) and are H-H coupling constants unless otherwise stated. In the case of amide rotamers, and when possible, only the major rotamer has been assigned for chemical shifts, and areas underneath all rotameric peaks have been considered for integration calculations. Peak assignments were carried out with the aid of ^{1}H COSY and ^{1}H–^{13}C HSQC experiments where necessary. Infrared spectra were obtained on a Perkin Elmer Spectrum 100 FTIR Spectrometer operating in ATR mode. Infra-red spectra were recorded on a Bruker ALPHA FT-IR spectrometer operating in ATR mode, with frequencies given in reciprocal centimetres (cm^{-1}). Melting points were taken on a Gellenkamp apparatus and are uncorrected. High and low resolution mass spectra were recorded on a VG70 SE mass spectrometer, operating in modes ESI, EI, or CI (+ or -) depending on the sample, at the Department of Chemistry, University College London.

1.2 Synthetic chemistry protocols & NMR

Compound 1 - 1-azido-4-methylbenzene[1]

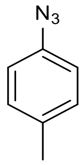

To a solution of p-toluidine (5.0 g, 46.66 mmol) in 2m HCl (50 mL) at −5 °C was added slowly a solution of sodium nitrite (3.75 g, 54.35 mmol) in H_2O (10 mL) over 5 min making sure that the internal temperature did not rise above 0 °C. After completion of addition, the reaction mixture was stirred at −5 °C for 5 min to form a diazonium salt. Then urea (0.32 g, 5.33 mmol) was added to neutralise the diazonium salt solution. Following this, the diazonium salt solution was added to a solution of sodium azide (6 g, 92.29 mmol) and sodium acetate (11.5 g, 140.0 mmol) in 50 mL of H_2O at 0 °C over 5 min. The mixture was stirred for 2 h at 0 °C. The mixture was extracted into Et_2O (2 × 100 mL), the combined organic layers dried ($MgSO_4$) and concentrated under vacuum to afford 1-azido-4-methylbenzene **1** (5.91 g, 44.39 mmol, 95 %) as a yellow oil. ^{1}H NMR (600 MHz, $CDCl_3$) 7.15 (d, J = 8.4 Hz, 2H), 6.92 (d, J = 8.4 Hz, 2H), 2.32 (s, 3H); ^{13}C NMR (125 MHz, $CDCl_3$) 137.2 (C), 134.8 (C), 130.5 (CH), 119.0 (CH), 21.0 (CH_3).

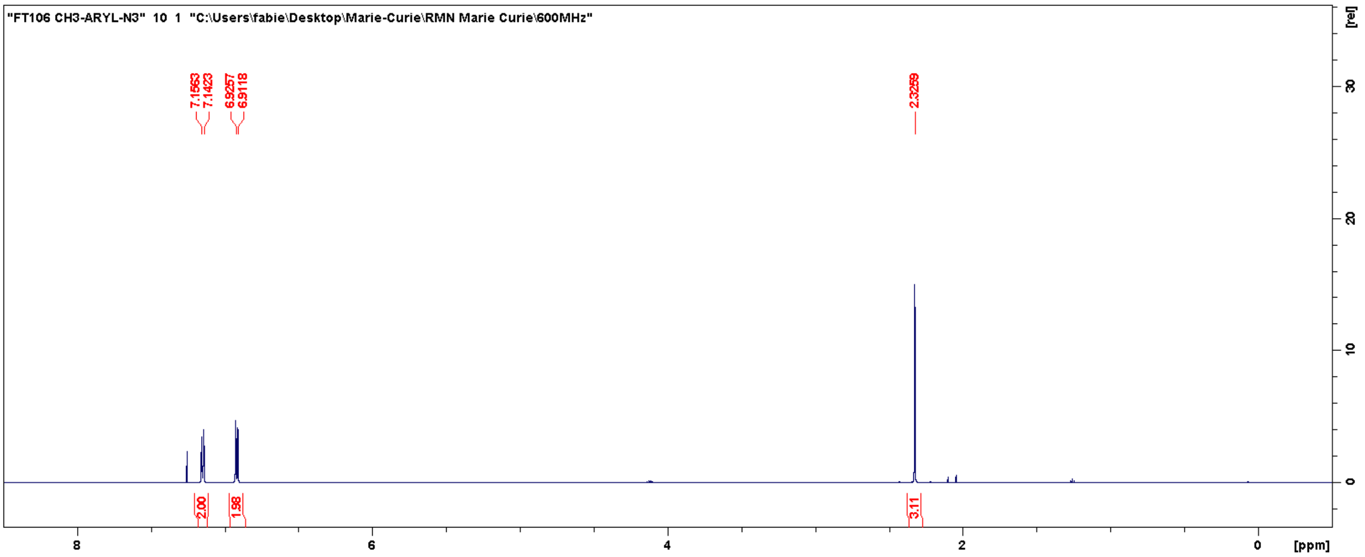

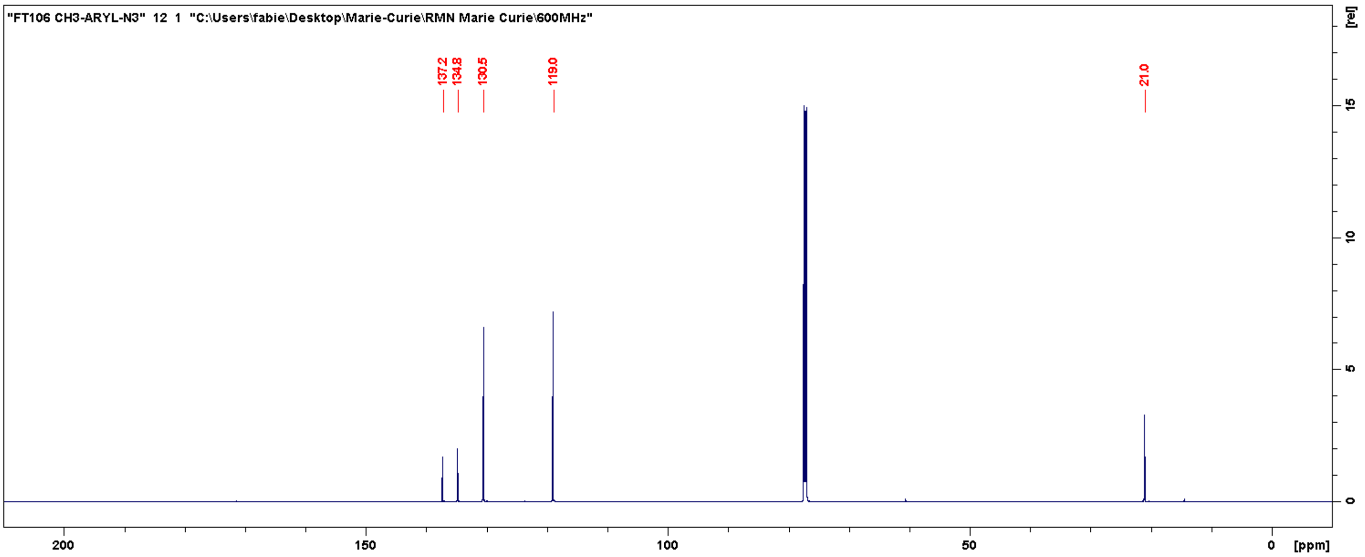

Compound 2 - 1-azido-4-(bromomethyl)benzene[1]

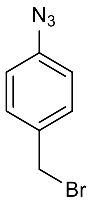

A solution of 1-azido-4-methylbenzene **1** (3 g, 22.6 mmol, 1 equiv.), N-bromosuccinimide (5.4 g, 30.5 mmol, 1.35 equiv.) and bis(isobutyronitrile) (1.13 g, 6.78 mmol, 0.3 equiv.) in dry benzene (30 mL) was heated under reflux under argon in the dark for 8 h. After this time, the mixture was poured onto H_2O (20 mL), extracted with Et_2O (2 × 20 mL), the combined organic layers dried ($MgSO_4$) and concentrated under vacuum. Purification by flash column chromatography (100 % cyclohexane) yielded 1-azido-4-(bromomethyl)benzene **2** (2.96 g, 13.96 mmol, 62 %) as a light brown solid: ^{1}H NMR (600 MHz, $CDCl_3$) 7.38 (d, J = 8.4 Hz, 2H), 7.00 (d, J = 8.4 Hz, 2H), 4.48 (s, 2H); ^{13}C NMR (150 MHz, $CDCl_3$) 140.3 (C), 134.6 (C), 130.7 (CH), 119.5 (CH), 33.1 (CH_2); IR (solid) 2107, 1607, 1505 cm^{-1}; GC-MS : theoretical = 209.97, 210.97, 211.97, 212.97; experimental = 211.08, 212.09, 213.07, 214.08.

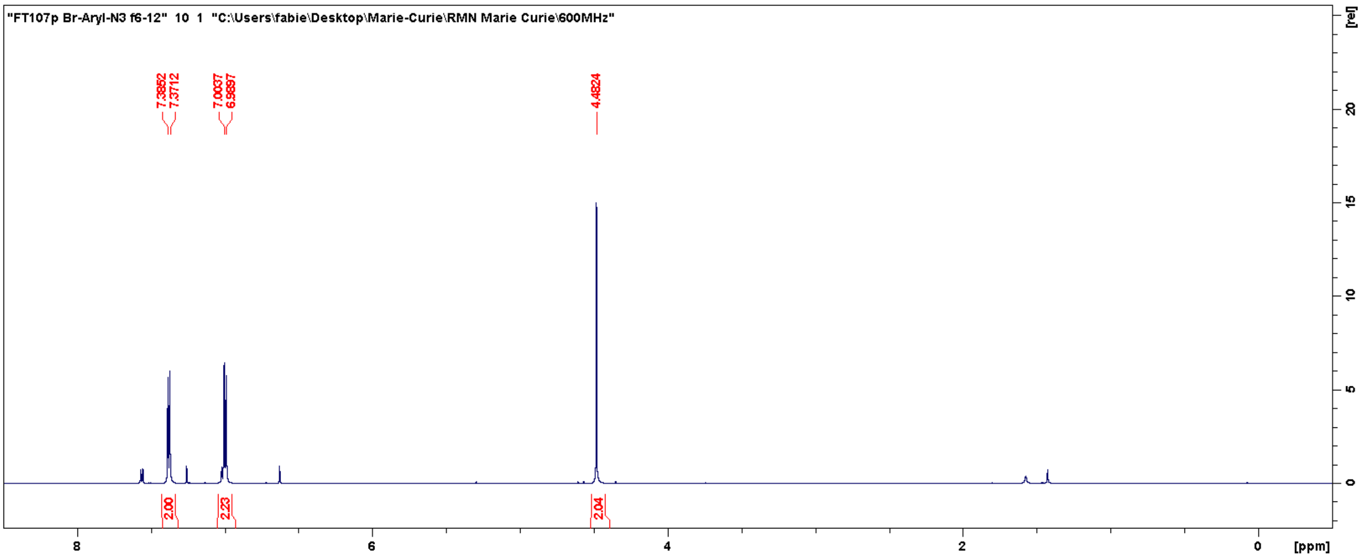

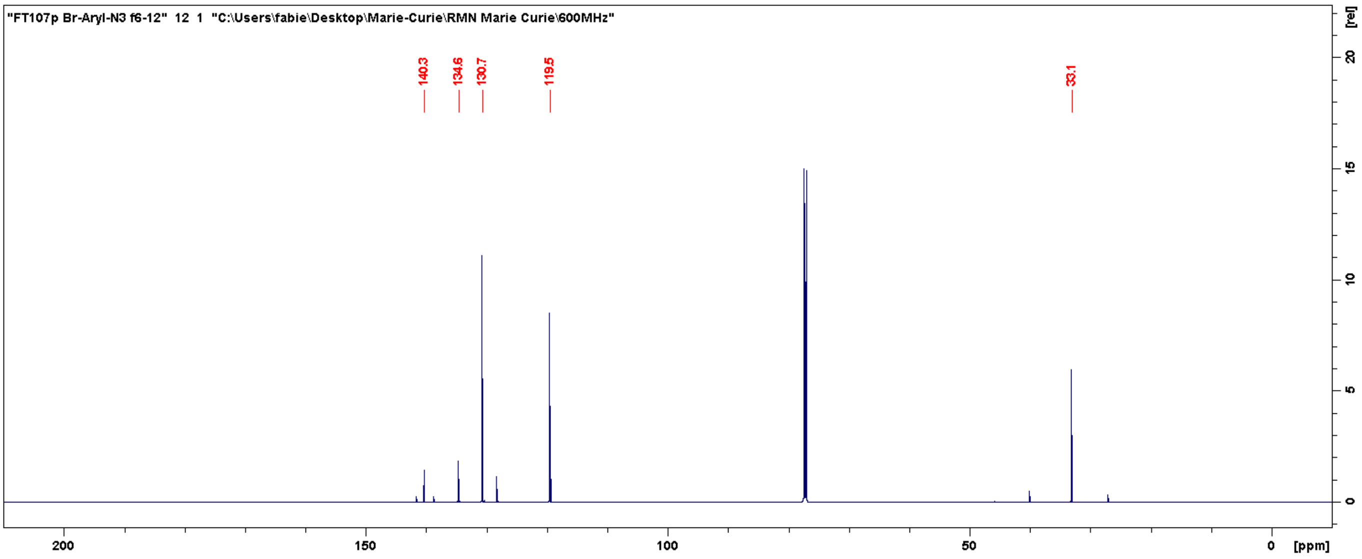

Compound 3 - *di-tert-butyl 1-(4-azidobenzyl)hydrazine-1,2-dicarboxylate*

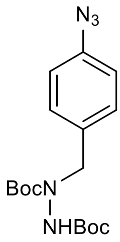

To a solution of di-*tert*-butyl hydrazine-1,2-dicarboxylate (2.17 g, 9.35 mmol, 1 equiv.) in a mixture of toluene (25 mL) and 5 % aq. NaOH (25 mL) were added tetra-*n*-butylammonium bromide (143 mg, 0.35 mmol, 0.025 equiv.) and 1-azido-4-(bromomethyl)benzene **2** (2.96 g, 14.0 mmol, 1.5 equiv.). The reaction mixture was stirred at 20 °C for 48 h. After this time, the reaction mixture was diluted with H_2O (20 mL) and extracted with EtOAc (3 × 20 mL). The combined organic layers were washed with brine (15 mL), dried ($MgSO_4$), and concentrated under vacuum. Purification by flash column chromatography (20 % EtOAc in cyclohexane) yielded di-tert-butyl 1-(4-azidobenzyl)hydrazine-1,2-dicarboxylate **3** (3.27 g, 9.0 mmol, 96 %) as a thick orange oil (presence of rotamers): ^{1}H NMR (600 MHz, $CDCl_3$) 7.27 (br. s, 2H), 6.98 (d, *J* = 7.9 Hz, 2H), 4.59 (br. s, 2H), 1.47 – 1.42 (m, 18H); ^{13}C NMR (150 MHz, $CDCl_3$) 155.5 (C), 155.2 (C), 139.4 (C), 134.1 (C), 130.2 (CH), 129.8 (CH), 119.2 (CH), 81.6 (C), 81.4 (C), 53.6 (CH_2), 52.5 (CH_2), 28.3 (CH_3), 27.0 (CH_3); IR (thin film) 3316, 2978, 2107, 1703, 1150 cm^{-1}; HRMS (ESI) calcd for $[C_{17}H_{25}N_5O_4+H]+$: 364.1979, observed: 364.1979.

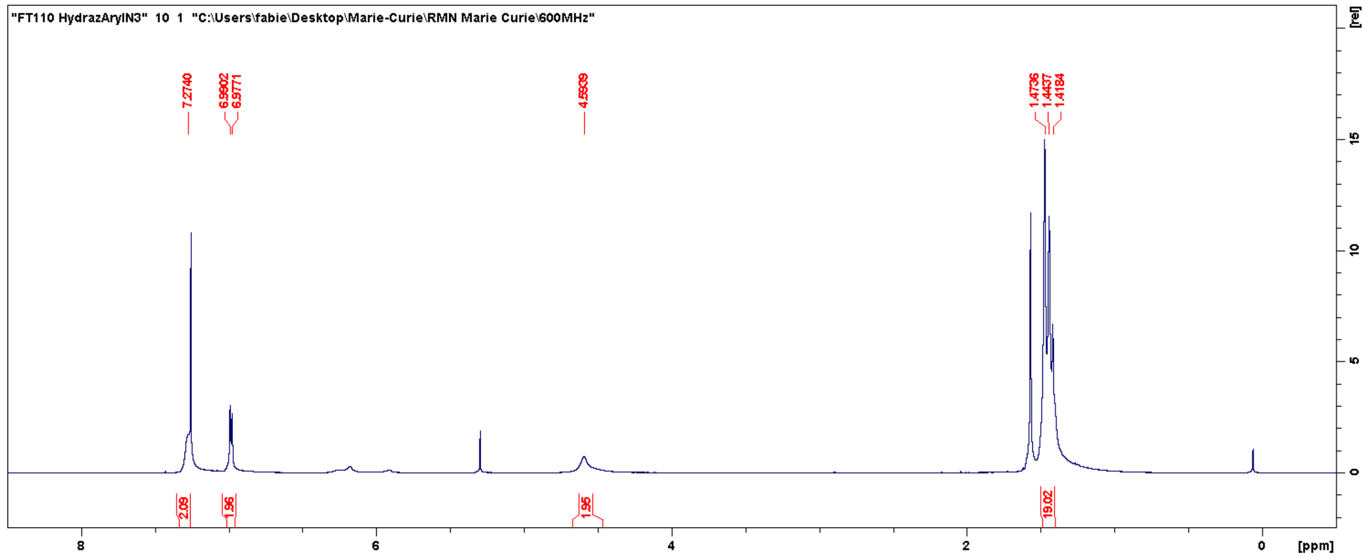

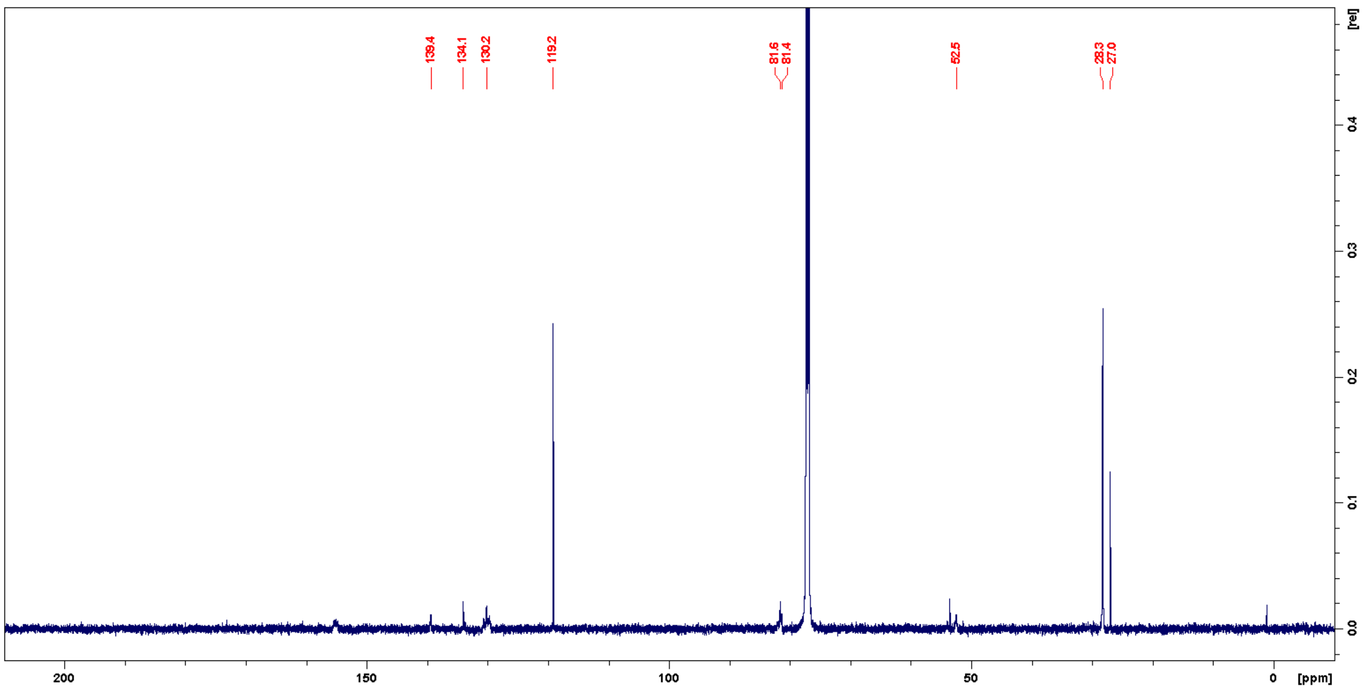

Compound 4 - di-tert-butyl 1-(4-azidobenzyl)-2-(3-(tert-butoxy)-3-oxopropyl)hydrazine-1,2-dicarboxylate

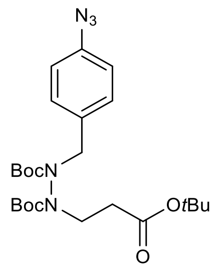

To a solution of di-tert-butyl 1-(4-azidobenzyl)hydrazine-1,2-dicarboxylate **3** (2.00 g, 5.51 mmol, 1 equiv.) in *tert*-BuOH (30 mL) was added 0.4 mL of NaOH (10 % w/w) and reaction mixture was stirred for 10 min at room temperature. After this time, *tert*-butyl acrylate (2.4 mL, 16.5 mmol, 3 equiv.) was added to the solution and the reaction was stirred at 65 °C for 16 h. Crude was concentrated under vacuum, solubilised in EtOAc (60 mL), washed with H_2O (3 x 30 mL) , dried Na_2SO_4 and concentrated under vacuum. Purification by flash column chromatography (0 to 40 % of EtOAc in cyclohexane) yielded di-tert-butyl 1-(4-azidobenzyl)-2-(3-(tert-butoxy)-3-oxopropyl)hydrazine-1,2-dicarboxylate **4** (1.14 g, 2.32 mmol, 42 %) as a thick orange oil (presence of rotamers): ^{1}H NMR (600 MHz, $CDCl_3$) 7.31 (m, 2H), 6.97 (d, J = 8.4 Hz, 2H), 4.89 - 4.20 (m, 2H), 3.67 - 3.31 (m, 2H), 2.52 - 1.95 (m, 2H), 1.48 - 1.31 (m, 27H); ^{13}C NMR (150 MHz, $CDCl_3$) 171.1 (C), 155.0 (C), 154.6 (C), 139.6 (C), 133.6 (C), 131.3 (CH), 119.1 (CH), 81.6 (C), 81.2 (C), 80.8 (C), 52.1 (CH_2), 46.2 (CH_2), 34.0 (CH_2), 28.4 (CH_3), 28.3 (CH_3), 28.2 (CH_3); IR (thin film) 2977, 2932, 2111, 1706, 1366, 1147 cm^{-1}, HRMS (ESI) calcd for $[C_{24}H_{37}N_5O_6+H]^+$: 492.2817, observed: 492.2813.

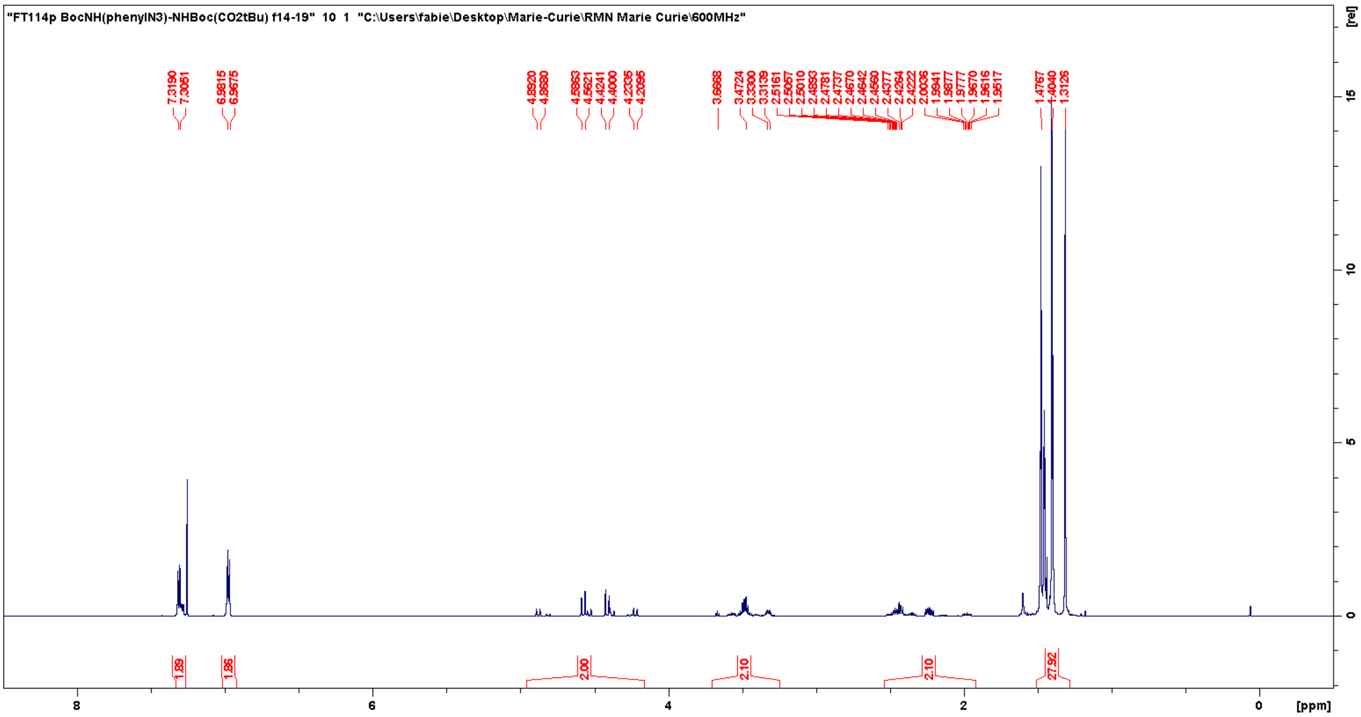
"FT114p BocNH(phenylN3)-NHBoc(CO2tBu) f14-19" 10 1 "C:\Users\fabie\Desktop\Marie-Curie\RMN Marie Curie\600MHz"
7.3190
7.3051
6.9815
6.9675
4.8920
4.8830
4.5863
4.5621
4.4241
4.4000
4.2335
4.2095
3.6668
3.4724
3.3300
3.3139
2.5161
2.5067
2.5010
2.4893
2.4781
2.4737
2.4670
2.4642
2.4560
2.4377
2.4264
2.4222
2.0036
1.9941
1.9877
1.9777
1.9670
1.9616
1.9517
1.4767
1.4040
1.3126
1.89
1.86
2.00
2.10
2.10
27.92
[rel]
[ppm]

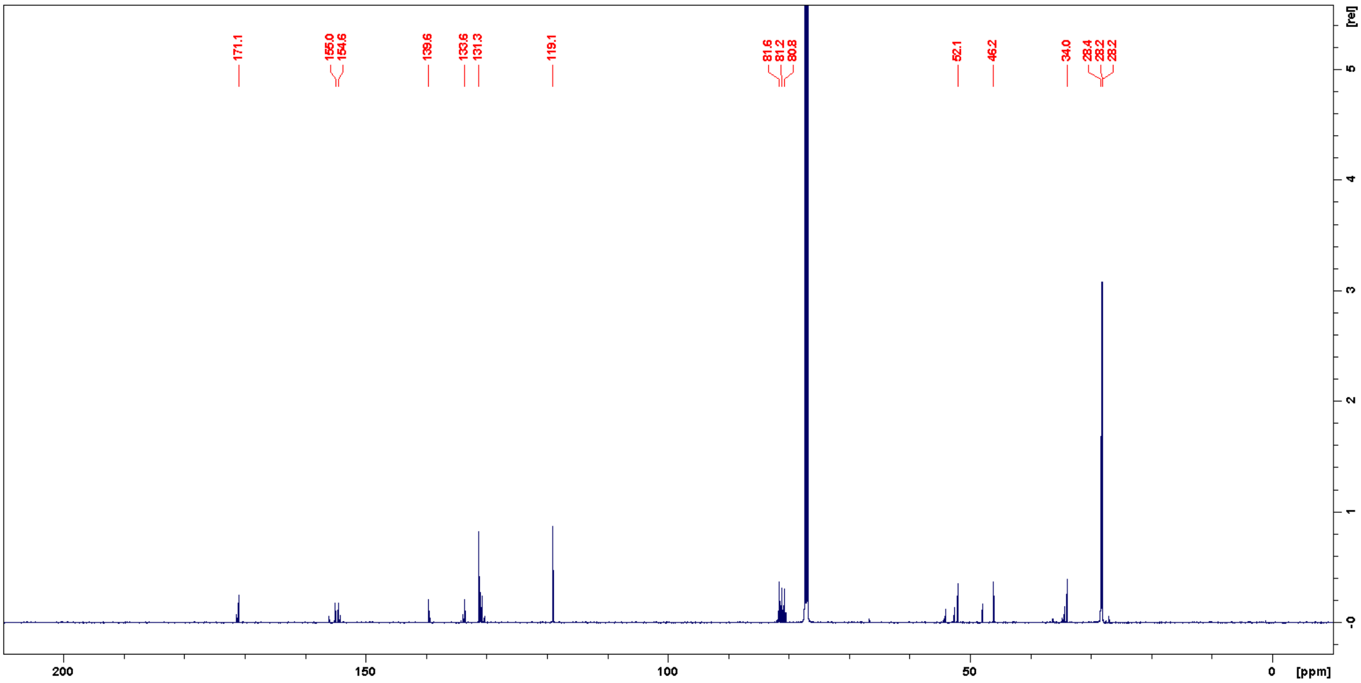
171.1
155.0
154.6
139.6
133.6
131.3
119.1
81.6
81.2
80.8
52.1
46.2
34.0
28.4
28.2
28.2
[rel]
[ppm]

Compound 5 - 3-(2-(4-azidobenzyl)-4,5-dibromo-3,6-dioxo-3,6-dihydropyridazin-1(2H)-yl)propanoic acid

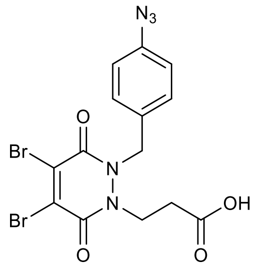

Dibromomaleic acid (826 mg, 3.016 mmol, 1.53 equiv.) was dissolved in AcOH (200 mL) and heated under reflux for 30 min. After this time, di-tert-butyl 1-(4-azidobenzyl)-2-(3-(tert-butoxy)-3-oxopropyl)hydrazine-1,2-dicarboxylate **4** (1.14 g, 2.32 mmol, 1 equiv.) was added and the resultant mixture was stirred under reflux for a further 12 h (solution turned brown). The solvent was removed under vacuum by co-evaporation with toluene and the crude residue purified by flash column chromatography (0 to 100 % EtOAc (1 % AcOH) in cyclohexane) to yield 3-(2-(4-azidobenzyl)-4,5-dibromo-3,6-dioxo-3,6-dihydropyridazin-1(2H)-yl)propanoic acid **5** (800 mg, 1.69 mmol, 73 %) as a yellow solid: ^{1}H NMR (400 MHz, $CDCl_3$) δ 11.09 (s, 7H), 7.20 (d, *J* = 8.5 Hz, 2H), 6.98 (d, *J* = 8.5 Hz, 2H), 5.32 (s, 2H), 4.26 (t, *J* = 7.1 Hz, 2H), 2.58 (t, *J* = 7.1 Hz, 2H); ^{13}C NMR (150 MHz, $CDCl_3$) 175.7 (C), 154.1 (C), 153.4 (C), 140.7 (C), 136.2 (C), 130.7 (C), 128.7 (CH), 120.0 (CH), 50.7 (CH_2), 43.3 (CH_2), 31.4 (CH_2); IR (thin film) 2968, 2105, 1724, 1365, 1152 cm^{-1}; HRMS (ESI) calcd for $[C_{14}H_{11}Br_2N_5O_4\text{-H}]^-$: 469.9106, observed: 469.9105

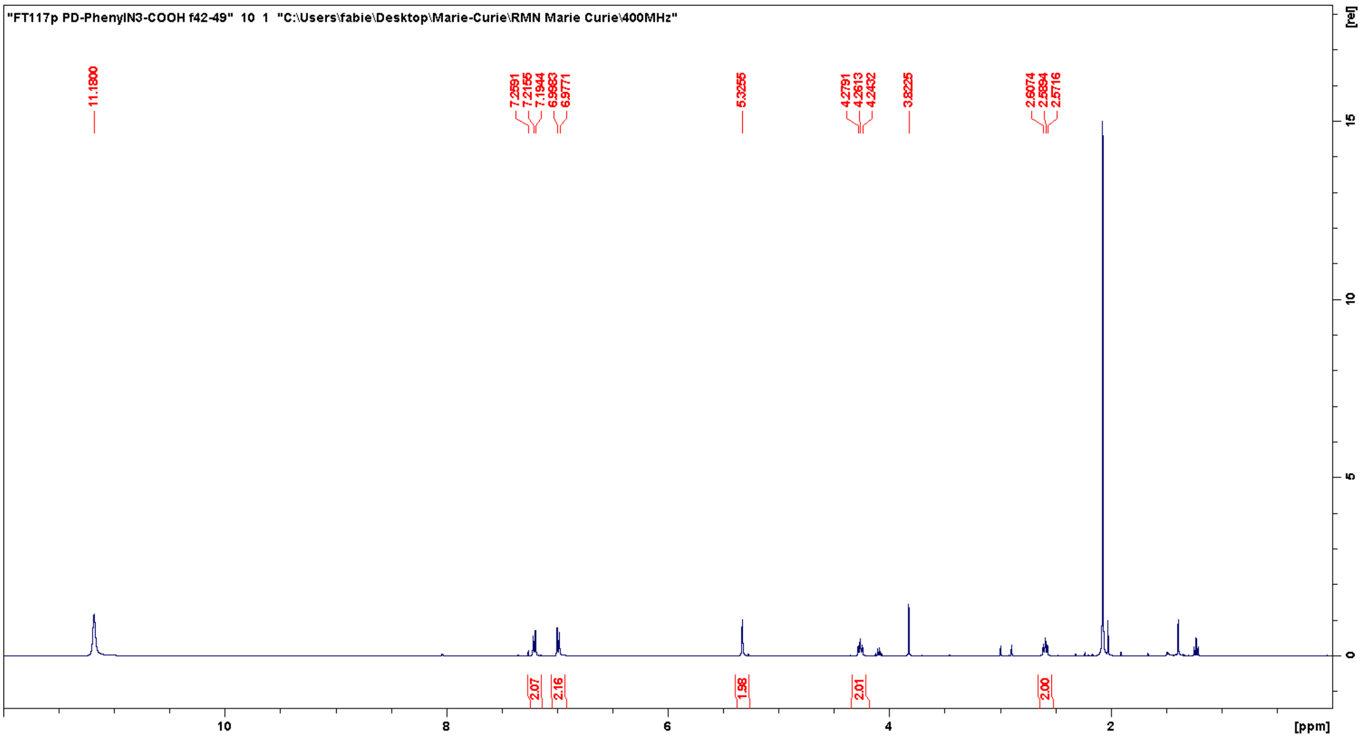

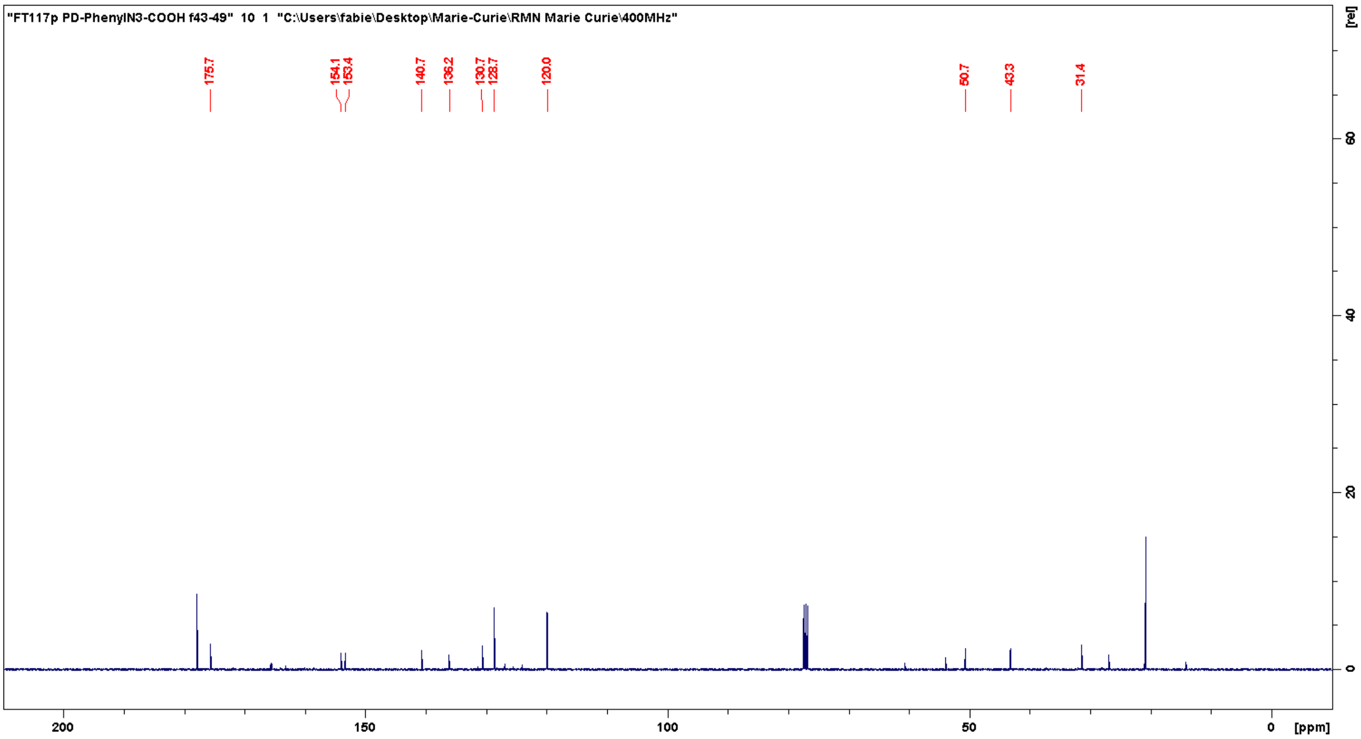

Compound 6 - 2,5-dioxopyrrolidin-1-yl 3-(2-(4-azidobenzyl)-4,5-dibromo-3,6-dioxo-3,6-dihydropyridazin-1(2H)-yl)propanoate

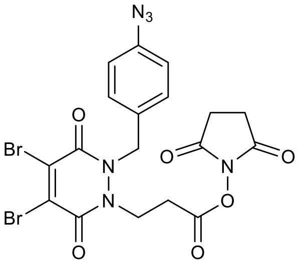

To a solution of 3-(2-(4-azidobenzyl)-4,5-dibromo-3,6-dioxo-3,6-dihydropyridazin-1(2H)-yl)propanoic acid **5** (400 mg, 849 µmol, 1 equiv.) in DCM (100 mL) at 0 °C was added EDC.HCl (327 mg, 1.70 mmol, 2 equiv.). The solution was stirred at 0 °C for 30 min. Following this, NHS was added (196 mg, 1.70 mmol, 2 equiv.) and the reaction was stirred at room temperature for a further 16 h. The solvent was removed under vacuum and the crude residue purified by flash column chromatography (10 to 100 % EtOAc in cyclohexane) to yield 2,5-dioxopyrrolidin-1-yl 3-(2-(4-azidobenzyl)-4,5-dibromo-3,6-dioxo-3,6-dihydropyridazin-1(2H)-yl)propanoate **6** (145 mg, 30 %) as light yellow crystals: ^{1}H NMR (600 MHz, $CDCl_3$) δ 7.21 (d, *J* = 8.4 Hz, 2H), 7.02 (d, *J* = 8.4 Hz, 2H), 5.32 (s, 2H), 4.31 (t, *J* = 7.1 Hz, 2H), 2.86 (t, *J* = 7.1 Hz, 2H), 2.84 (s, 4H); ^{13}C NMR (150 MHz, $CDCl_3$) 168.8 (C), 166.1 (C), 154.0 (C), 153.3 (C), 140.9 (C), 136.6 (C), 136.0 (C), 130.6 (C), 128.8 (CH), 120.2 (CH), 50.8 (CH_2), 42.9 (CH_2), 28.8 (CH_2), 25.7 (CH_2); HRMS (ESI) calcd for $[C_{18}H_{14}Br_2N_6O_6+H]^+$: 568.9414, observed: 568.9426.

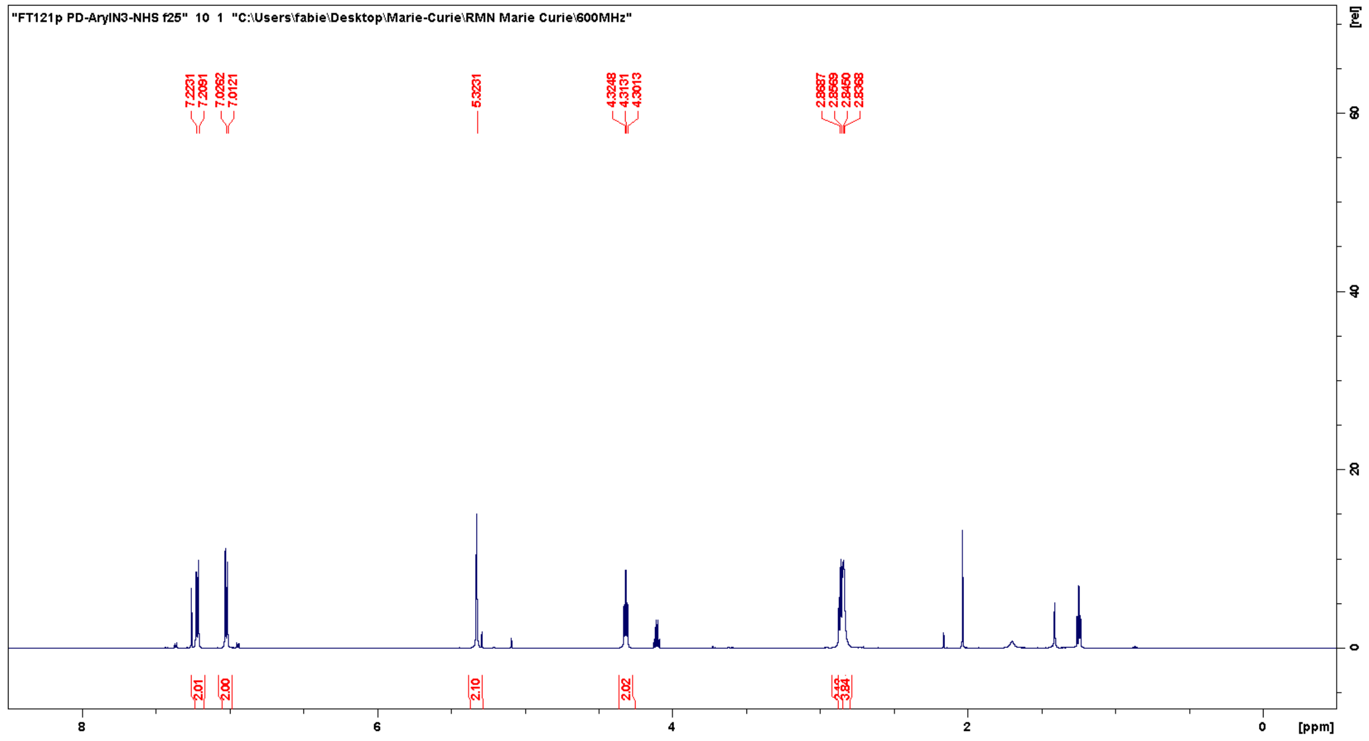
"FT121p PD-ArylN3-NHS f25" 10 1 "C:\Users\fabie\Desktop\Marie-Curie\RMN Marie Curie\600MHz"
7.2231
7.2091
7.0262
7.0121
5.3231
4.3248
4.3131
4.3013
2.8687
2.8569
2.8450
2.8368
2.01
2.00
2.10
2.02
3.94
[ppm]
[rel]

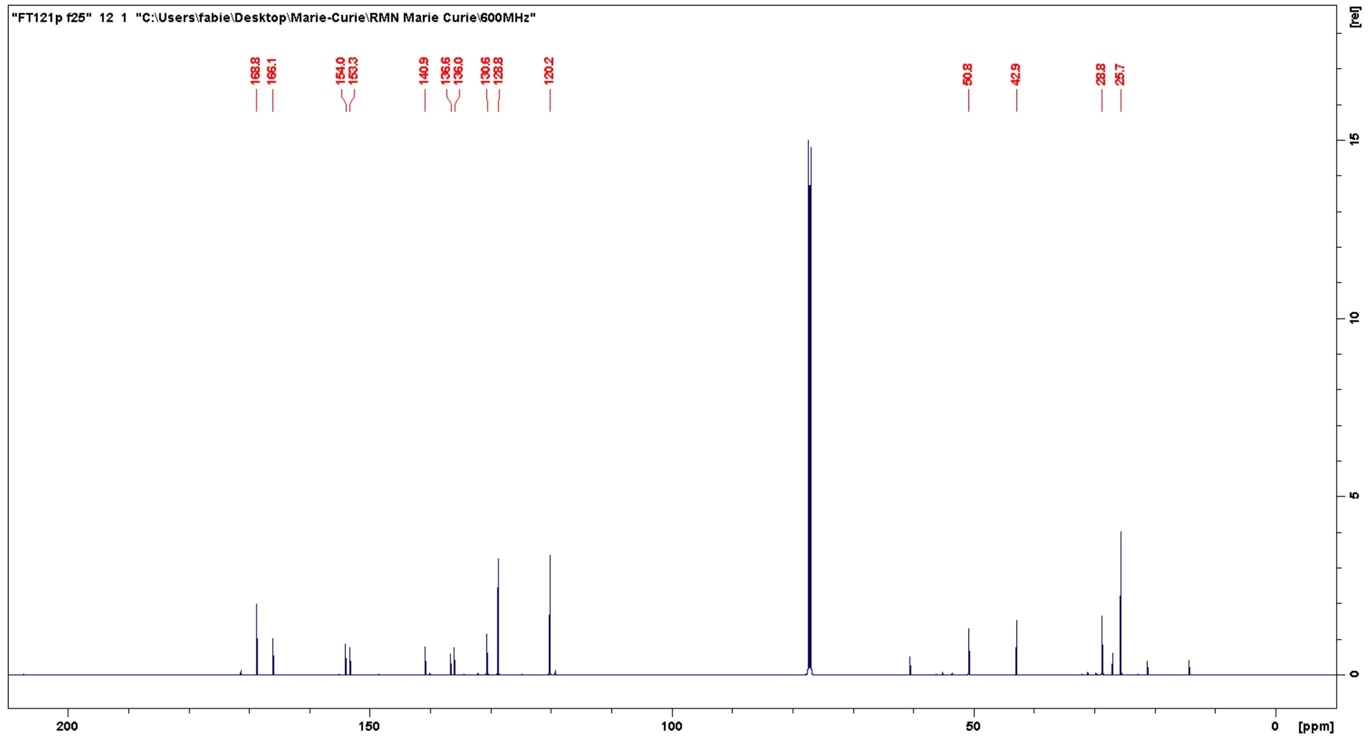
"FT121p f25" 12 1 "C:\Users\fabie\Desktop\Marie-Curie\RMN Marie Curie\600MHz"
168.8
166.1
154.0
153.3
140.9
136.6
136.0
130.6
128.8
120.2
50.8
42.9
28.8
25.7
[ppm]
[rel]

Compound 7 - tert-butyl (4-cyanobenzyl)carbamate[2]

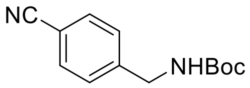

Boc_2O (10.7 g, 49.03 mmol, 1.1 equiv.) was dissolved in 100 mL of water. NaOH (5.4 g, 135 mmol, 2.8 equiv.) and 4-(aminomethyl) benzonitrile hydrochloride (7.5 g, 44.48 mmol, 1 equiv.) were added respectively and the solution was stirred overnight at room temperature. The white precipitate was isolated by centrifugation and dried using rotary evaporation, yielding 8.97 g of compound **7** as a white solid (38.62 mmol, 79 %): ^{1}H NMR (600 MHz, $CDCl_3$) δ 7.62 (d, *J* = 8.2 Hz, 2H), 7.38 (d, *J* = 8.2 Hz, 2H), 4.97 (s, 1H), 4.37 (d, *J* = 6.0 Hz, 2H), 1.46 (s, 9H) ; ^{13}C NMR (150 MHz, $CDCl_3$) δ 156.0 (C), 144.7 (C), 132.6 (CH), 127.9 (CH), 119.0 (C), 111.2 (C), 80.2 (C), 44.3 (CH_2), 28.5 (CH_3).

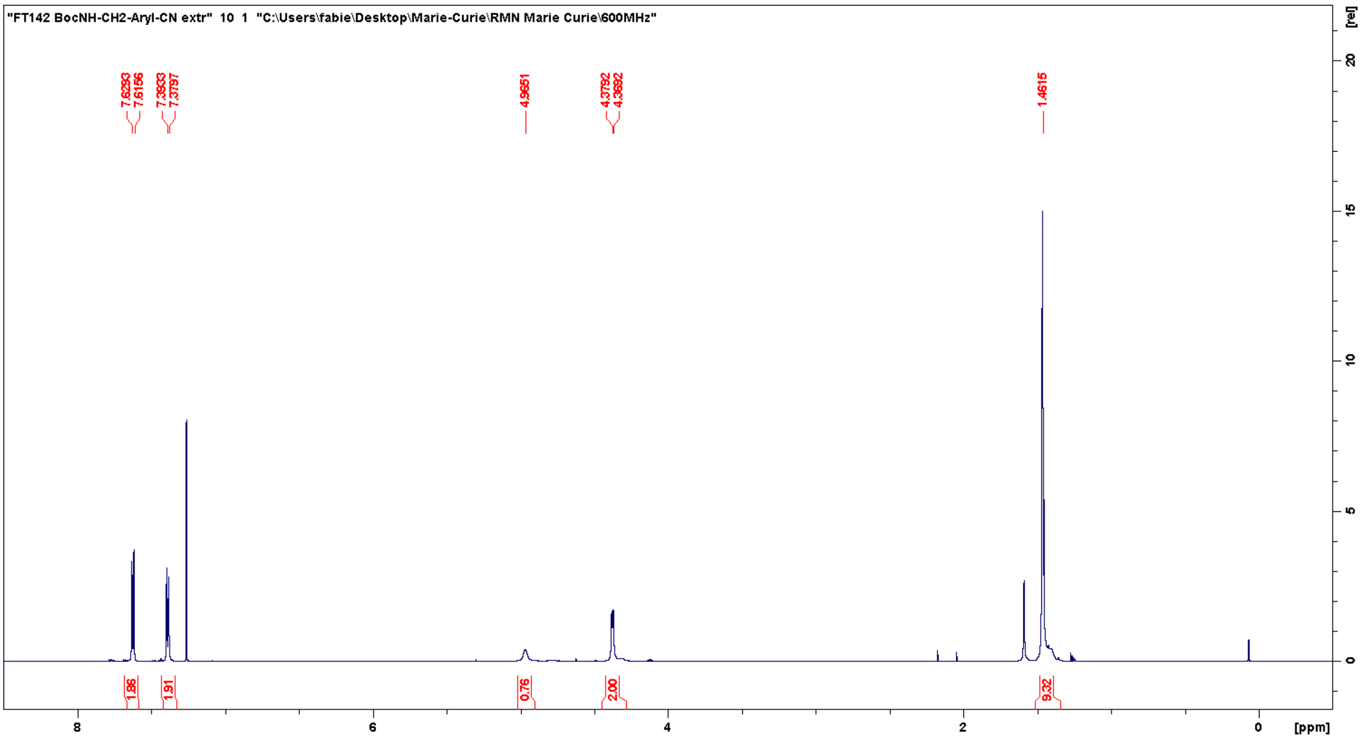

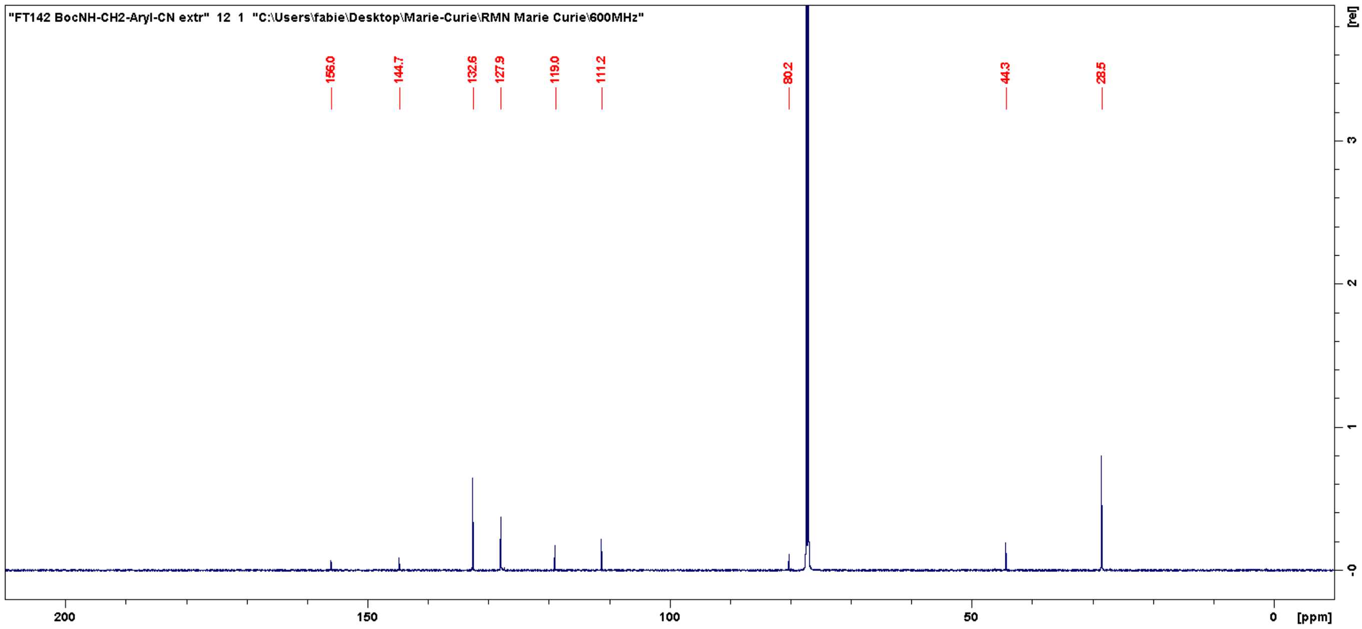

<u>Compound 8</u> - tert-butyl (4-(6-methyl-1,2,4,5-tetrazin-3-yl)benzyl)carbamate[3]

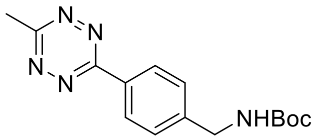

Tert-butyl (4-cyanobenzyl)carbamate **7** (5.0 g, 21.53 mmol, 1 equiv.), acetonitrile (5.6 mL, 107.65 mmol, 5 equiv.) and zinc triflate (1.96 g, 5.38 mmol, 0.25 equiv.) were added to 25 mL of dioxane in a round bottom flask under inert nitrogen atmosphere. Monohydrated hydrazine (53 mL, 1.08 mol, 50 equiv.) was added dropwise under heavy stirring, while maintaining room temperature in a water bath. After 5 minutes, temperature was adjusted to 80 °C and the mixture was stirred overnight to obtain a dihydrotetrazine containing reaction mixture (orange). For the oxidation step, 150 mL of a DCM/AcOH mixture (1:1, v:v) was prepared. While stirring, the dihydrotetrazine containing reaction mixture was added dropwise. Solid $NaNO_2$ (30 g, 434.81 mmol, 20 equiv.) was added portion wise over an hour (mixture get pink). The mixture was concentrated using rotary evaporation, re-dissolved in EtOAc, washed with $NaHCO_3$ (aq.) (added slowly), dried using $MgSO_4$ and concentrated under vacuum. The crude product was purified by flash chromatography (EtOAc 2 to 5 % in DCM), affording tert-butyl (4-(6-methyl-1,2,4,5-tetrazin-3-yl)benzyl)carbamate **8** as a pink solid (1.69 g, 5.60 mmol, 26 %): ^{1}H NMR (600 MHz, $CDCl_3$) δ 8.55 (d, *J* = 8.3, 2H), 7.50 (d, *J* = 8.3, 2H), 4.96 (NH, s, 1H), 4.43 (d, *J* = 5.9, 2H), 3.10 (s, 3H), 1.48 (s, 9H) ; ^{13}C NMR (150 MHz, CD_3OD) δ 167.4 (C), 164.1 (C), 156.1 (C), 144.1 (C), 130.9 (C), 128.4 (CH), 128.2 (CH), 80.0 (C), 44.5 (CH_2), 28.5 (CH_3), 21.1 (CH_3).

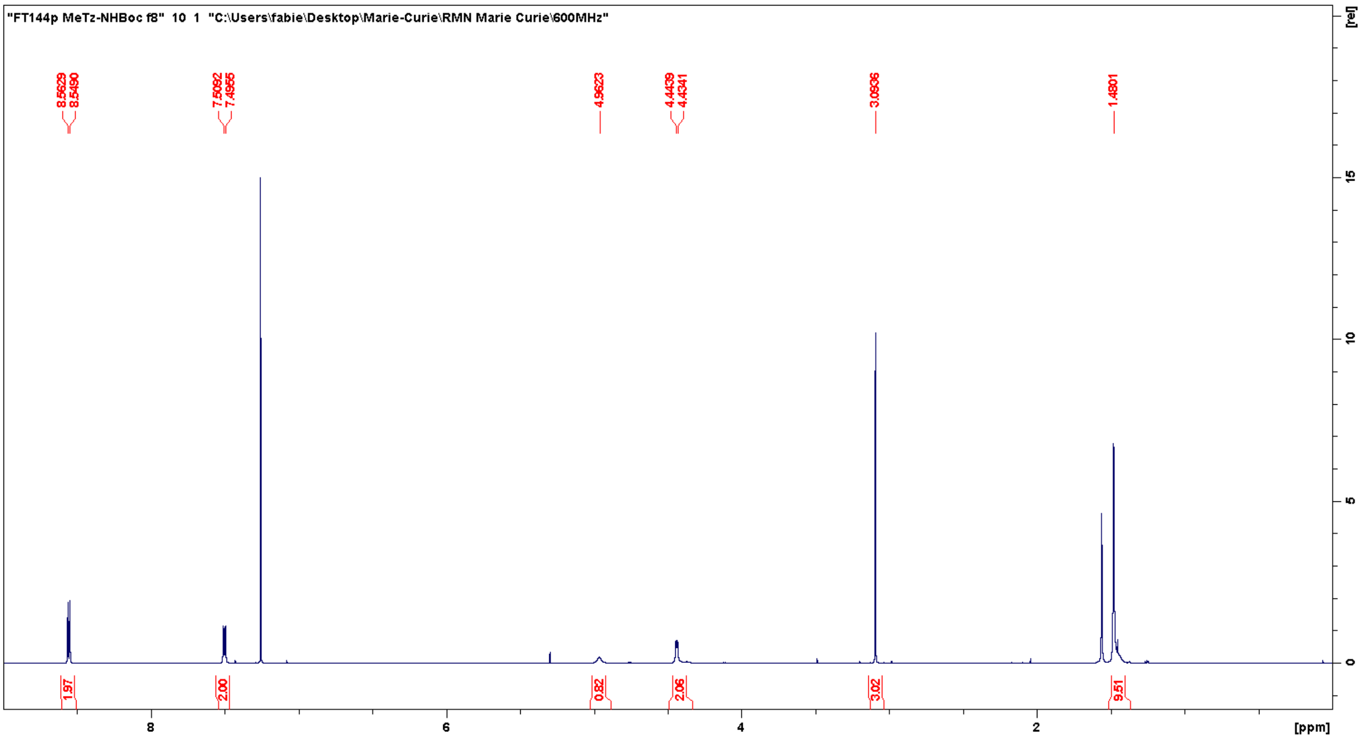

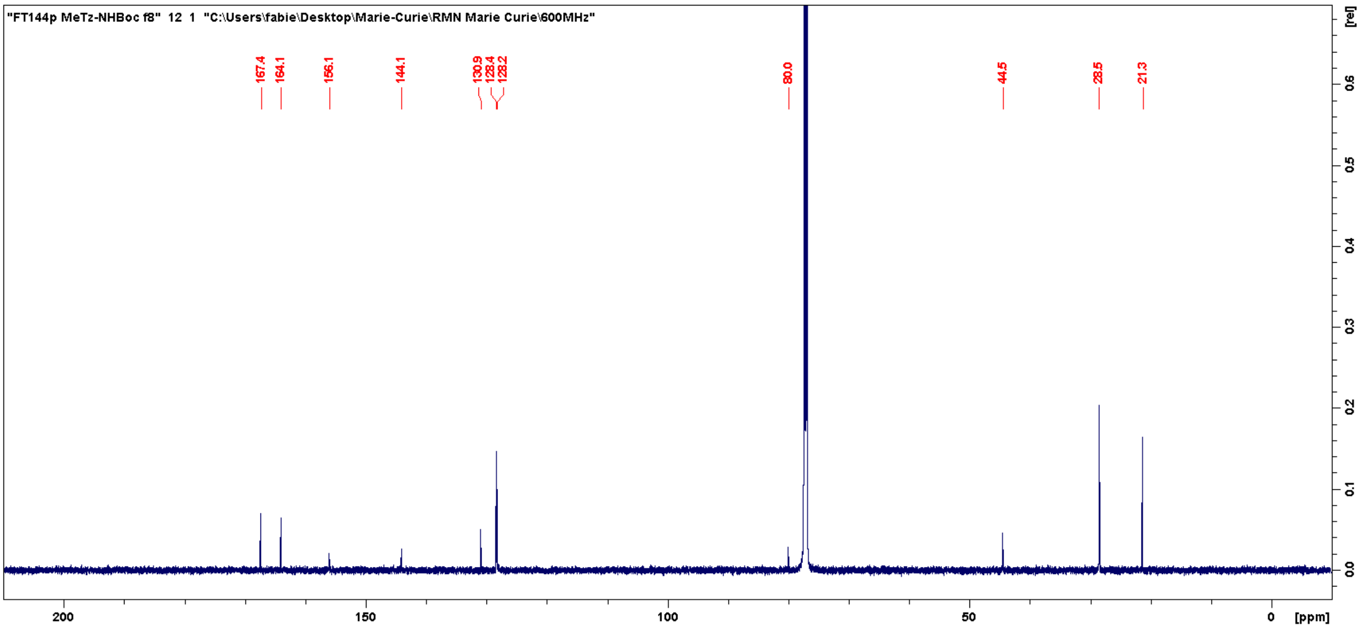

<u>Compound 9</u> - (4-(6-methyl-1,2,4,5-tetrazin-3-yl)phenyl)methanamine[3]

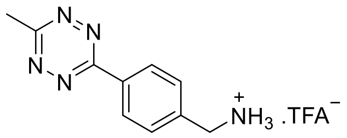

The N-Boc protected tetrazine **8** (1.69 g, 5.60 mmol, 1 equiv.) was dissolved in a DCM/TFA solution (1/1) and stirred 2 h, at room temperature. After this time, the reaction mixture was concentrated under vacuum resulting in (4-(6-methyl-1,2,4,5-tetrazin-3-yl)phenyl)methanamine **9** (1.76 g, quantitative) as a pink TFA salt: ^{1}H NMR (600 MHz, CD_3OD) δ 8.63 (d, *J* = 8.5, 2H), 7.71 (d, *J* = 8.5, 2H), 4.26 (br s, 2H), 3.06 (s, 3H) ; ^{13}C NMR (150 MHz, CD_3OD) δ 169.1 (C), 164.9 (C), 138.7 (C), 134.4 (C), 130.8 (CH), 129.4 (CH), 43.9 (CH_2), 21.1 (CH_3).

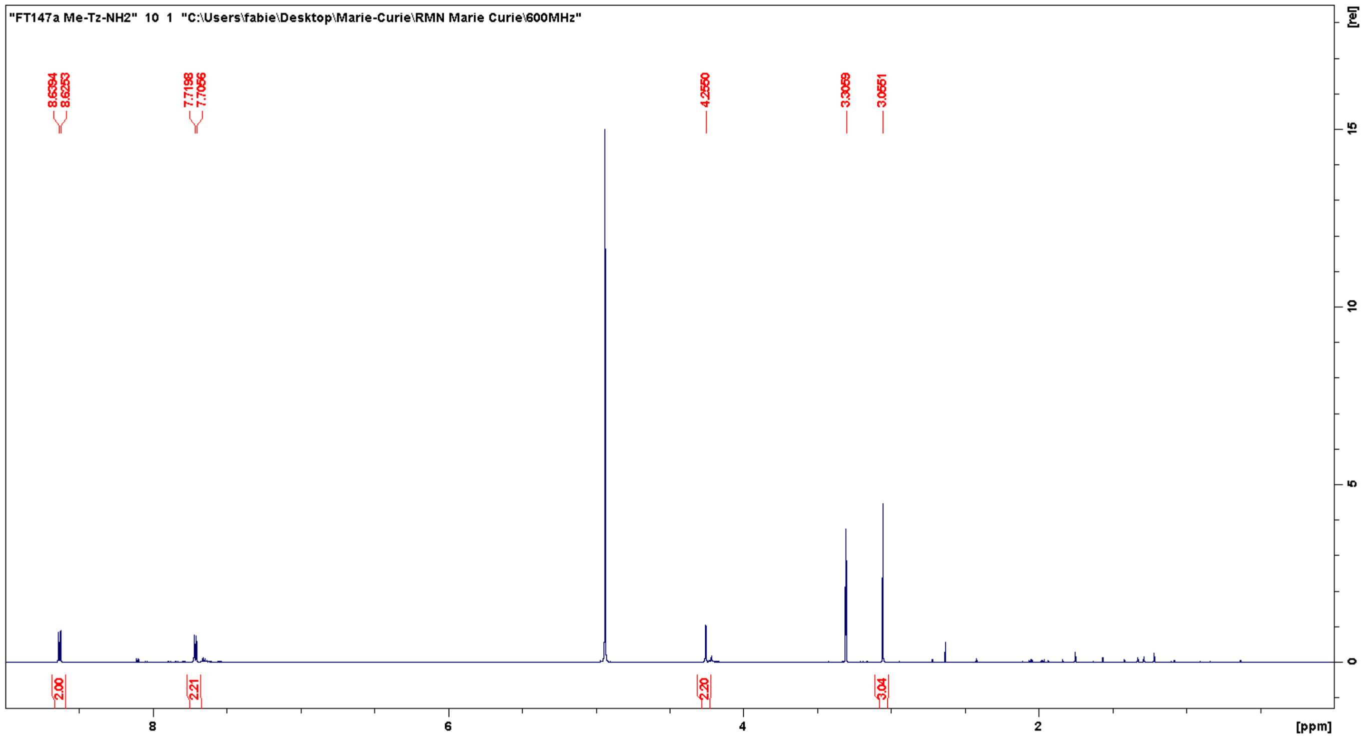

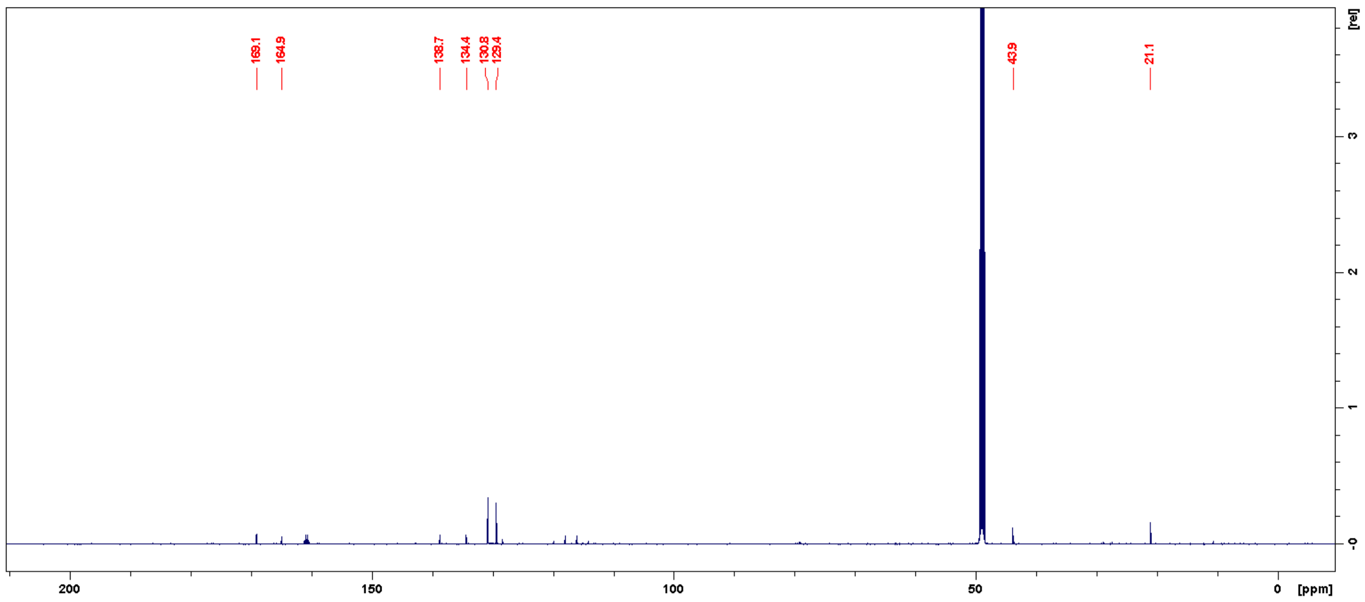

<u>Compound 10</u> - 5-((4-(6-methyl-1,2,4,5-tetrazin-3-yl)benzyl)amino)-5-oxopentanoic acid[2]

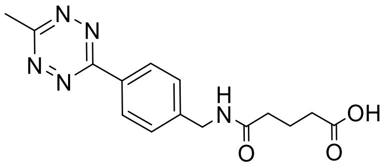

To [4-(6-methyl-1,2,4,5-tetrazin-3-yl)phenyl]methanamine **9** (480 mg, 1.52 mmol, 1 equiv.) in 100 mL of THF, was added glutaric anhydride (346 mg, 3.04 mmol, 2 equiv.) and the resultant mixture was heated to 70 °C for 4 h. The mixture was then cooled to 55 °C and stirred at this temperature for a further 12 h. The solvent was evaporated under vacuum, to give the crude material as a pink oil. The crude was solubilised in EtOAc (80 mL) and washed with 2 x NH_4Cl (aq.) (30 mL) and 2 x LiCl (aq.). The solvent was evaporated under vacuum and the product was purified by flash chromatography (0 to 8 % of MeOH in CH_2Cl_2) to give acid 5-((4-(6-methyl-1,2,4,5-tetrazin-3-yl)benzyl)amino)-5-oxopentanoic acid **10** as a red solid (150 mg, 476µmol, 32 %): ^{1}H NMR (600 MHz, $CD_3OD/CDCl_3$) δ 8.48 (d, *J* = 8.3 Hz, 2H), 7.47 (d, *J* = 8.3 Hz, 2H), 4.47 (s, 2H), 3.06 (s, 3H), 2.35-2.29 (m, 4H), 1.94 (q, *J* = 7.4 Hz, 2H) ; ^{13}C NMR (150 MHz, $CD_3OD/CDCl_3$) δ 177.3 (C), 175.2 (C), 168.6 (C), 165.3 (C), 144.9 (C), 131.9 (C), 129.7 (CH), 129.5 (CH), 44.3 (CH_2), 36.5 (CH_2), 34.5 (CH_2), 22.4 (CH_2), 22.2 (CH_3); Rf = 0.3 (5% MeOH in CH_2Cl_2).

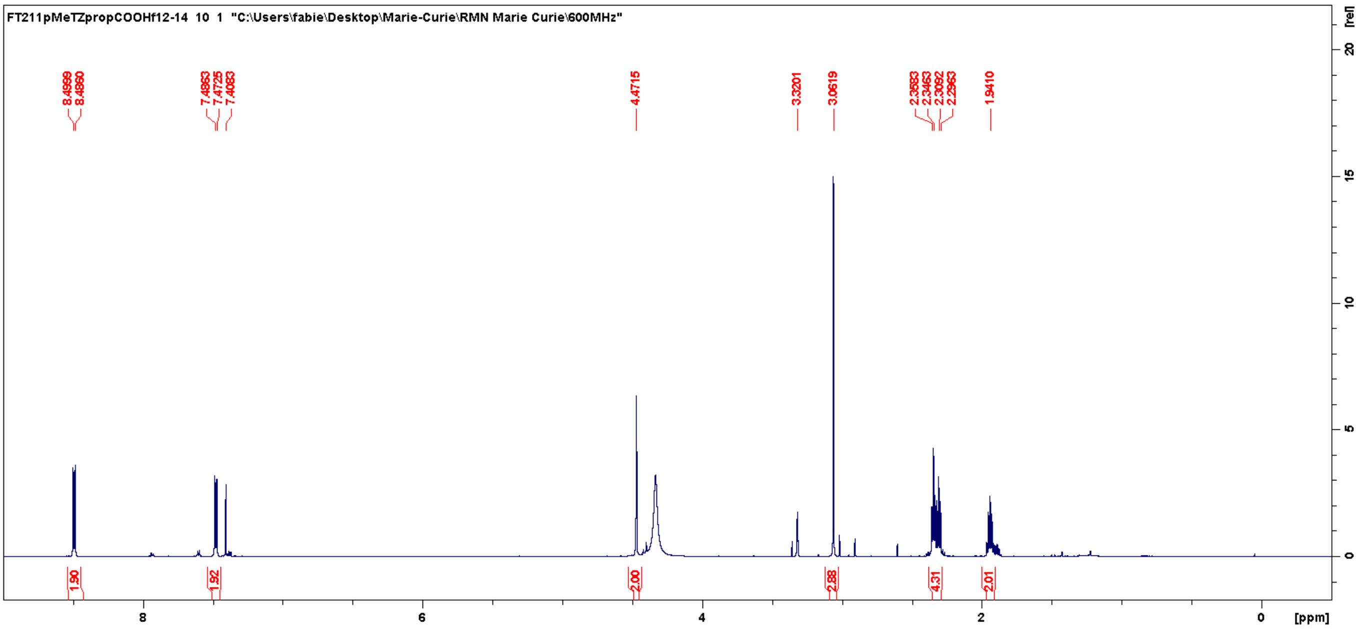
FT211pMeTZpropCOOHf12-14 10 1 "C:\Users\fabie\Desktop\Marie-Curie\RMN Marie Curie\600MHz"
8.4999
8.4860
7.4863
7.4725
7.4083
4.4715
3.3201
3.0619
2.3583
2.3453
2.3092
2.2963
1.9410
1.90
1.92
2.00
2.88
4.31
2.01
[ppm]
[rel]

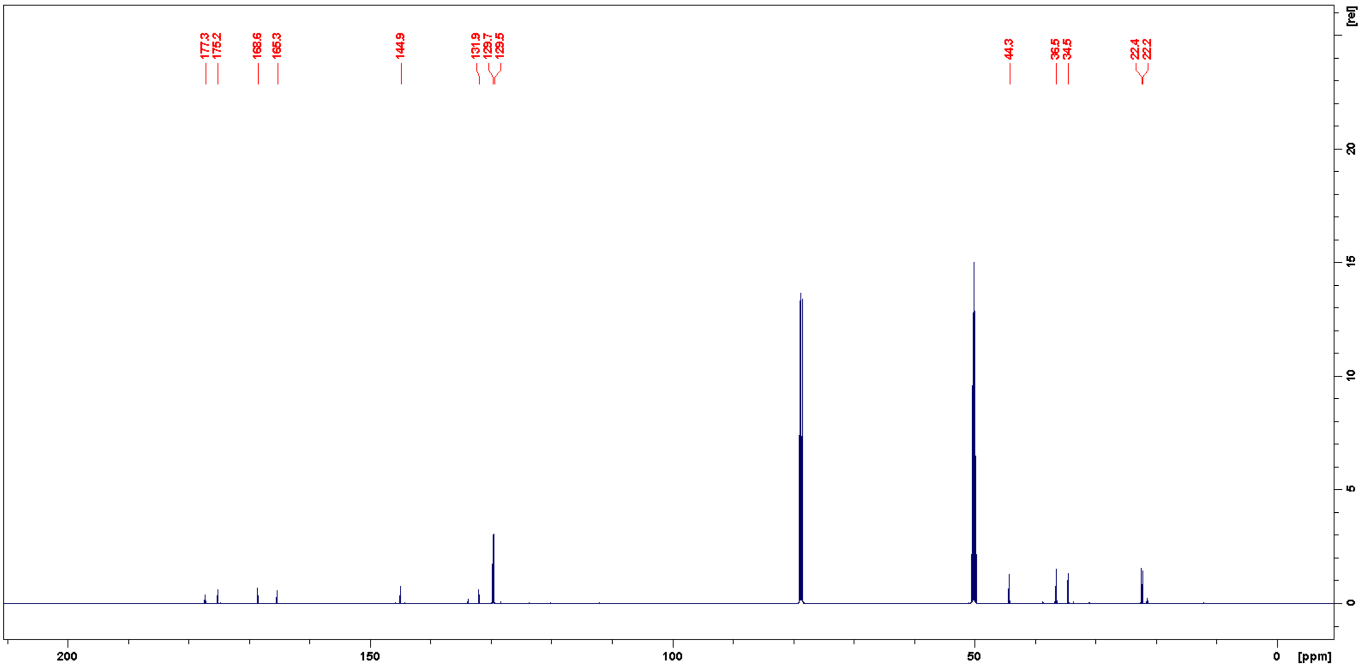
177.3
175.2
168.6
165.3
144.9
131.9
129.7
129.5
44.3
36.5
34.5
22.4
22.2
[ppm]
[rel]

Compound 11 - N1-(2-(2-(2-(2-azidoethoxy)ethoxy)ethoxy)ethyl)-N5-(4-(6-methyl-1,2,4,5-tetrazin-3-yl)benzyl)glutaramide

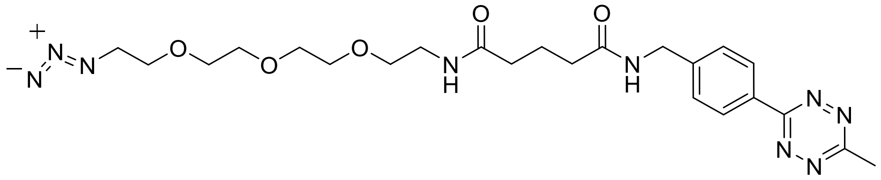

To tetrazine acid **10** (100 mg, 317 µmol, 1 equiv.) in DMF was added DIPEA (102 µL, 640 µmol, 2 equiv.) and HATU (160 mg, 416 µmol, 1.3 equiv.). The reaction mixture was stirred 30 min at room temperature. After this time, NH_2-PEG_2-N_3 (128 µL, 640 µmol, 2 equiv.) was added and the resulting mixture was stirred overnight at room temperature. The solvent was evaporated under vacuum with toluene co-evaporation (as an azeotrope). Crude is solubilised in EtOAc, washed with 1 x $NaHCO_3$ (aq.), 2 x NH_4Cl (aq.) and dried Na_2SO_4 and concentrated under vacuum evaporation. The product was purified by flash chromatography (0 to 10 % of MeOH in CH_2Cl_2), yielding N1-(2-(2-(2-(2-azidoethoxy)ethoxy)ethoxy)ethyl)-N5-(4-(6-methyl-1,2,4,5-tetrazin-3-yl)benzyl)glutaramide **11** as a pink oil (130 mg, 252 µmol, 77 %): ^1H NMR (600 MHz, $CDCl_3$) δ 8.52 (d, J = 8.4 Hz, 2H), 7.49 (d, J = 8.4 Hz, 2H), 6.74 (bs, 1H), 6.25 (bs, 1H), 4.53 (d, J = 6 Hz, 2H), 3.65 – 3.59 (m, 10H), 3.52 (t, J = 5 Hz, 2H), 3.42 – 3.39 (m, 2H), 3.36 (t, J = 5 Hz, 2H), 3.07 (s, 3H), 2.33 (t, J = 7.1 Hz, 2H), 2.25 (t, J = 7.0 Hz, 2H), 1.99 (q, J = 7.0 Hz, 2H) ; ^{13}C NMR (150 MHz, $CDCl_3$) δ 172.9 (C), 172.8 (C), 167.4 (C), 164.0 (C), 143.7 (C), 131.0 (C), 128.6 (CH), 128.3 (CH), 70.8 (CH_2), 70.7 (CH_2), 70.6 (CH_2), 70.3 (CH_2), 70.1 (CH_2), 69.8 (CH_2), 50.8 (CH_2-N_3), 43.3 (CH_2), 39.3 (CH_2), 35.4 (CH_2), 35.3 (CH_2), 22.1 (CH_2), 21.3 (CH_3) ; HRMS (ESI) calcd for $[C_{23}H_{33}N_9O_5-H]^-$: 514.2532, observed: 514.2531; Rf = 0.8 (4% MeOH in CH_2Cl_2).

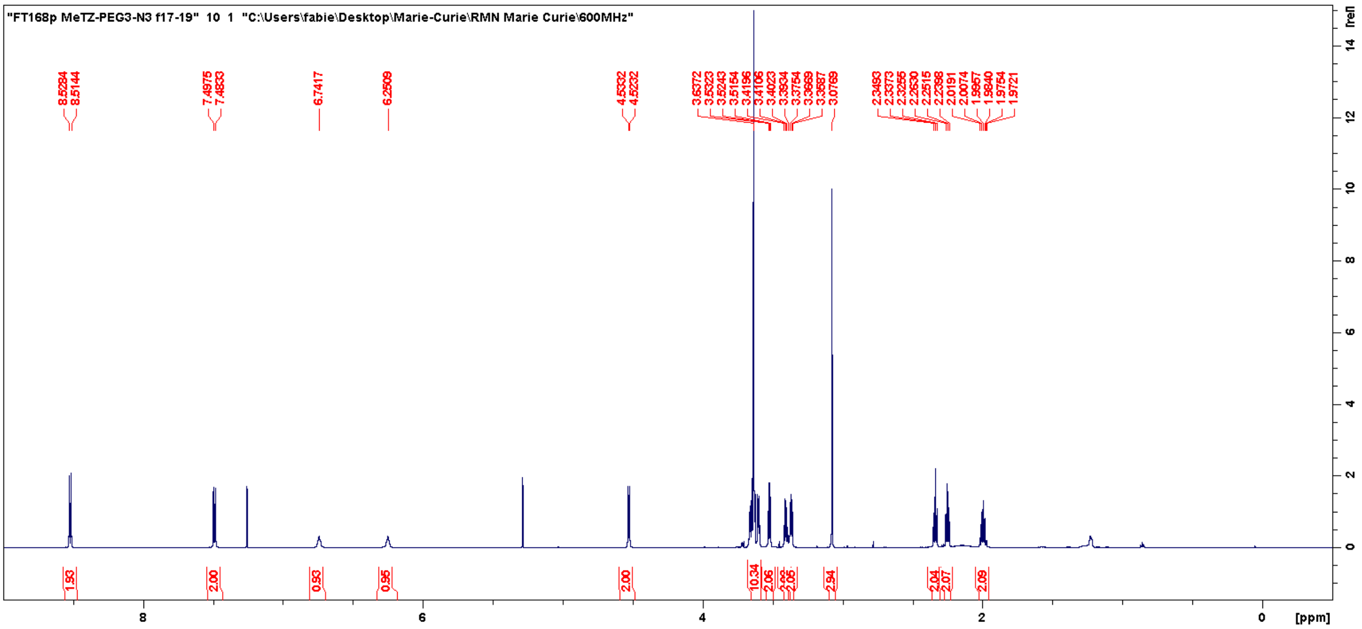

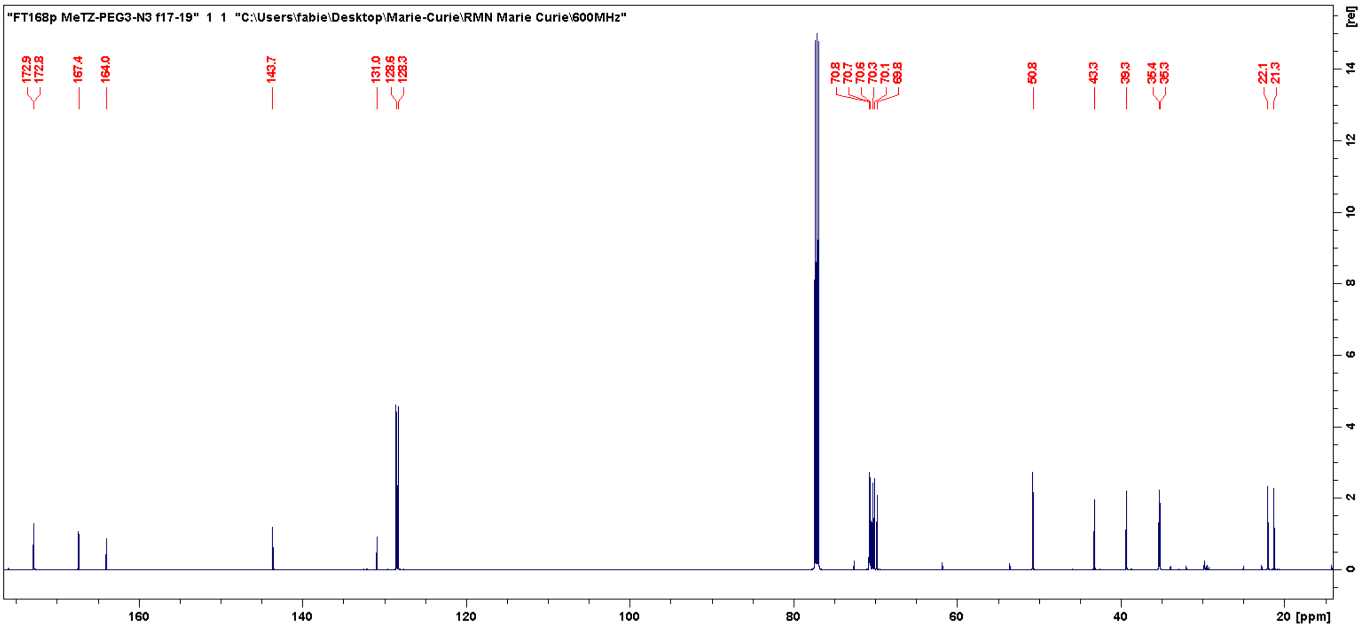

Compound 12 – tert-butyl (2-(2-(2-aminoethoxy)ethoxy)ethyl)carbamate adapted from Lee *et al.* [4]

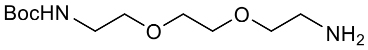

To a stirring solution of NH_2-PEG_2-NH_2 (8.15 g, 55.0 mmol, 4 equiv.) in 1,4-dioxane (60 mL) at 0 °C was added dropwise Boc_2O (3.0 g, 13.75 mmol, 1 equiv.) predisolved in 1,4-dioxane (35 mL) over 2 h, ensuring temperature did not exceed 21 °C. After this time, reaction mixture was stirred at 21 °C for a further 30 min. Following this, the reaction mixture was concentrated under vacuum, the crude residue dissolved in water (75 mL), and the organics extracted into EtOAc (5 × 30 mL). The organics were combined, dried ($MgSO_4$) and concentrated under vacuum to afford tert-butyl (3-(2-(2-(3-aminopropoxy)ethoxy)ethoxy)propyl)carbamate **12** (3.0 g, 12.04 mmol, 88 %) as a colourless oil: ^{1}H NMR (600 MHz, $CDCl_3$) δ 5.19 (br s, 1H), 3.60 – 3.57 (m, 4H), 4.93 (s, 4H), 3.52-3.47 (m, 4H), 3.29-3.28 (m, 2H), 2.85 (t, *J* = 4.9 Hz, 2H), 1.43 (s, 9H); ^{13}C NMR (150 MHz, $CDCl_3$) δ 156.2 (C), 79.3 (C), 73.5 (CH_2), 70.4 (CH_2), 70.3 (CH_2), 70.2 (CH_2), 41.8 (CH_2), 40.4 (CH_2), 28.5 (CH_3); IR (thin film) 3341, 2971, 2925, 2866, 1702, 1517, 1170, 1117 cm^{-1}; HRMS (ESI) calcd for $[C_{11}H_{24}N_2O_4+H]^+$: 249.1809, observed: 249.1809.

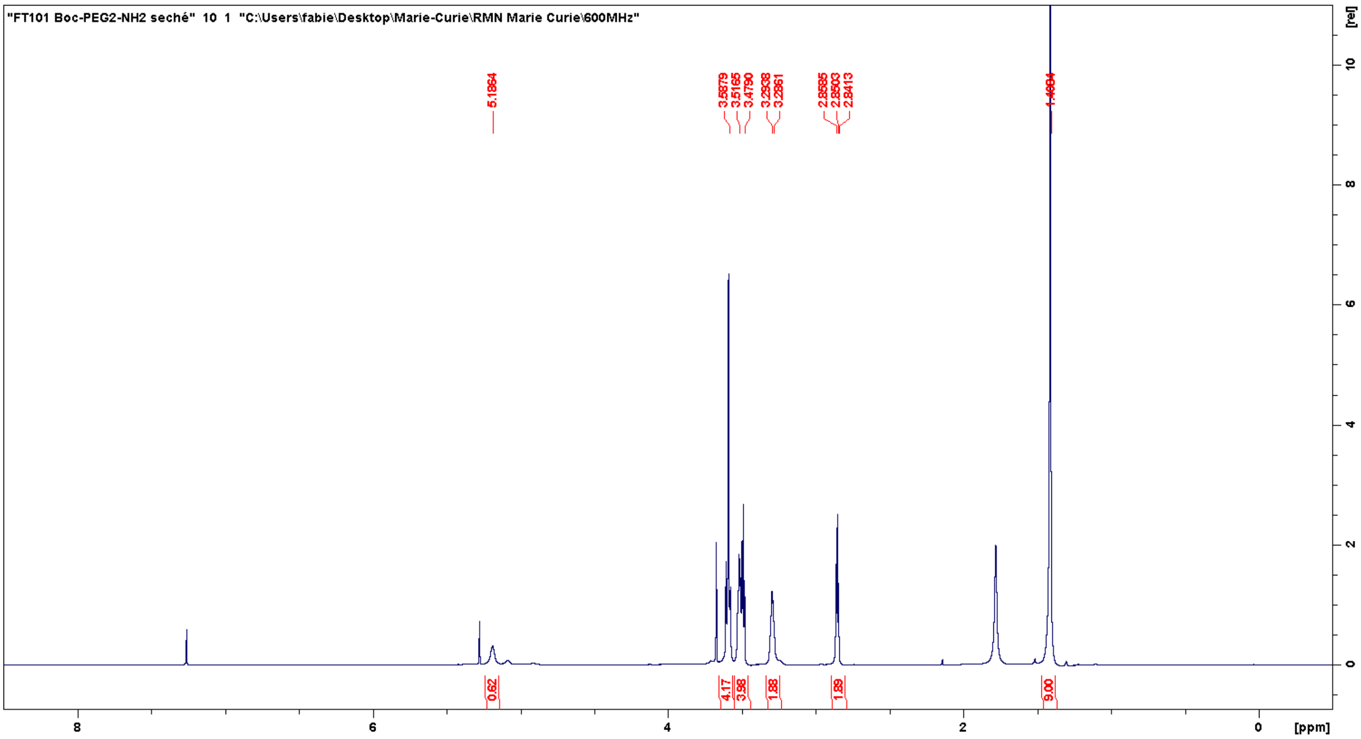

"FT101 Boc-PEG2-NH2 seché" 10 1 "C:\Users\fabie\Desktop\Marie-Curie\RMN Marie Curie\600MHz"
5.1864
3.5879
3.5165
3.4790
3.2938
3.2861
2.8585
2.8503
2.8413
1.4634
0.62
4.17
3.58
1.88
1.89
9.00
[rel]
[ppm]

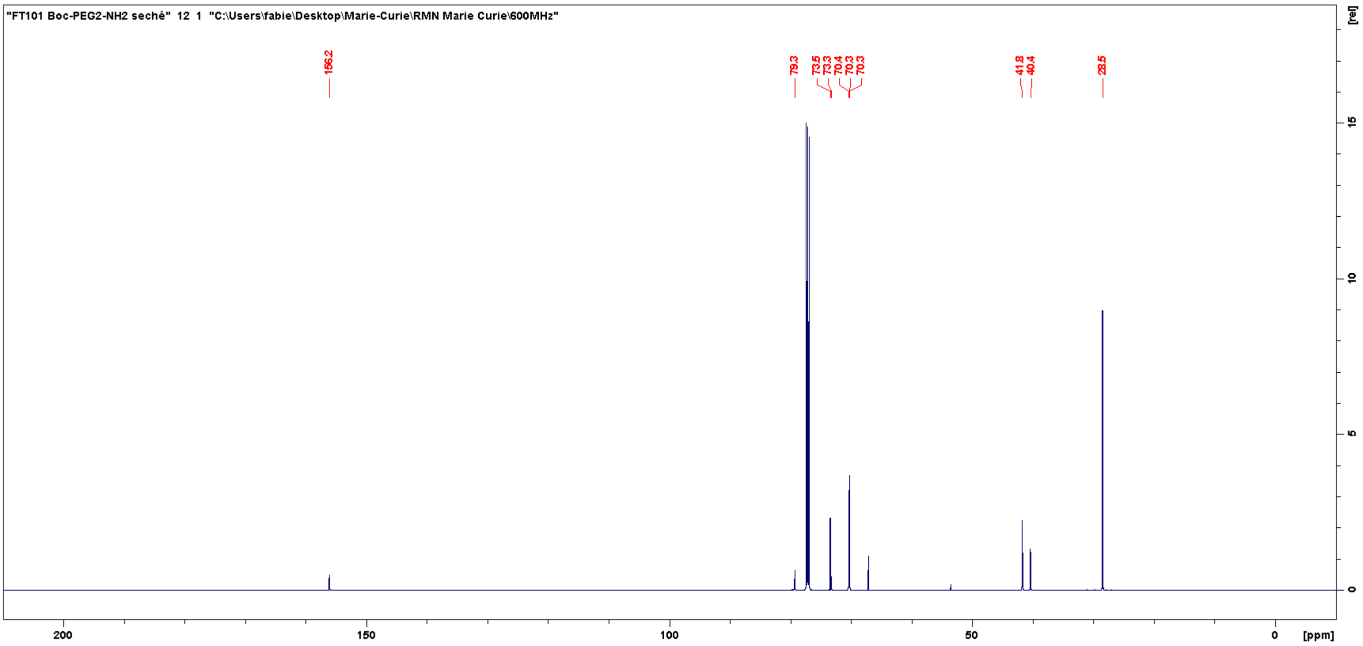

"FT101 Boc-PEG2-NH2 seché" 12 1 "C:\Users\fabie\Desktop\Marie-Curie\RMN Marie Curie\600MHz"
156.2
79.3
73.5
73.3
70.4
70.3
70.3
41.8
40.4
28.5
[rel]
[ppm]

<u>Compound 13</u> – di-tert-butyl (10,12-dioxo-11-(prop-2-yn-1-yl)-3,6,16,19-tetraoxa-9,13-diazahenicosane-1,21-diyl)dicarbamate adapted from Chui *et al.* [5]

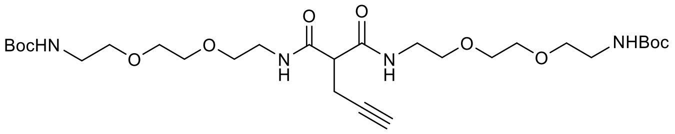

In a 250 mL flask equipped with a reflux condenser were combined (neat) tert-butyl (3-(2-(2-(3-aminopropoxy)ethoxy)ethoxy)propyl)carbamate **12** (512 mg, 460 µL, 2.91 mmol, 1 equiv.) and dimethylpropargylmalonate (1.73 g, 6.98 mmol, 2.4 equiv.). The viscous mixture was heated under reflux (80 °C) overnight. Crude was then purified by flash chromatography (15 to 100 % EtOAc in cyclohexane then 0 to 15 % MeOH in EtOAc) to afford di-tert-butyl (10,12-dioxo-11-(prop-2-yn-1-yl)-3,6,16,19-tetraoxa-9,13-diazahenicosane-1,21-diyl)dicarbamate **13** (397 mg, 659 µmol, 23 %) as an orange oil: ^{1}H NMR (600 MHz, $CDCl_3$) δ 7.21 (br s, 1H), 5.25 (br s, 1H), 3.60 – 3.56 (m, 8H), 3.56 – 3.52 (m, 8H), 3.49 – 3.43 (m, 4H), 3.34 – 3.24 (m, 5H), 2.75 (dd, *J* = 2.2 Hz, *J* = 7.4 Hz, 2H), 2.08 (m, 1H), 1.43 (s, 18H); ^{13}C NMR (150 MHz, $CDCl_3$) δ 169.1 (C), 156.2 (C), 80.5 (C), 79.4 (C), 71.3 (CH), 70.4 (CH_2), 70.3 (CH_2), 53.0 (CH), 40.5 (CH_2), 39.6 (CH_2), 28.6 (CH_3), 21.1 (CH_2); Rf = 0.3 in EtOAc/MeOH (9/1); IR (thin film) 3315, 2973, 2930, 2869, 1674, 1525, 1170 cm^{-1}; HRMS (ESI) calcd for $[C_{28}H_{50}N_4O_{10}+H]^+$: 603.35997, observed: 603.3597.

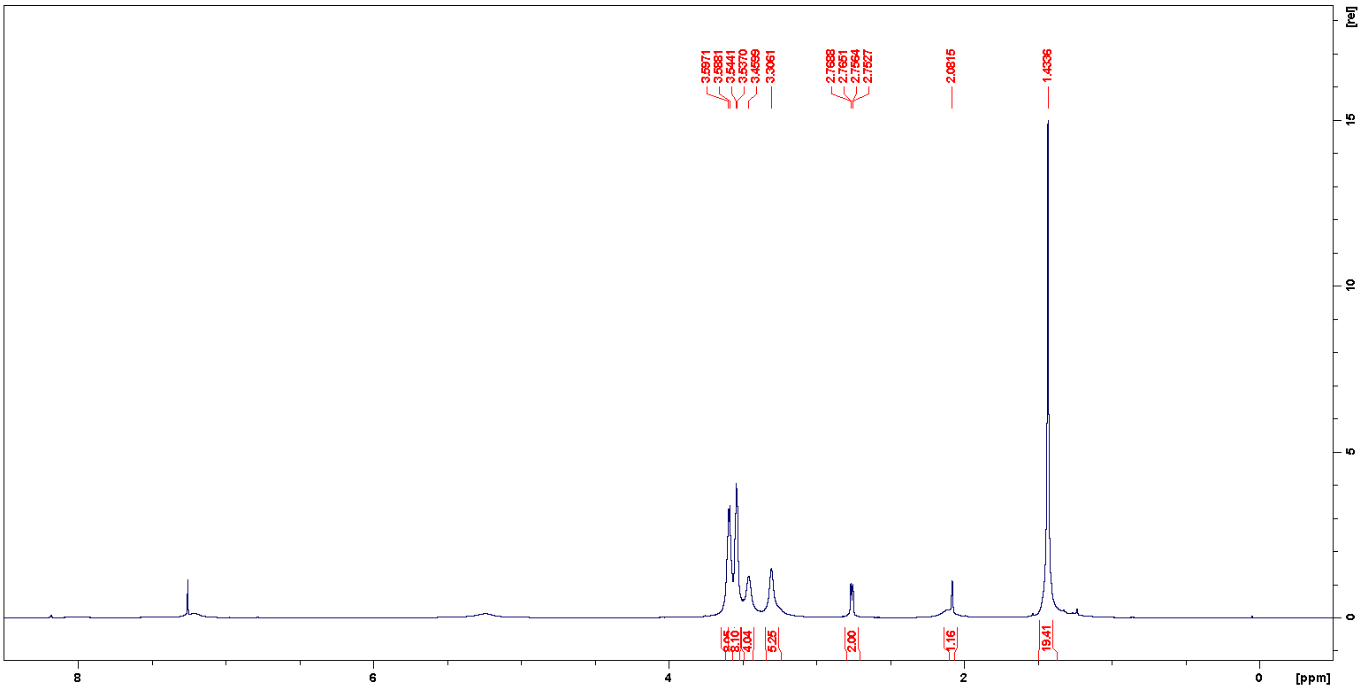

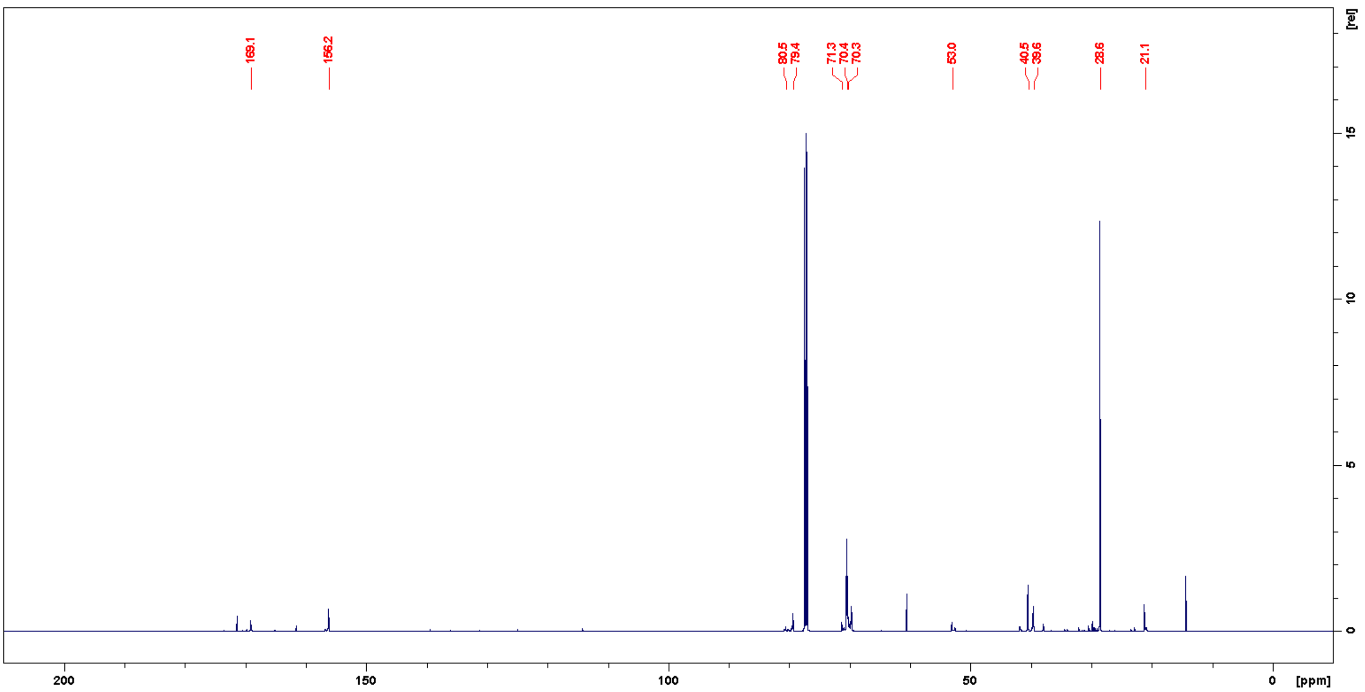

Compound 14 - di-tert-butyl (11-((1-(1-(4-(6-methyl-1,2,4,5-tetrazin-3-yl)phenyl)-3,7-dioxo-11,14,17-trioxa-2,8-diazanonadecan-19-yl)-1H-1,2,3-triazol-4-yl)methyl)-10,12-dioxo-3,6,16,19-tetraoxa-9,13-diazahenicosane-1,21-diyl)dicarbamate

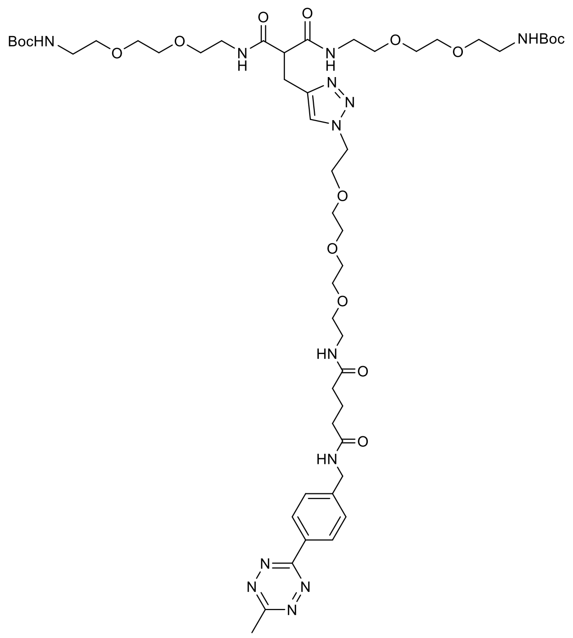

To di-tert-butyl (10,12-dioxo-11-(prop-2-yn-1-yl)-3,6,16,19-tetraoxa-9,13-diazahenicosane-1,21-diyl)dicarbamate **13** (45.0 mg, 75 µmol, 1 equiv.) in DMF (12 mL) was added a solution of $CuSO_4$ (3.0 mg, 19 µmol, 0.25 equiv.) and THPTA (24.5 mg, 56 µmol, 0.75 equiv.) in PBS (pH 7.4, 9 mL) and tetrazine-azide **11** (40 mg, 75 µmol, 1 equiv.) under argon. A solution of sodium ascorbate (18.4 mg, 94 µmol, 1.25 equiv.) in H_2O (3 mL) was added under argon and the resulting mixture is stirred at 37 °C under argon for 1 h. After this time, crude is diluted in DCM (50 mL), washed with NH_4Cl sat. (3 x 20 mL), dried over $MgSO_4$, and purified by flash chromatography (0 to 10 % MeOH in DCM) to afford di-tert-butyl (11-((1-(1-(4-(6-methyl-1,2,4,5-tetrazin-3-yl)phenyl)-3,7-dioxo-11,14,17-trioxa-2,8-

diazanonadecan-19-yl)-1H-1,2,3-triazol-4-yl)methyl)-10,12-dioxo-3,6,16,19-tetraoxa-9,13-diazahenicosane-1,21-diyl)dicarbamate **14** (19 mg, 17 µmol, 23 %) as a pink oil: ^{1}H NMR (600 MHz, $CDCl_3$) δ 8.5 (d, *J* = 8.3 Hz, 2H), 7.54 (br s, 1H), 7.50 (d, *J* = 8.3 Hz, 2H), 4.54 – 4.52 (m, 2H), 4.47 (t, *J* = 4.9, 2H), 3.83 (t, *J* = 4.9, 2H), 3.65-3.64 (m, 2H), 3.63 – 3.21 (m, 37H), 3.08 (s, 3H), 2.37 – 2.33 (m, 2H), 2.29 – 2.25 (m, 2H), 2.02 – 2.00 (m, 2H), 1.41 (s, 18H); ^{13}C NMR (150 MHz, $CDCl_3$) δ 173.3 (C), 172.9 (C), 172.8 (C), 167.4 (C), 164.0 (C), 156.3 (C), 144.3 (CH), 143.8 (C), 130.9 (C), 128.6 (CH), 128.3 (CH), 123.5 (CH), 79.4 (C), 70.8 – 69.7 (13 x CH_2), 69.5 (CH_2), 50.8 (CH), 50.3 (CH_2), 43.3 (CH_2), 43.2 (CH_2), 40.5 (CH_2), 39.5 (CH_2), 39.3 (CH_2), 35.5 (CH_2), 35.3 (CH_2), 28.6 (CH_3), 27.2 (CH_2), 22.2 (CH_2), 21.3 (CH_3); Rf = 0.45 in DCM/MeOH (9/1); IR (thin film) 3313, 2917, 2870, 1651, 1552, 1170 cm^{-1}; HRMS (ESI) calcd for $[C_{51}H_{83}N_{13}O_{15}+H]^+$: 1118.6204, observed: 1118.6211.

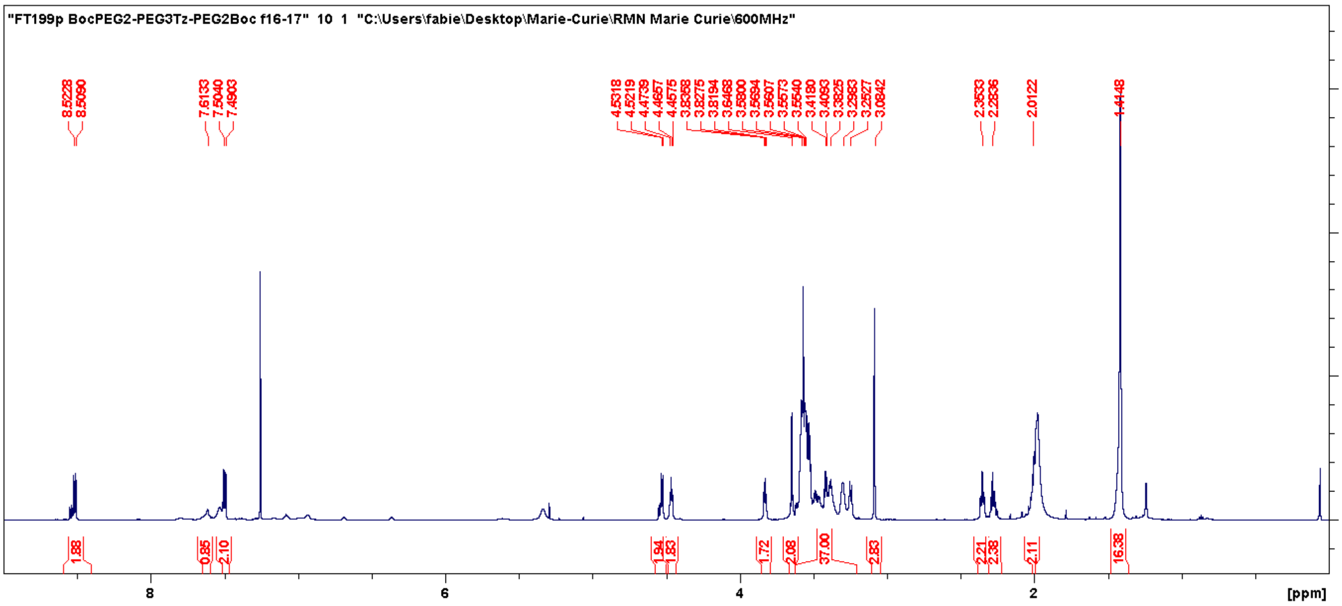

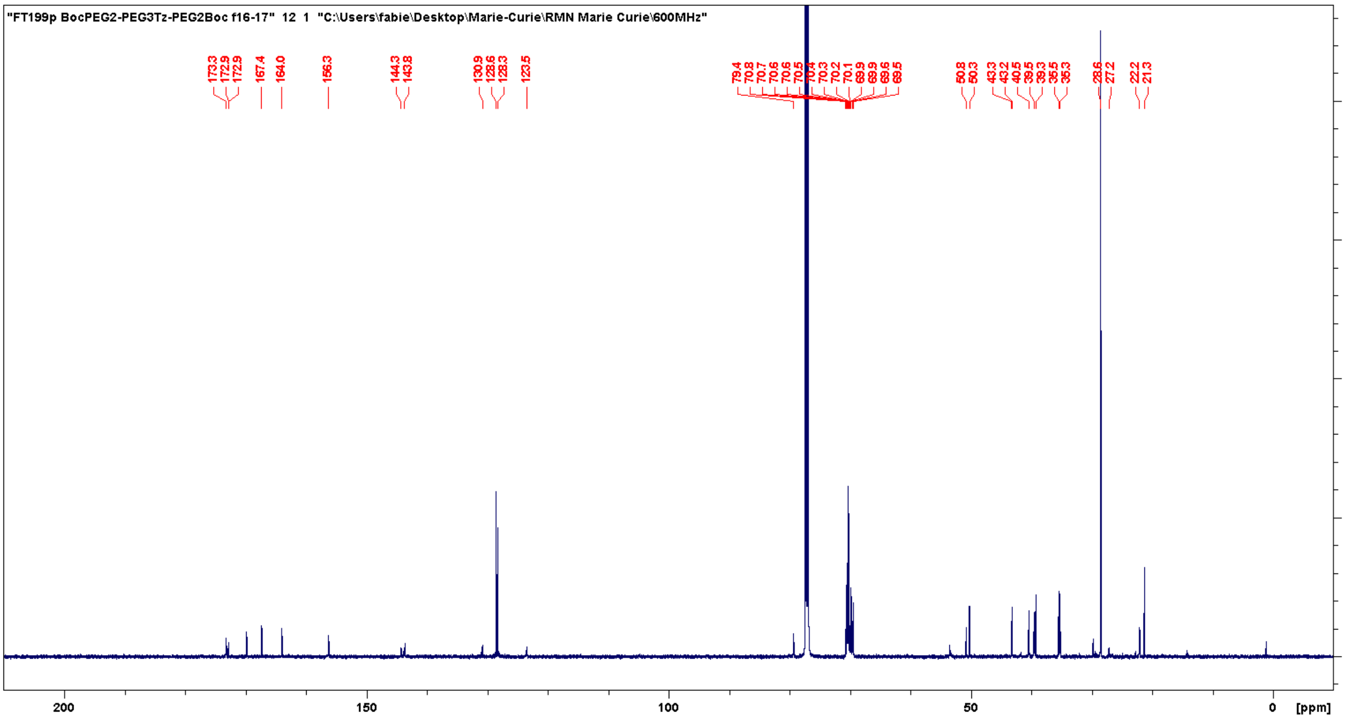

<u>Compound 15</u> - N1-(2-(2-(2-(2-(4-(3-((2-(2-(2-aminoethoxy)ethoxy)ethyl)amino)-2-((2-(2-(2-aminoethoxy) ethoxy)ethyl)carbamoyl)-3-oxopropyl)-1H-1,2,3-triazol-1-yl)ethoxy)ethoxy)ethoxy)ethyl)-N5-(4-(6-methyl-1,2,4,5-tetrazin-3-yl)benzyl)glutaramide

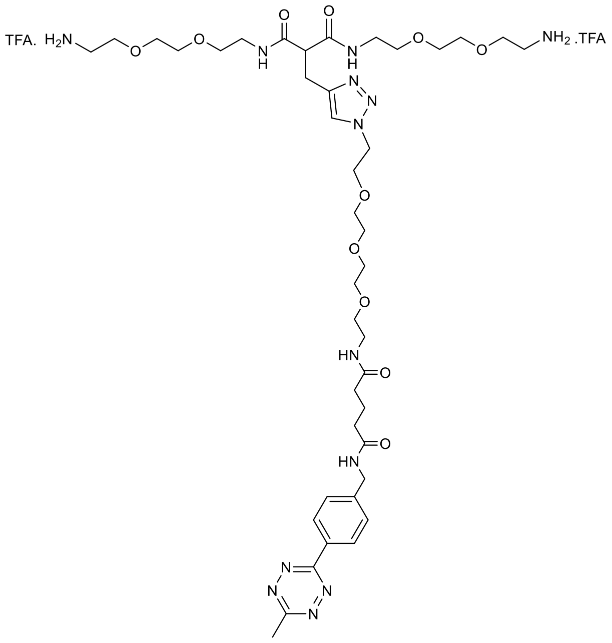

Tetrazine **14** (19 mg, 17 µmol, 1 equiv.) was stirred in a (1/1) DCM/TFA mixture (50 mL) at room temperature for 30 min. After this time, solvent were removed under vacuum to afford N1-(2-(2-(2-(2-(4-(3-((2-(2-(2-aminoethoxy)ethoxy)ethyl)amino)-2-((2-(2-(2-aminoethoxy)ethoxy)ethyl)carbamoyl)-3-oxopropyl)-1H-1,2,3-triazol-1-yl)ethoxy)ethoxy)ethoxy)ethyl)-N5-(4-(6-methyl-1,2,4,5-tetrazin-3-yl)benzyl)glutaramide **15** (22.8 mg, quant.) as a pink viscous TFA salt: ^{1}H NMR (600 MHz, $CD_3OD/CDCl_3$) δ 8.48 (d, *J* = 8.3 Hz, 2H), 7.69 (s, 1H), 7.48 (d, *J* = 8.3 Hz, 2H), 4.48 (t, *J* = 5.0 Hz, 2H), 4.46 (br s, 2H), 3.83 (t, *J* = 5.0, 2H), 3.68 (t, = 5.0 Hz, 4H), 3.64 – 3.43 (m, 25H), 3.36 – 3.33 (m, 6H), 3.17 (d, *J* = 7.6 Hz, 2H), 3.12 – 3.10 (m, 4H), 3.05 (s, 3H), 2.28 (t, *J* = 7.4 Hz, 2H), 2.23 (t, *J* = 7.4 Hz, 2H), 1.92 (q, *J* = 7.4 Hz, 2H); ^{13}C NMR (150 MHz, $CDCl_3$) δ 175.4 (C), 171.3 (C), 168.6 (C), 165.3 (C), 145.3 (C), 145.0 (C), 132.0 (C), 129.6 (CH), 129.4 (CH), 125.0 (CH), 71.7 (CH_2), 71.6 (CH_2), 71.5 (CH_2), 71.4 (CH_2), 71.3 (CH_2), 70.8 (CH_2), 70.7 (CH_2), 70.5 (CH_2), 54.5 (CH), 51.5 (CH_2), 44.2 (CH_2), 40.8 (CH_2), 40.6 (CH_2), 40.5 (CH_2), 36.5 (CH_2), 27.4 (CH_2), 22.2 (CH_2), 21.3 (CH_3); IR (thin film) 3291, 2916, 1676, 1552, 1130 cm^{-1}; HRMS (ESI) calcd for $[C_{41}H_{67}N_{13}O_{11}+H]^+$: 918.5156, observed: 918.5134.

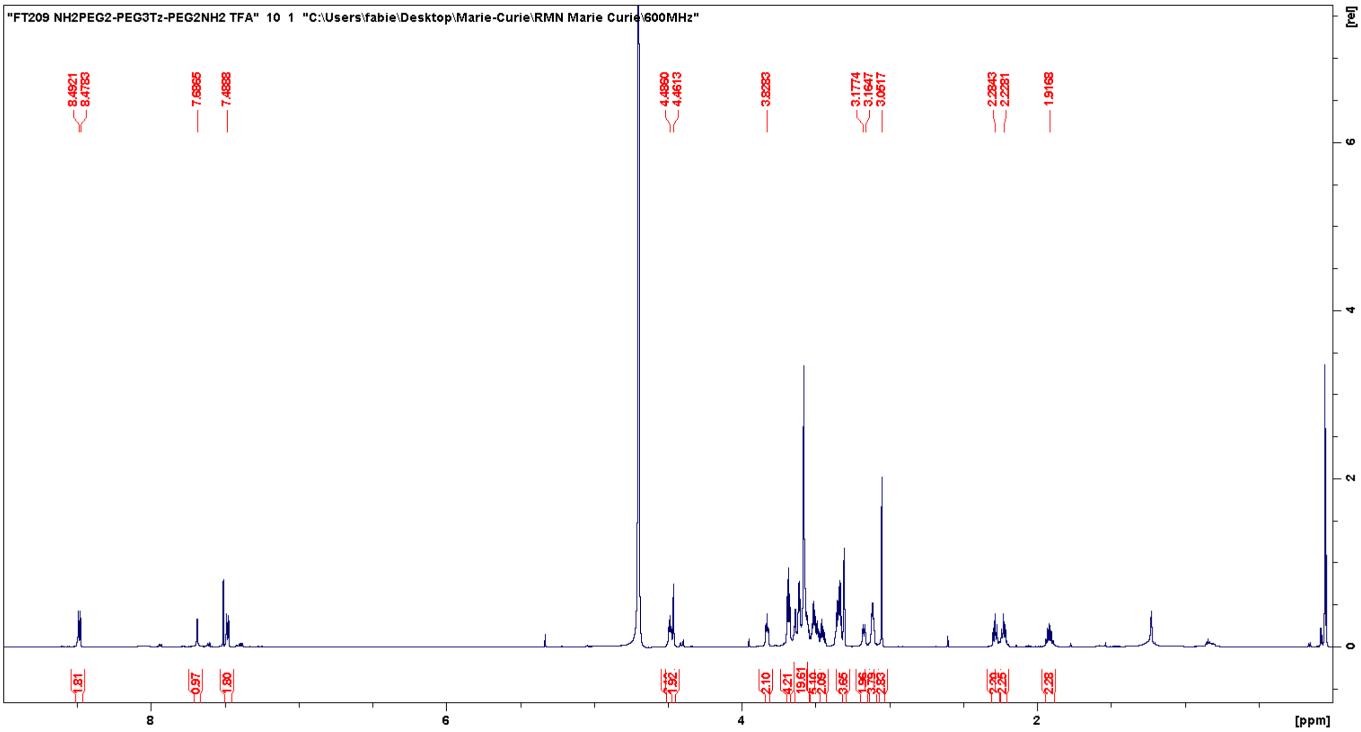
"FT209 NH2PEG2-PEG3Tz-PEG2NH2 TFA" 10 1 "C:\Users\fabie\Desktop\Marie-Curie\RMN Marie Curie\600MHz"
8.4921
8.4783
7.6865
7.4888
4.4860
4.4613
3.8263
3.1774
3.1647
3.0517
2.2843
2.2281
1.9168
1.81
0.97
1.80
2.12
1.92
2.10
4.21
19.61
5.10
2.09
3.65
1.96
1.79
2.83
2.20
2.25
2.28
[ppm]
[rel]

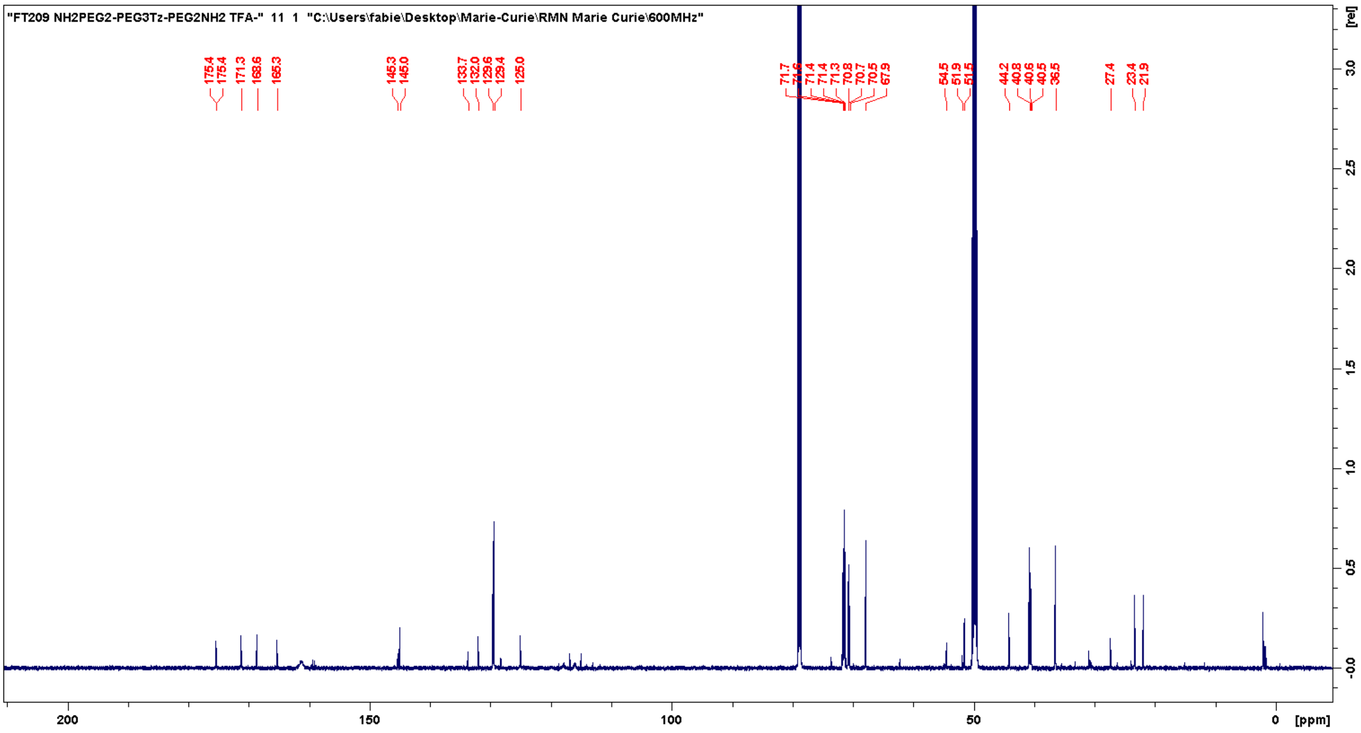
"FT209 NH2PEG2-PEG3Tz-PEG2NH2 TFA-" 11 1 "C:\Users\fabie\Desktop\Marie-Curie\RMN Marie Curie\600MHz"
175.4
175.4
171.3
168.6
165.3
145.3
145.0
133.7
132.0
129.6
129.4
125.0
71.7
71.6
71.4
71.3
70.8
70.7
70.5
67.9
54.5
51.9
51.5
44.2
40.8
40.6
40.5
36.5
27.4
23.4
21.9
[ppm]
[rel]

Compound 16 - N1-(2-(2-(2-(2-(4-(16-(2-(4-azidobenzyl)-4,5-dibromo-3,6-dioxo-3,6-dihydropyridazin-1(2H)-yl)-2-((2-(2-(2-(3-(2-(4-azidobenzyl)-4,5-dibromo-3,6-dioxo-3,6-dihydropyridazin-1(2H)-yl)propanamido)ethoxy) ethoxy)ethyl)carbamoyl)-3,14-dioxo-7,10-dioxa-4,13-diazahexadecyl)-1H-1,2,3-triazol-1-yl)ethoxy)ethoxy)ethoxy) ethyl)-N5-(4-(6-methyl-1,2,4,5-tetrazin-3-yl)benzyl)glutaramide

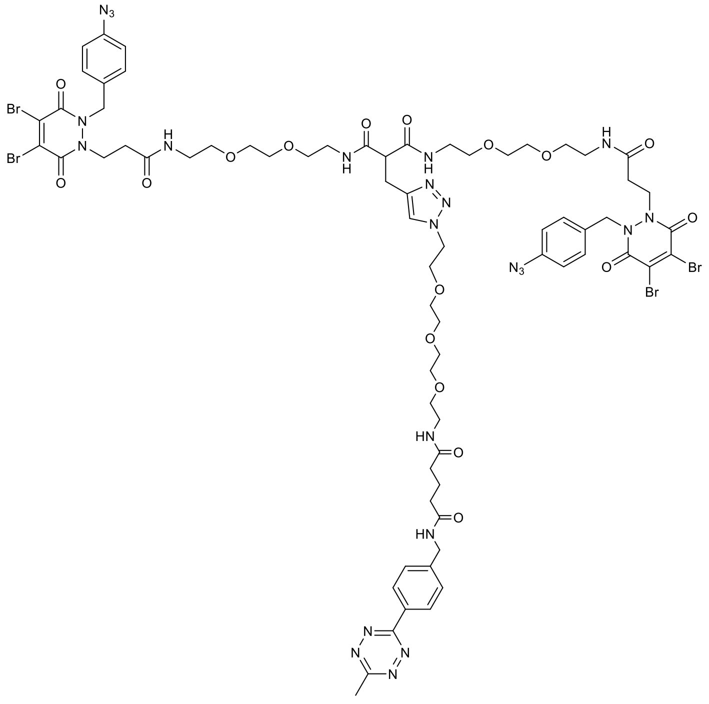

To a solution of tetrazine salt **15** (9.7 mg, 8.5 µmol, 1 equiv.) in DMF (200 µL) was added a solution of diBrPD-NHS **6** (11.6 mg, 20.4 µmol, 2.4 equiv.) in DMF (200 µL) and DIEA (6 µL, 34.0 µmol, 4 equiv.). The mixture was stirred for 12 h at room temperature. After this time, crude was concentrated under vacuum, solubilised in DCM (30 mL), washed with NH_4Cl sat. (3 x 10 mL) and dried with Na_2SO_4. Solvent was removed under vacuum and crude was purified by flash chromatography (0 to 20 % of MeOH in DCM) to afford N1-(2-(2-(2-(2-(4-(16-(2-(4-azidobenzyl)-4,5-dibromo-3,6-dioxo-3,6-dihydropyridazin-1(2H)-yl)-2-((2-(2-(2-(3-(2-(4-azidobenzyl)-4,5-dibromo-3,6-dioxo-3,6-dihydropyridazin-1(2H)-yl)propanamido)ethoxy)ethoxy)ethyl)carbamoyl)-3,14-dioxo-7,10-dioxa-4,13-diazahexadecyl)-1H-1,2,3-triazol-1-yl)ethoxy)ethoxy)ethoxy)ethyl)-N5-(4-(6-methyl-1,2,4,5-tetrazin-3-yl)benzyl)glutaramide **16** (3.4 mg, 1.86 µmol, 22 %) as a pink oil: ^{1}H NMR (600 MHz, $CDCl_3$) δ 8.51 (d, *J* = 8.2 Hz, 2H), 7.65 – 7.63 (s, 1H), 7.49 (d, *J* = 8.2 Hz, 2H), 7.21 – 7.16 (m, 4H), 6.99 (d, *J* = 8.4 Hz, 4H), 5.46 – 5.40 (m, 4H), 4.53 – 4.52 (m, 2H), 4.49 – 4.45 (m, 2H), 4.30 – 4.16 (m, 4H), 3.84 – 3.82 (m, 2H), 3.58 – 3.33 (m, 37H), 3.21 – 3.23 (m, 2H), 3.09 (s, 3H), 2.62 – 2.49 (m, 4H), 2.36 – 2.33 (m, 2H), 2.27 (t, *J* = 6.9 Hz, 2H), 2.00 – 1.98 (m, 2H); ^{13}C NMR (150 MHz, $CDCl_3$) δ 173.2 (C), 170.0 (C), 169.9 (C), 169.7 (C), 169.1 (C), 169.0 (C), 144.1 (C), 140.4 (C), 136.1 (C), 129.0 (CH), 128.6 (CH), 128.3 (CH), 123.5 (CH), 119.9 (CH), 54.1 (CH), 50.3 (CH_2), 49.9 (CH_2), 48.3 (CH_2), 44.3 (CH_2), 43.3 (CH_2), 42.5 (CH_2), 39.7 (CH_2), 39.6 (CH_2), 39.5 (CH_2), 39.3 (CH_2), 35.5 (CH_2), 35.3 (CH_2), 33.9 (CH_2), 33.8 (CH_2), 33.6 (CH_2), 29.8 (CH_2), 29.5 (CH_2), 27.5 (CH_2), 22.1 (CH_2), 21.3 (CH_3); IR (thin film) 3388, 2916, 1635, 1590, 1552 cm^{-1}; HRMS (ESI) calcd for $[C_{69}H_{85}N_{23}O_{17}Br_4+H]^+$: 1824.3300, observed: 1824.3283.

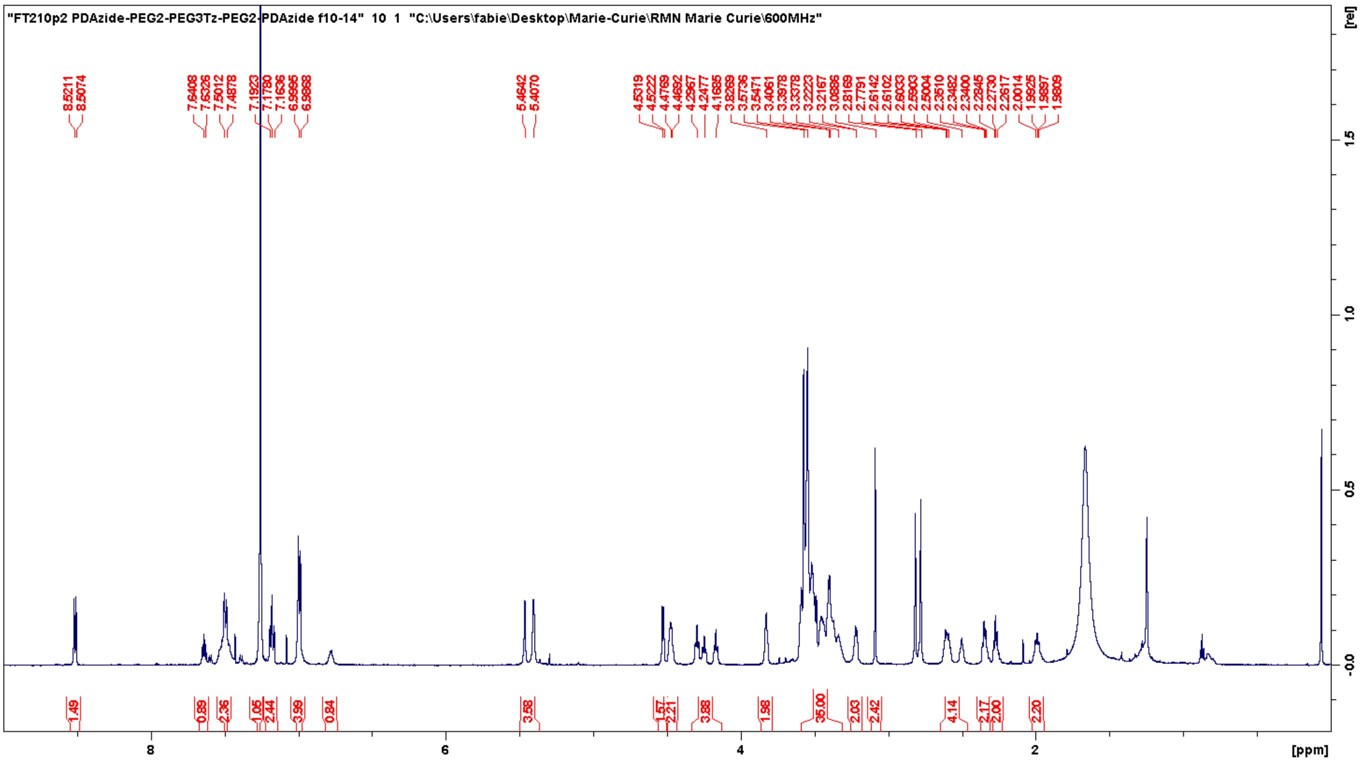
"FT210p2 PDAzide-PEG2-PEG3Tz-PEG2-PDAzide f10-14" 10 1 "C:\Users\fabie\Desktop\Marie-Curie\RMN Marie Curie\600MHz"

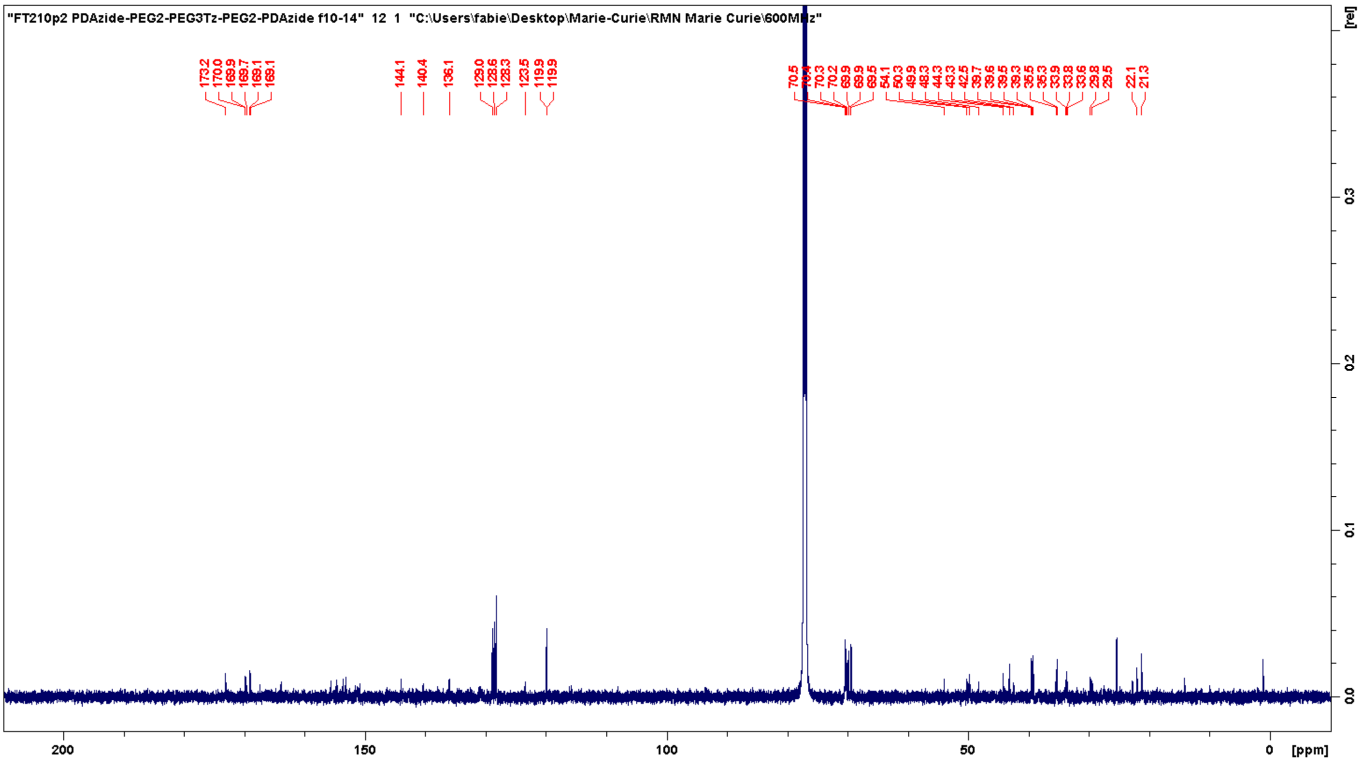
"FT210p2 PDAzide-PEG2-PEG3Tz-PEG2-PDAzide f10-14" 12 1 "C:\Users\fabie\Desktop\Marie-Curie\RMN Marie Curie\600MHz"

2. Chemical biology section

2.1 General experimental details for chemical biology

Conjugation experiments were carried out in standard 1.5 mL Eppendorf tubes. All buffer solutions were prepared with double-deionised water and filter-sterilized for long-term storage. BBS refers to borate buffered saline (25 mM borate, 25 mM NaCl, pH 8.0, 2 mM EDTA), 5 × BBS refers to borate buffered saline (125 mM borate, 125 mM NaCl, pH 8.0, 10 mM EDTA) and PBS refers to phosphate buffered saline (10 mM phosphate, 2.7 mM KCl, 137 mM NaCl, pH 7.4) unless otherwise stated. Buffer exchange was carried out using sample concentrators (Sartorius Stedim, Vivaspin, MWCO 3, 5 or 10 kDa) or desalting columns (Zeba™ Spin, ThermoFisher Scientific, 7k MWCO). Rituximab (anti-CD20) and Ontruzant/Trastuzumab (anti-HER2) antibodies were purchased from UCLH. For protein A purification Pierce™ Protein A IgG binding Buffer was used.

2.2 Protein LC-MS general protocol

Protein conjugates were prepared for analysis by desalting into distilled water (Zeba™ Spin, ThermoFisher Scientific, 7k MWCO) to achieve approximate concentrations of 4-5 µM and submitted to the UCL Chemistry Mass Spectrometry Facility at the Chemistry Department, UCL for analysis on the Agilent 6510 QTOF LC-MS system (Agilent, UK). For mAb and Fc protein samples, a deglycosylation step was realised *via* addition of PNGase F (0.1 µL of New England BioLab® PNGase F glycerol free solution per 10 µL of 5 µM protein solution) and incubation at room temperature for 12 h. 10-20 µL of each sample was injected onto a PLRP-S, 1000 Å, 8 µM, 150 mM × 2.1 mM column (Agilent, UK), which was maintained at 60 °C. The separation was achieved using mobile phase A (5 % MeCN in 0.1 % formic acid) and B (95 % MeCN, 5 % water 0.1 % formic acid) using a gradient elution (Table 1). The column effluent was continuously electrosprayed into the capillary ESI source of the Agilent 6510 QTOF mass spectrometer and ESI mass spectra were acquired in positive electrospray ionisation (ESI) mode using the m/z range 1,000−8,000 in profile mode with Quad AMU set to 500. The raw data was converted to zero charge mass spectra using a maximum entropy deconvolution algorithm, over the appropriate regions as identified via the LC trace, with the software, MassHunter (version B.07.00).

Table 1 | Gradient for LC-MS elution.

System used: Agilent 6510 QTOF LC-MS (Agilent, UK). Column: PLRP-S, 1000 Å, 8 µM, 150 mM × 2.1 mM (Agilent, UK).

Time (min)	%A (H_2O 0.1% FA)	%B (MeCN 0.1% FA)
0	85	15
2	85	15
3	68	32
4	68	32

14	50	50
18	5	95
20	5	95
22	85	15
25	85	15

2.3 SDS-PAGE gels general protocols

Non-reducing glycine-SDS-PAGE with 10 % or 12 % acrylamide gels (based on protein size: 10 % for 45-150 kDa, 12 % for 40-100 kDa) were performed following standard lab procedures. A 6% stacking gel was used and a broad-range MW marker (10–250 kDa, PageRuler™ Plus Pre-stained Protein Ladder, Thermo Scientific™) was co-run to estimate protein weights. Samples (~10 µL at ~5 µM protein) were mixed with loading buffer (1 µL, composition for 6 × SDS: 1 g SDS, 3 mL glycerol, 6 mL 0.5 M Tris buffer pH 6.8, 2 mg bromophenol blue in 10 mL) and heated at 90 °C for 5 min. 6-8 µL of the samples was loaded into each well. The gels were run at 30-35 mA for 30-50 min in 1 × SDS running buffer. The gels were stained with Coomassie Blue dye, and destained with distilled water under microwave irradiation.

Reducing glycine-SDS-PAGE with 10 % or 12 % acrylamide gels (based on protein size: 10 % for 45-150 kDa, 12 % for 40-100 kDa) were performed following standard lab procedures. A 6% stacking gel was used and a broad-range MW marker (10–250 kDa, PageRuler™ Plus Pre-stained Protein Ladder, Thermo Scientific™) was co-run to estimate protein weights. Samples (~10 µL at ~5 µM protein) were mixed with 1 µL of TCEP solution (10mM in H_2O) and loading buffer (1 µL, composition for 6 × SDS: 1 g SDS, 3 mL glycerol, 6 mL 0.5 M Tris buffer pH 6.8, 2 mg bromophenol blue in 10 mL) and heated at 90 °C for 5 min. 6-8 µL of the samples was loaded into each well. The gels were run at 30-35 mA for 30-50 min in 1 × SDS running buffer. The gels were stained with Coomassie Blue dye, and destained with distilled water under microwave irradiation.

2.4 Digestion of antibodies into (Fab')$_2$ or Fc - overview

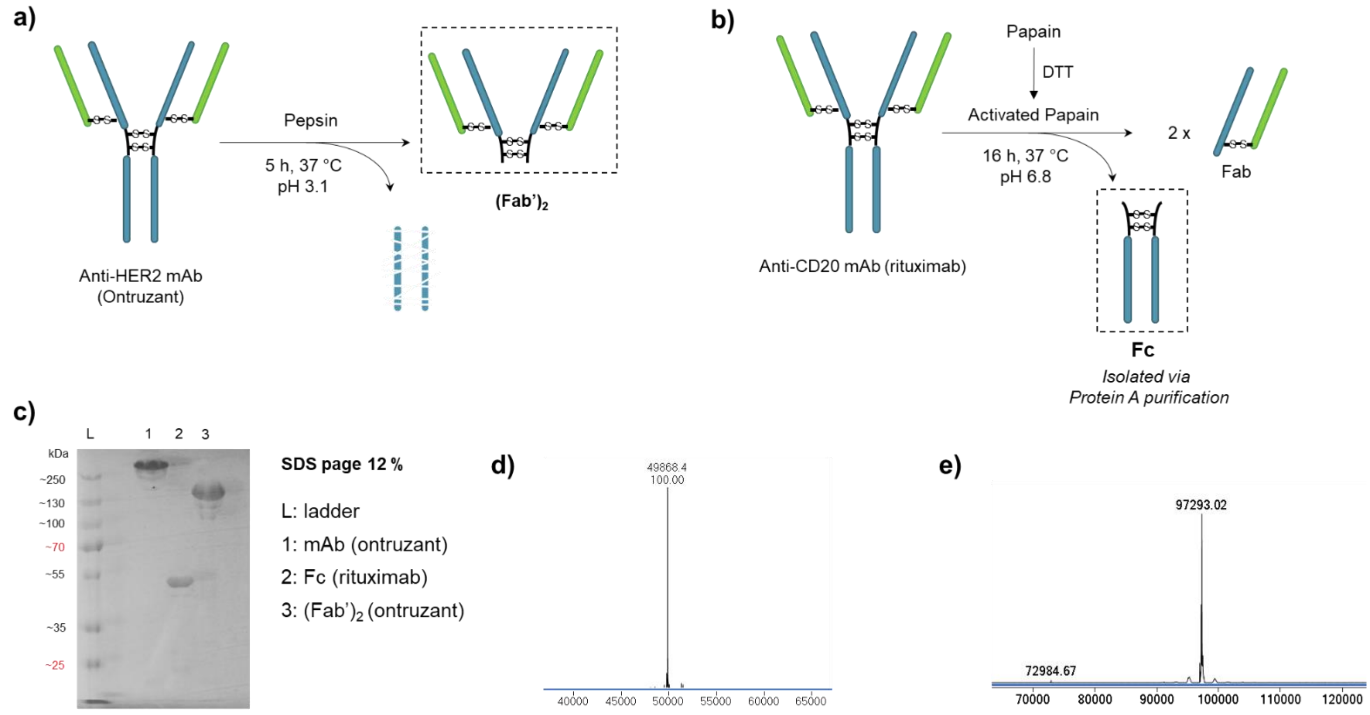

Figure 1 | Overview of digestion of monoclonal antibodies (mAbs) to their corresponding (Fab')$_2$ or Fc and Fab fragments. a) Protocol used for digestion of anti-HER2 mAb (Trastuzumab/Ontruzant). The Fc fragment was removed by pepsin digestion and (Fab')$_2$ isolated via filtration. b) Digestion protocol used in the case of anti-CD20 mAb (Rituximab). Papain, pre-activated with 10 mM DTT, was used to generate Fab and Fc fragments of the parent mAb. These were then separated by protein A purification. c) SDS-PAGE of protein generated by antibody digestion. L: Ladder, Lane 1: native mAb$_{HER2}$. Lane 2: Fc$_{CD20}$ ***29****, Lane 3: (Fab')$_{2\ HER2}$* ***22****. d) LC-MS analysis of Fc$_{CD20}$* ***29****. Expected mass : 49868 Da, Observed mass: 49868 Da. e) LC-MS analysis of (Fab')$_{2\ HER2}$* ***22*** *Expected mass: 97303 Da, Observed mass: 97293 Da.*

2.5 Digestion of anti-HER2 mAb (Ontruzant/Trastuzumab) – generation of anti-HER2 (Fab')$_2$

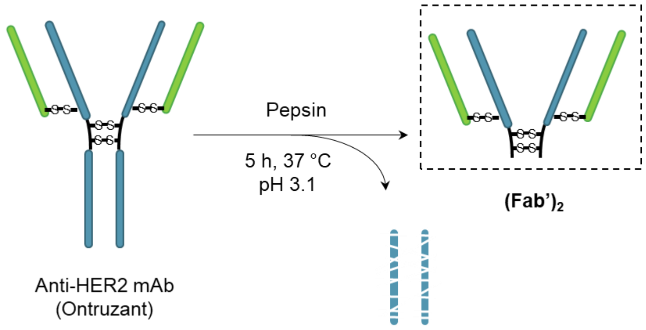

The mAb sample (20 mg) was buffer exchanged into sodium pepsin digest buffer (20 mM NaOAc, pH 3.1, 3 x 15 min, 4000 rpm). Immobilized pepsin (470 µL / 10 mg antibody) was washed 4 times with pepsin digest buffer and the mAb solution (1 mL, 130 µM) was added to this. The mixture was incubated for 5 h at 37 °C under constant agitation (1100 rpm). The resin was separated from the digest using a filter column and washed 3 times with BBS pH 8 buffer (25 mM borate, 25 mM NaCl, pH 8.0, 2 mM EDTA). The digest F(ab')$_2$ solution was combined with the washes and buffer exchanged into BBS pH 8 buffer (10 kDa MWCO Vivaspin) to get rid of small compounds resulting from Fc digestion. Volume was adjusted to 500 µL (14.4 mg/mL, 140 µM).

Representative yield: 54% (Fab')$_{2\_HER2}$

2.6 Digestion of anti-CD20 mAb (Rituximab) – generation of Fc

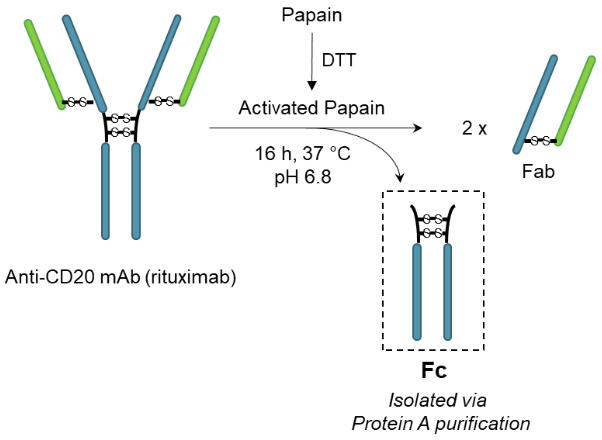

Immobilised papain (0.3 mL, 0.25 mg/mL) was activated with 10 mm DTT in papain digest buffer (50 mM phosphate, 1 mM EDTA, 150 mM NaCl, pH 6.8) under constant agitation (1100 rpm) for 1 h at 25 °C. The resin was washed with papain digest buffer (without DTT) four times and rituximab (3 mg in 0.5 mL of papain digest buffer) was added. The mixture was incubated for 16 h at 37 °C under constant agitation (1100 rpm). Then the resin was separated from the digest using a filter column and washed with Pierce™ Protein A Binding Buffer three times. The digest was combined with the washes and the buffer was exchanged completely for Pierce™ Protein A Binding Buffer and the volume adjusted to 1.5 mL. The Fab and Fc were then separated by protein A purification following manufacturer's protocol, then buffer exchanged in BBS buffer. Representative yield: 31 % Fc_{CD20}.

2.7 Protein A purification

The sample was applied to a NAb™ protein A column (Thermo Scientific) and incubated at RT with end-over-end mixing for 10 min. The unbound Fab fraction was eluted by washing the column four times with Pierce™ Protein A Binding Buffer. The bound Fc fraction was eluted four times by washing the column with Pierce™ IgG elution buffer or 0.1 M glycine buffer (pH 2.5), which was neutralised with 10 % (V/V) of a 1.5 M Tris base, pH 8.8 solution. The Fab and Fc solutions were buffer exchanged into BBS buffer.

2.8 General protocol for the reduction of mAb, Fab or Fc

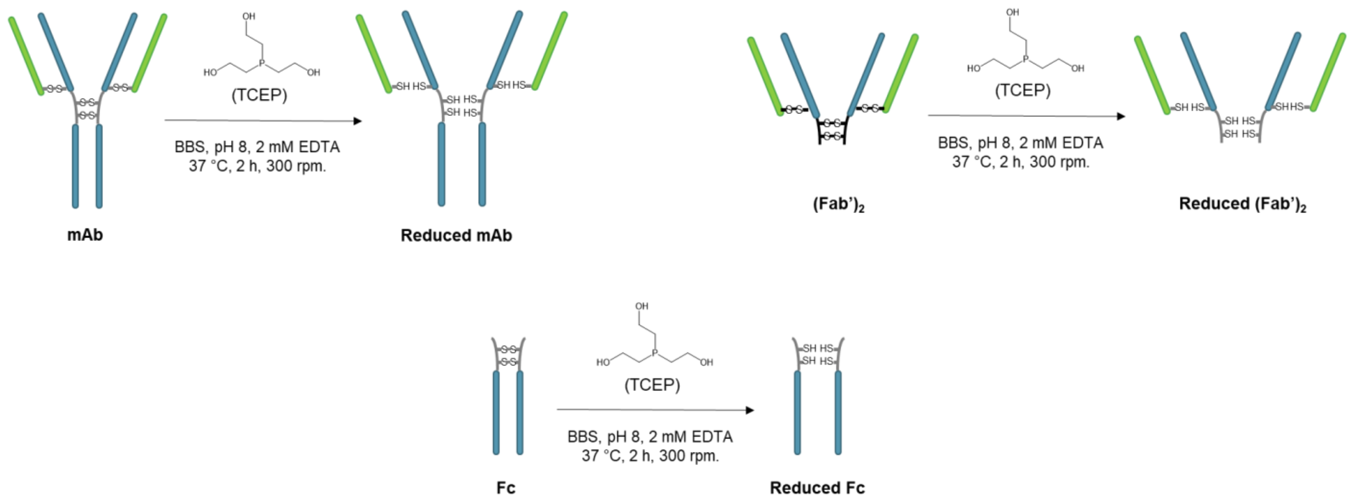

A solution of 10 mM TCEP was prepared by dissolving TCEP-HCl (2.86 mg) in 5 × BBS (1 mL). mAb, (Fab')$_2$ or Fc were prepared in BBS pH 8, 2 mM EDTA (resulting solution ~20 µM), followed by addition of 10 molar equivalents of TCEP solution (10 mM in 5 × BBS pH 8). The mixture was incubated for 90-120 min at 37 °C under constant agitation (300 rpm). The buffer was then exchanged for BBS pH 8, 2 mM EDTA (1 x Zeba spin 7 MCWO unsalting procedure, followed by 1 x Zeba spin 7 MCWO buffer-swap procedure. Alternatively, if sample volume was superior to 120 µL, buffer-swap was realised in 3 washing cycles with Vivaspin 10 MCWO). NB.: The 5 × BBS was employed to maintain the pH of the solution at 8, as TCEP-HCl is strongly acidic.

2.9 General protocol for the re-bridging of mAb and (Fab')$_2$ with trifunctional dual bridging linker

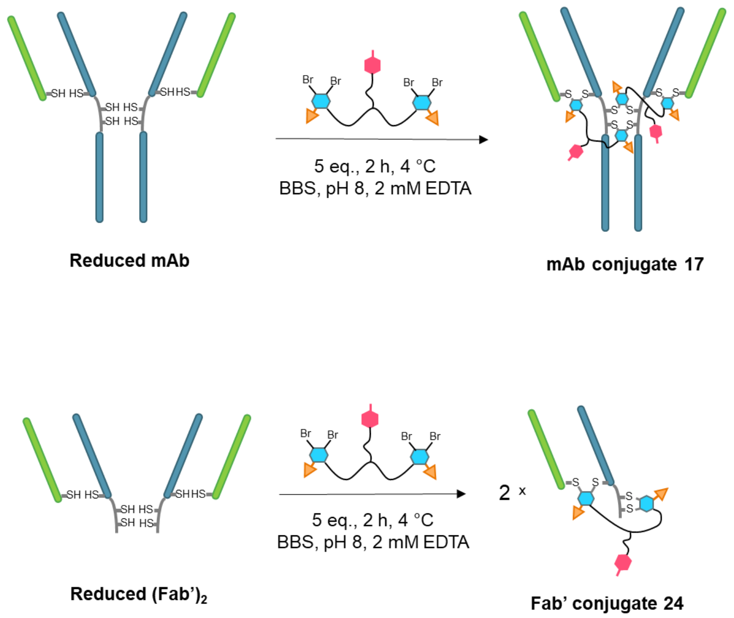

To a solution of reduced mAb or (Fab')$_2$ (100 µL, 20 µM) in BBS pH 8, 2 mM EDTA, was added 5 molar equivalents of dual bridging linker **16** (10 mM in DMF) and the mixture incubated at 4 °C over 120 min, to yield mAb conjugate **17** and Fab' conjugate **24** respectively. The purity of the samples were assessed by non-reducing SDS-PAGE and high-resolution LC-MS. Representative yields: 74 % mAb **17,** 77 % Fab' conjugate **24**.

2.10 General protocol for the re-bridging of Fc with trifunctional dual bridging linker

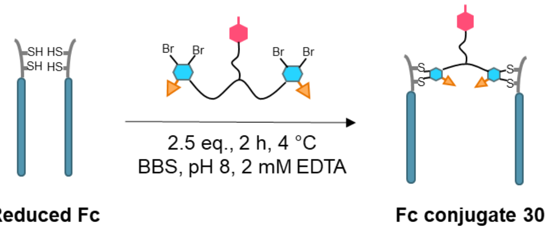

To a solution of reduced Fc (100 µL, 20 µM) in BBS pH 8, 2mM EDTA, was added 2.5 equivalents of dual bridging linker **16** (10 mM in DMF) and the mixture incubated at 4 °C over 120 min, to yield Fc conjugate **30**. The purity of the sample was assessed by non-reducing SDS-PAGE and high-resolution LC-MS. Representative yield: 75 % Fc conjugate **30**.

2.11 Structures and mass of commercial clickable payloads

- Structure and mass of commercial **BCN-fluorescein**

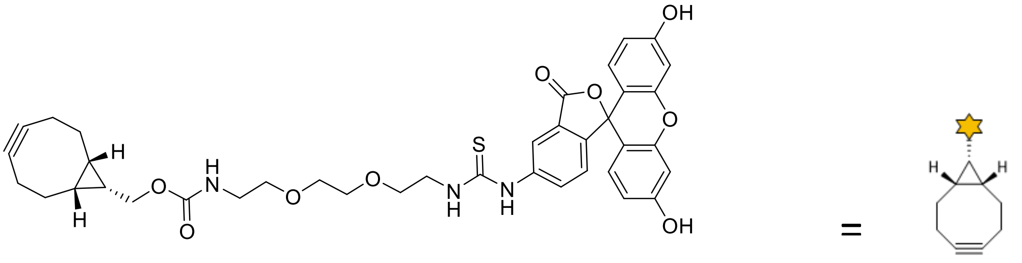

"BCN-fluorescein"

Exact Mass: 713,24
Molecular Weight: 713,80

- Structure and mass of commercial **DBCO-rhodamine**

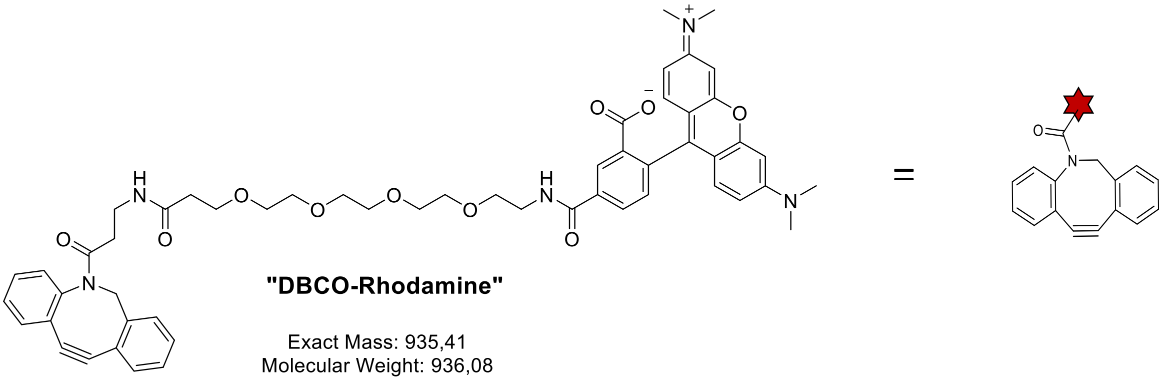

"DBCO-Rhodamine"

Exact Mass: 935,41
Molecular Weight: 936,08

- Structure and mass of commercial **TCO-PEG$_4$-biotin**

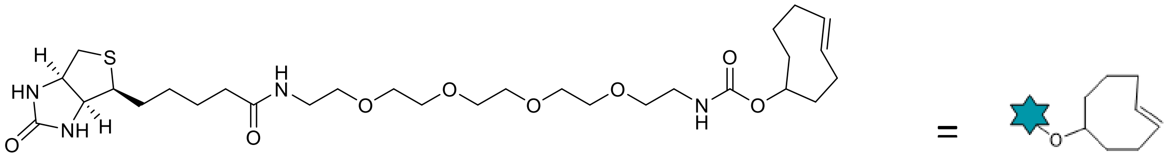

"TCO-Biotin"

Exact Mass: 614,33
Molecular Weight: 614,80

- Structure and mass of commercial **TCO-Cy5**

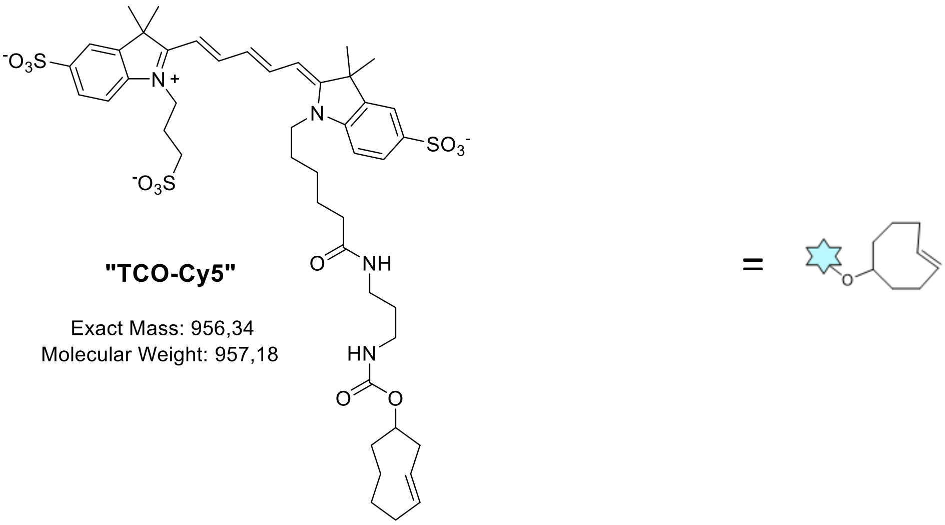

"TCO-Cy5"

Exact Mass: 956,34
Molecular Weight: 957,18

2.12 Click reaction procedures with **BCN-fluorescein**

- Trastu (mAb_{HER2}) conjugate **18**

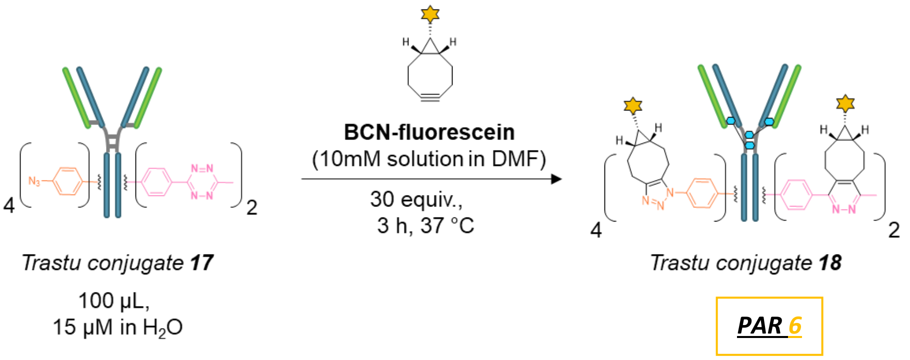

To 100 µL of Trastu conjugate **17** at 15 µM in H_2O was added 30 equiv. (45 nmol, 4.5 µL) of a BCN-fluorescein solution (10 mM in DMF). The resulting mixture was incubated for 3 h at 37 °C under constant agitation (1100 rpm). After this time, excess BCN-fluorescein was removed *via* ultrafiltration (Zeba™ Spin, ThermoFisher Scientific, 7k MWCO, desalting procedure) and sample was buffer swapped to H_2O (Zeba™ Spin, ThermoFisher Scientific, 7k MWCO, buffer swap procedure) to yield Trastu conjugate **18**, as confirmed by LC-MS analysis.
(For LC-MS analysis, sample was diluted down in H_2O to ~5 µM final concentration. PNGase F was added (0.6 µL) and resulting sample was incubated 12 h at RT before analysis).

- Fab'$_{HER2}$ conjugate **25**

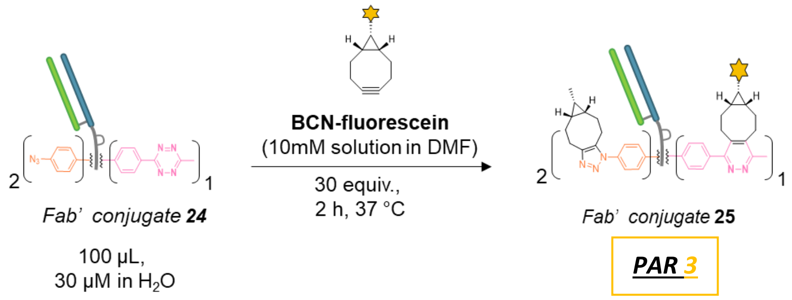

To 100 µL of Fab'$_{HER2}$ conjugate **24** at 30 µM in H_2O was added 15 equiv. (45 nmol, 4.5 µL) of a BCN-fluorescein solution (10 mM in DMF). The resulting mixture was incubated for 3 h at 37 °C under constant agitation (1100 rpm). After this time, excess BCN-fluorescein was removed *via* ultrafiltration (Zeba™ Spin, ThermoFisher Scientific, 7k MWCO, desalting procedure) and sample was buffer swapped to H_2O (Zeba™ Spin, ThermoFisher Scientific, 7k MWCO, buffer swap procedure) to yield Fab'$_{HER2}$ conjugate **25**, as confirmed by LC-MS analysis.
(For LC-MS analysis, sample was diluted down in H_2O to ~5 µM final concentration).

- Fc_{CD20} conjugate **31**

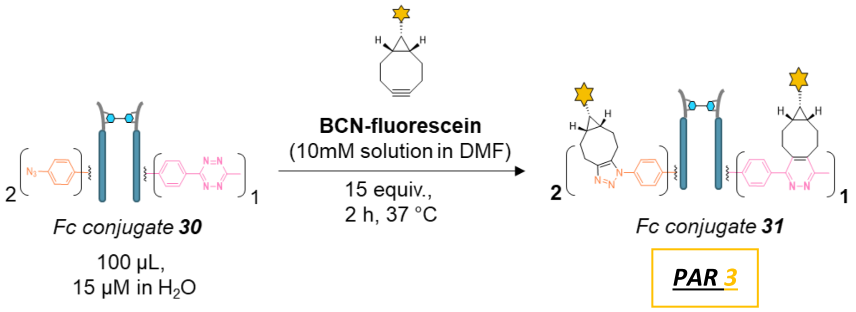

To 100 µL of Fc conjugate **30** at 15 µM in H_2O was added 15 equiv. (22.5 nmol, 2.25 µL) of a BCN-fluorescein solution (10 mM in DMF). The resulting mixture was incubated for 3 h at 37 °C under constant agitation (1100 rpm). After this time, excess BCN-fluorescein was removed *via* ultrafiltration (Zeba™ Spin, ThermoFisher Scientific, 7k MWCO, desalting procedure) and sample was buffer swapped to H_2O (Zeba™ Spin, ThermoFisher Scientific, 7k MWCO, buffer swap procedure) to yield Fc conjugate **31**, as confirmed by LC-MS analysis.
(For LC-MS analysis, sample was diluted down in H_2O to ~5 µM final concentration. PNGase F was added (0.6 µL) and resulting sample was incubated 12 h at RT before analysis).

2.13 Click reaction procedures with **DBCO-rhodamine**

- Trastu (mAb_{HER2}) conjugate **19**

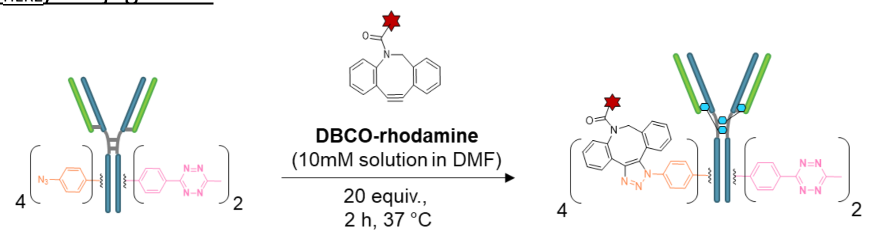

Trastu conjugate ***17*** — 100 µL, 15 µM in H_2O → *Trastu conjugate* **19** — ***PAR 4***

To 100 µL of Trastu conjugate **17** at 15 µM in H_2O was added 20 equiv. (30 nmol, 3 µL) of a DBCO-rhodamine solution (10 mM in DMF). The resulting mixture was incubated for 2 h at 37 °C under constant agitation (1100 rpm). After this time, excess DBCO-rhodamine was removed *via* ultrafiltration (Zeba™ Spin, ThermoFisher Scientific, 7k MWCO, desalting procedure) and sample was buffer swapped to H_2O (Zeba™ Spin, ThermoFisher Scientific, 7k MWCO, buffer swap procedure) to yield Trastu conjugate **19**, as confirmed by LC-MS analysis.
(For LC-MS analysis, sample was diluted down in H_2O to ~5 µM final concentration. PNGase F was added (0.6 µL) and resulting sample was incubated 12 h at RT before analysis).

- Fab'$_{HER2}$ conjugate **26**

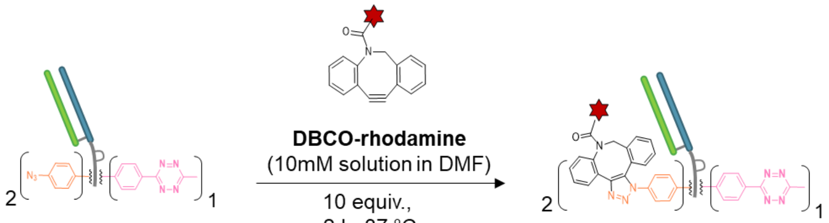

Fab' conjugate ***24*** — 100 µL, 30 µM in H_2O → *Fab' conjugate* ***26*** — ***PAR 2***

To 100 µL of Fab'$_{HER2}$ conjugate **24** at 30 µM in H_2O was added 10 equiv. (30 nmol, 3 µL) of a DBCO-rhodamine solution (10 mM in DMF). The resulting mixture was incubated for 2 h at 37 °C under constant agitation (1100 rpm). After this time, excess DBCO-rhodamine was removed *via* ultrafiltration (Zeba™ Spin, ThermoFisher Scientific, 7k MWCO, desalting procedure) and sample was buffer swapped to H_2O (Zeba™ Spin, ThermoFisher Scientific, 7k MWCO, buffer swap procedure) to yield Fab'$_{HER2}$ conjugate **26**, as confirmed by LC-MS analysis.
(For LC-MS analysis, sample was diluted down in H_2O to ~5 µM final concentration).

- Fc_{CD20} conjugate **32**

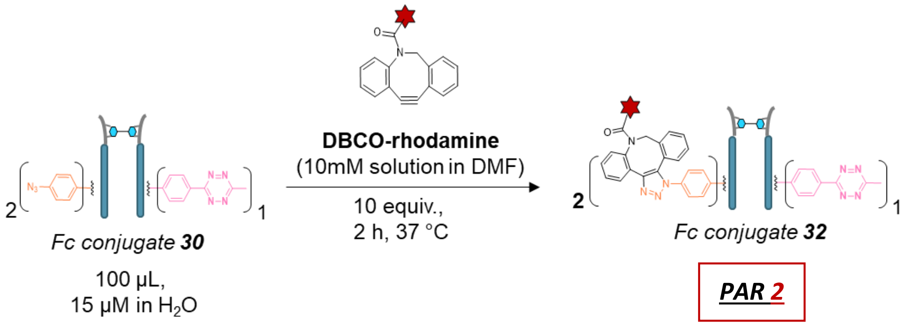

Fc conjugate ***30*** — 100 µL, 15 µM in H_2O → *Fc conjugate* ***32*** — ***PAR 2***

To 100 µL of Fc conjugate **30** at 15 µM in H_2O was added 10 equiv. (15 nmol, 1.5 µL) of a DBCO-rhodamine solution (10 mM in DMF). The resulting mixture was incubated for 2 h at 37 °C under constant agitation (1100 rpm). After this time, excess DBCO-rhodamine was removed *via* ultrafiltration (Zeba™ Spin, ThermoFisher Scientific, 7k MWCO, desalting procedure) and sample was buffer swapped to H_2O (Zeba™ Spin, ThermoFisher Scientific, 7k MWCO, buffer swap procedure) to yield Fc conjugate **32**, as confirmed by LC-MS analysis.
(For LC-MS analysis, sample was diluted down in H_2O to ~5 µM final concentration. PNGase F was added (0.6 µL) and resulting sample was incubated 12 h at RT before analysis).

2.14 Click reaction procedures with **TCO-biotin**

- Trastu (mAb$_{HER2}$) conjugate **20**

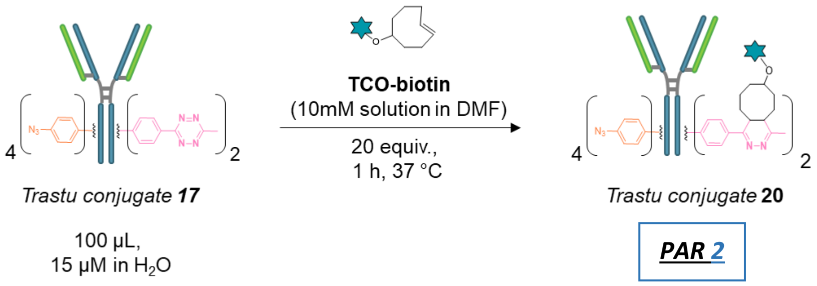

To 100 µL of Trastu conjugate **17** at 15 µM in H_2O was added 20 equiv. (30 nmol, 3 µL) of a TCO-biotin solution (10 mM in DMF). The resulting mixture was incubated for 1 h at 37 °C under constant agitation (1100 rpm). After this time, excess TCO-biotin was removed *via* ultrafiltration (Zeba™ Spin, ThermoFisher Scientific, 7k MWCO, desalting procedure) and sample was buffer swapped to H_2O (Zeba™ Spin, ThermoFisher Scientific, 7k MWCO, buffer swap procedure) to yield Trastu conjugate **20**, as confirmed by LC-MS analysis.
(For LC-MS analysis, sample was diluted down in H_2O to ~5 µM final concentration. PNGase F was added (0.6 µL) and resulting sample was incubated 12 h at RT before analysis).

- Fab'$_{HER2}$ conjugate **27**

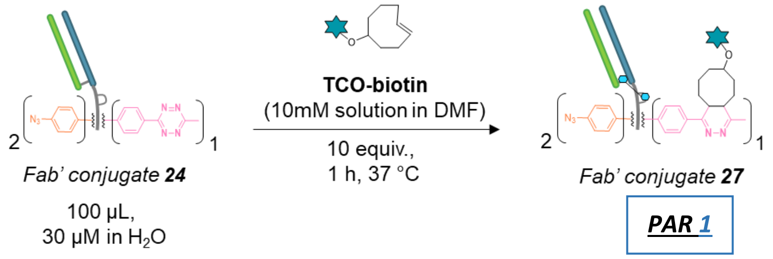

To 100 µL of Fab'$_{HER2}$ conjugate **24** at 30 µM in H_2O was added 10 equiv. (30 nmol, 3 µL) of a TCO-biotin solution (10 mM in DMF). The resulting mixture was incubated for 1 h at 37 °C under constant agitation (1100 rpm). After this time, excess TCO-biotin was removed *via* ultrafiltration (Zeba™ Spin, ThermoFisher Scientific, 7k MWCO, desalting procedure) and sample was buffer swapped to H_2O (Zeba™ Spin, ThermoFisher Scientific, 7k MWCO, buffer swap procedure) to yield Fab'$_{HER2}$ conjugate **27**, as confirmed by LC-MS analysis.
(For LC-MS analysis, sample was diluted down in H_2O to ~5 µM final concentration).

- Fc$_{CD20}$ conjugate **33**

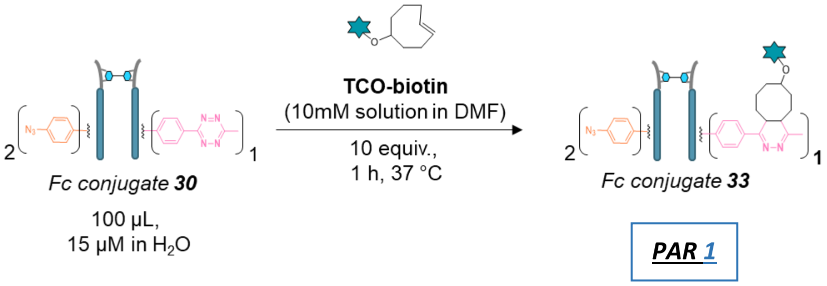

To 100 µL of Fc conjugate **30** at 15 µM in H_2O was added 10 equiv. (15 nmol, 1.5 µL) of a TCO-biotin solution (10 mM in DMF). The resulting mixture was incubated for 1 h at 37 °C under constant agitation (1100 rpm). After this time, excess TCO-biotin was removed *via* ultrafiltration (Zeba™ Spin, ThermoFisher Scientific, 7k MWCO, desalting procedure) and sample was buffer swapped to H_2O (Zeba™ Spin, ThermoFisher Scientific, 7k MWCO, buffer swap procedure) to yield Fc conjugate **33**, as confirmed by LC-MS analysis.
(For LC-MS analysis, sample was diluted down in H_2O to ~5 µM final concentration. PNGase F was added (0.6 µL) and resulting sample was incubated 12 h at RT before analysis).

2.14 Click reaction procedures with **TCO-Cyanine5**

- Trastu (mAb$_{HER2}$) conjugate S**20**

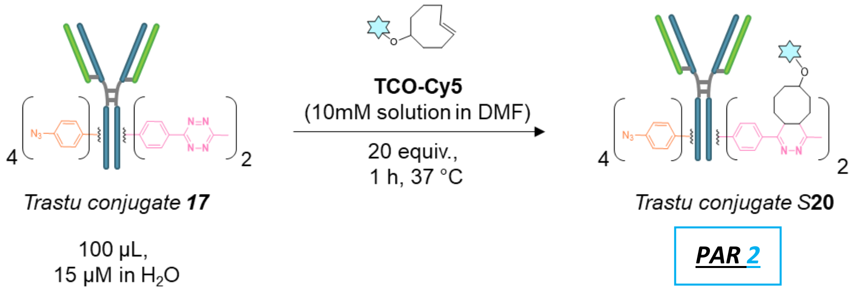

To 100 μL of Trastu conjugate **17** at 15 μM in H_2O was added 20 equiv. (30 nmol, 3 μL) of a TCO-Cy5 solution (10 mM in DMF). The resulting mixture was incubated for 1 h at 37 °C under constant agitation (1100 rpm). After this time, excess TCO-Cy5 was removed *via* ultrafiltration (Zeba™ Spin, ThermoFisher Scientific, 7k MWCO, desalting procedure) and sample was buffer swapped to H_2O (Zeba™ Spin, ThermoFisher Scientific, 7k MWCO, buffer swap procedure) to yield Trastu conjugate S**20**, as confirmed by LC-MS analysis.
(For LC-MS analysis, sample was diluted down in H_2O to ~5 μM final concentration. PNGase F was added (0.6 μL) and resulting sample was incubated 12 h at RT before analysis).

- Fab'$_{HER2}$ conjugate S**27**

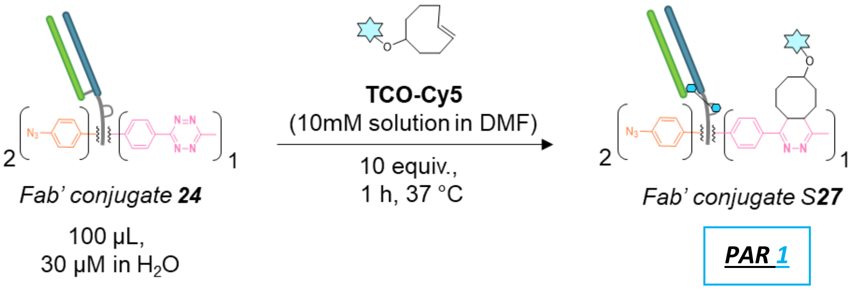

To 100 μL of Fab'$_{HER2}$ conjugate **24** at 30 μM in H_2O was added 10 equiv. (30 nmol, 3 μL) of a TCO-Cy5 solution (10 mM in DMF). The resulting mixture was incubated for 1 h at 37 °C under constant agitation (1100 rpm). After this time, excess TCO-Cy5 was removed *via* ultrafiltration (Zeba™ Spin, ThermoFisher Scientific, 7k MWCO, desalting procedure) and sample was buffer swapped to H_2O (Zeba™ Spin, ThermoFisher Scientific, 7k MWCO, buffer swap procedure) to yield Fab'$_{HER2}$ conjugate S**27**, as confirmed by LC-MS analysis.
(For LC-MS analysis, sample was diluted down in H_2O to ~5 μM final concentration).

- Fc$_{CD20}$ conjugate S**33**

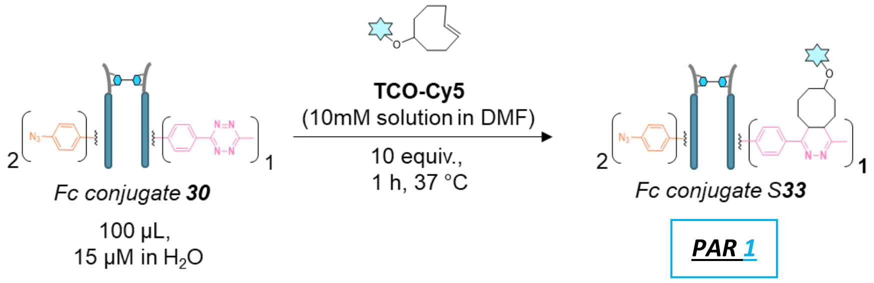

To 100 μL of Fc conjugate **30** at 15 μM in H_2O was added 10 equiv. (15 nmol, 1.5 μL) of a TCO-Cy5 solution (10 mM in DMF). The resulting mixture was incubated for 1 h at 37 °C under constant agitation (1100 rpm). After this time, excess TCO-Cy5 was removed *via* ultrafiltration (Zeba™ Spin, ThermoFisher Scientific, 7k MWCO, desalting procedure) and sample was buffer swapped to H_2O (Zeba™ Spin, ThermoFisher Scientific, 7k MWCO, buffer swap procedure) to yield Fc conjugate S**33**, as confirmed by LC-MS analysis.

(For LC-MS analysis, sample was diluted down in H_2O to ~5 µM final concentration. PNGase F was added (0.6 µL) and resulting sample was incubated 12 h at RT before analysis).

2.15 Successive click reaction procedures with **DBCO-rhodamin** and **BCN-fluorescein**

- Trastu (mAb_{HER2}) conjugate **21**

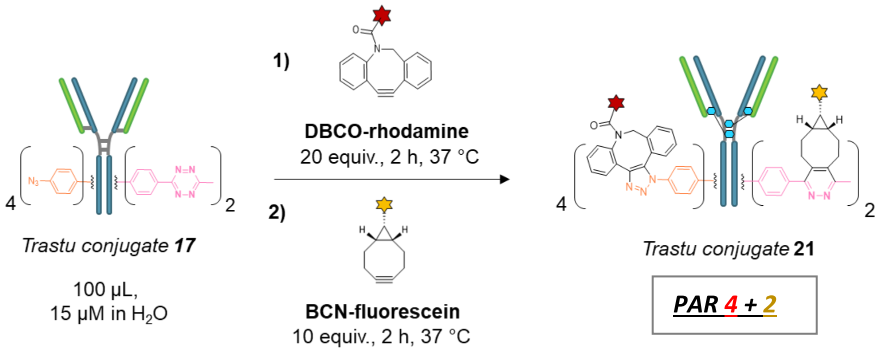

To 100 µL of Trastu conjugate **17** at 15 µM in H_2O was added 20 equiv. (30 nmol, 3 µL) of a DBCO-rhodamine solution (10 mM in DMF). The resulting mixture was incubated for 2 h at 37 °C under constant agitation (1100 rpm). After this time, excess DBCO-rhodamine was removed *via* ultrafiltration (Zeba™ Spin, ThermoFisher Scientific, 7k MWCO, desalting procedure) and sample was buffer swapped to H_2O (Zeba™ Spin, ThermoFisher Scientific, 7k MWCO, buffer swap procedure), for a resulting sample of 100µL at ~15 µM (which corresponds to Trastu conjugate **19** at that step). A solution of BCN-fluorescein (10 mM) was added (10 equiv., 1 µL) and resulting mixture was incubated for another 2 h at 37 °C under constant agitation (1100 rpm). After this time, excess BCN-fluorescein was removed *via* ultrafiltration (Zeba™ Spin, ThermoFisher Scientific, 7k MWCO, desalting procedure) and sample was buffer swapped to H_2O (Zeba™ Spin, ThermoFisher Scientific, 7k MWCO, buffer swap procedure) to yield Fc conjugate **21**, as confirmed by LC-MS analysis.
(For LC-MS analysis, sample was diluted down in H_2O to ~5 µM final concentration. PNGase F was added (0.6 µL) and resulting sample was incubated 12 h at RT before analysis).

- Fab'$_{HER2}$ conjugate **28**

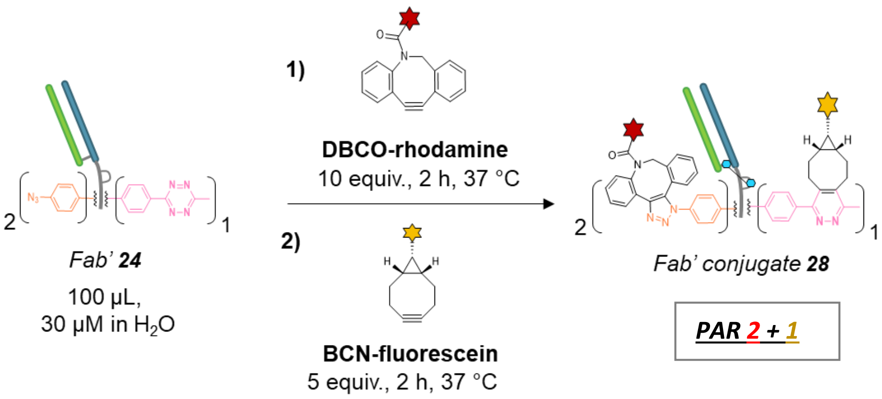

To 100 µL of Fab'$_{HER2}$ conjugate **24** at 30 µM in H_2O was added 10 equiv. (30 nmol, 3 µL) of a DBCO-rhodamine solution (10 mM in DMF). The resulting mixture was incubated for 2 h at 37 °C under constant agitation (1100 rpm). After this time, excess DBCO-rhodamine was removed *via* ultrafiltration (Zeba™ Spin, ThermoFisher Scientific, 7k MWCO, desalting procedure) and sample was buffer swapped to H_2O (Zeba™ Spin, ThermoFisher Scientific, 7k MWCO, buffer swap procedure) for a resulting sample of 100 µL at ~30 µM (which corresponds to Fab' conjugate **26** at that step). A solution of BCN-fluorescein (10 mM) was added (5 equiv., 1.5 µL) and resulting mixture was

incubated for another 2 h at 37 °C under constant agitation (1100 rpm). After this time, excess BCN-fluorescein was removed *via* ultrafiltration (Zeba™ Spin, ThermoFisher Scientific, 7k MWCO, desalting procedure) and sample was buffer swapped to H_2O (Zeba™ Spin, ThermoFisher Scientific, 7k MWCO, buffer swap procedure) to yield Fc conjugate **28**, as confirmed by LC-MS analysis.
(For LC-MS analysis, sample was diluted down in H_2O to ~5 µM final concentration).

- Fc_{CD20} conjugate **34**

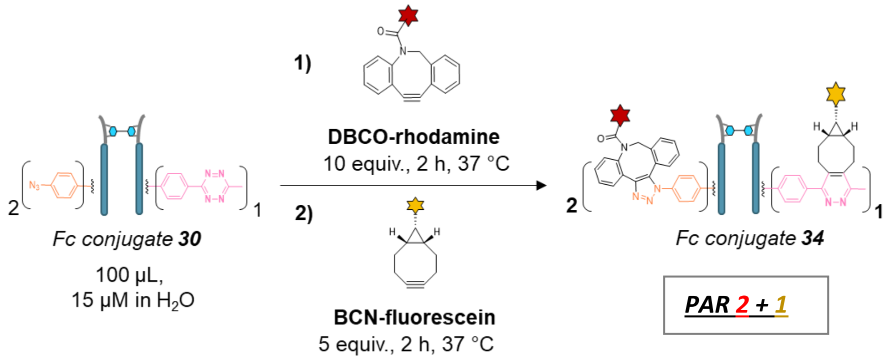

To 100 µL of Fc conjugate **30** at 15 µM in H_2O was added 10 equiv. (15 nmol, 1.5 µL) of a DBCO-rhodamine solution (10 mM in DMF). The resulting mixture was incubated for 2 h at 37 °C under constant agitation (1100 rpm). After this time, excess DBCO-rhodamine was removed *via* ultrafiltration (Zeba™ Spin, ThermoFisher Scientific, 7k MWCO, desalting procedure) and sample was buffer swapped to H_2O (Zeba™ Spin, ThermoFisher Scientific, 7k MWCO, buffer swap procedure) for a resulting sample of 100 µL at ~15 µM (which corresponds to Fc conjugate **32** at that step). A solution of BCN-fluorescein (10 mM) was added (5 equiv., 0.75 µL) and resulting mixture was incubated for another 2 h at 37 °C under constant agitation (1100 rpm). After this time, excess BCN-fluorescein was removed *via* ultrafiltration (Zeba™ Spin, ThermoFisher Scientific, 7k MWCO, desalting procedure) and sample was buffer swapped to H_2O (Zeba™ Spin, ThermoFisher Scientific, 7k MWCO, buffer swap procedure) to yield Fc conjugate **34**, as confirmed by LC-MS analysis.
(For LC-MS analysis, sample was diluted down in H_2O to ~5 µM final concentration. PNGase F was added (0.6 µL) and resulting sample was incubated 12 h at RT before analysis).

3. LC-MS spectra

NB: Given that sensitivity of high m/z was low for mAb conjugates analyses (see mass envelop raw data), the mass of half-antibody species (no desired but present in small proportions due to disulfide scrambling during rebridging) were included as “expected mass”, in order to reinforce confidence in the automatic deconvoluted data generated by the software for mAb species. Manual deconvolution check was realised too.

PNGase enzyme was used for deglycosylation in every mAb- and Fc-conjugates analysis (even if not necessarily shows up on MS spectra).

- <u>mAb Ontruzant/Trastuzumab (commercial)</u>

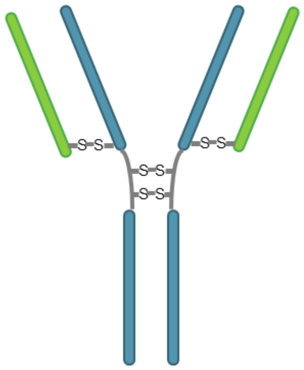

Expected mass: 145189 Da

Observed mass: **145188** Da

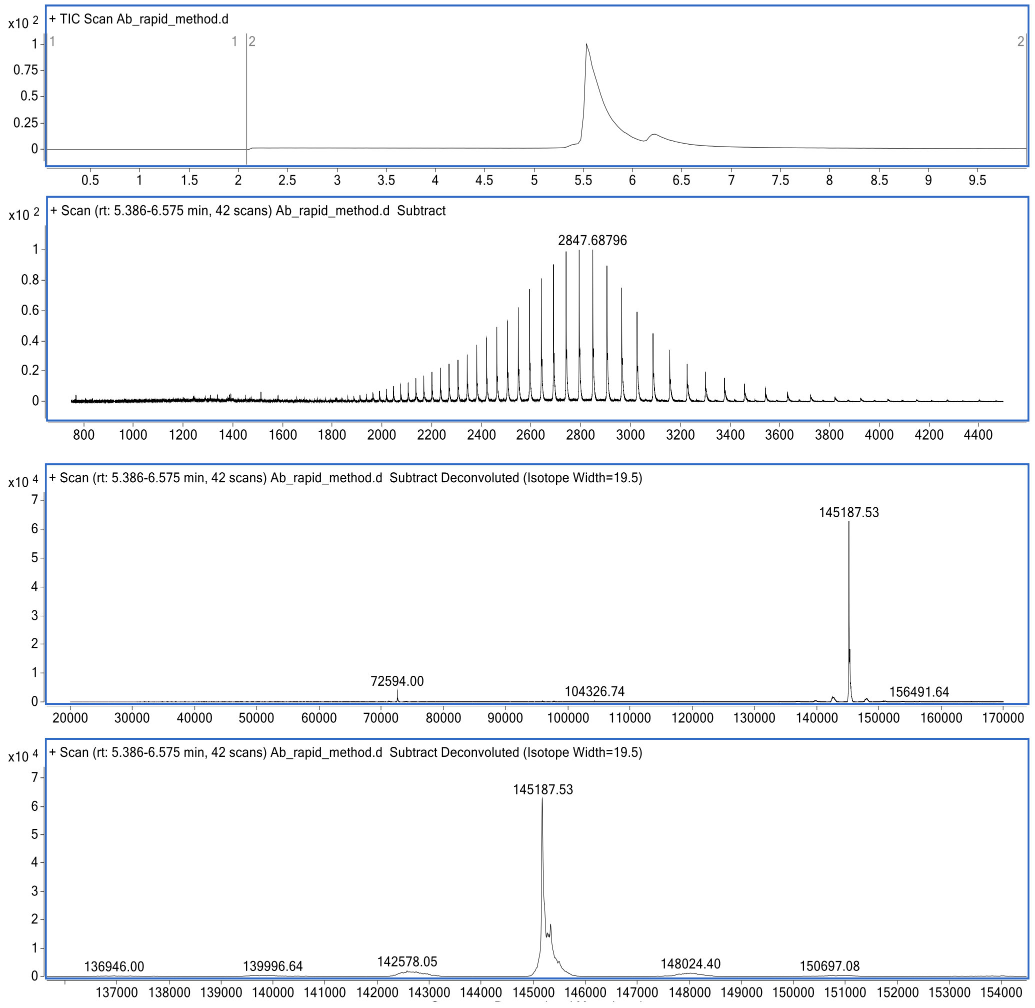

- <u>mAb conjugate **17**</u>

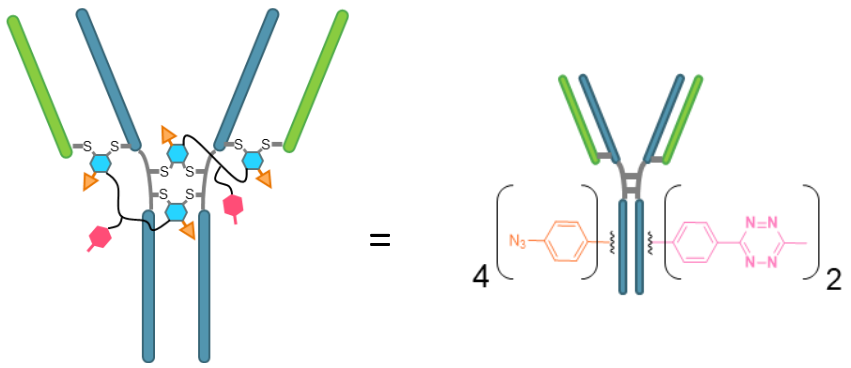

Expected mass: **148197** Da, 74098 Da (re-rebridged half-antibody)
Observed mass: **148203** Da, 74098 Da

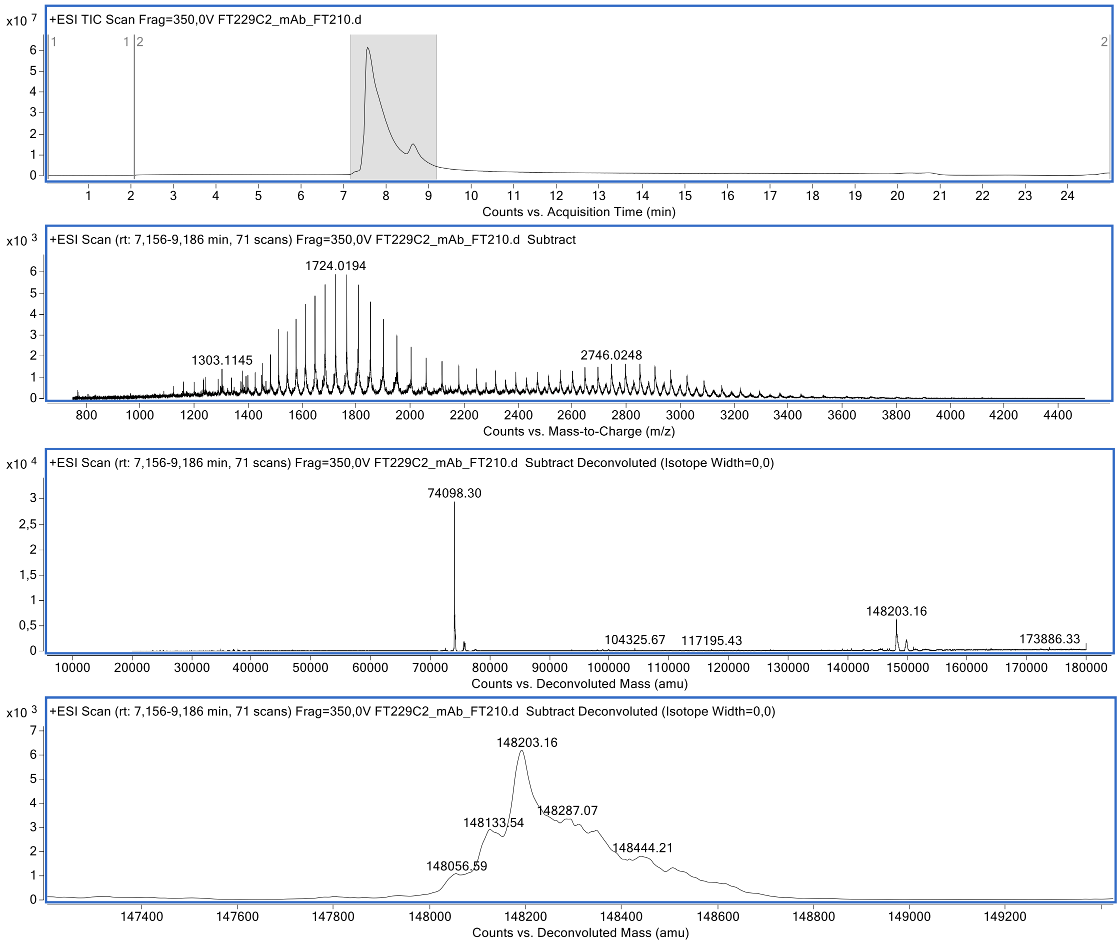

- <u>mAb conjugate **18**</u>

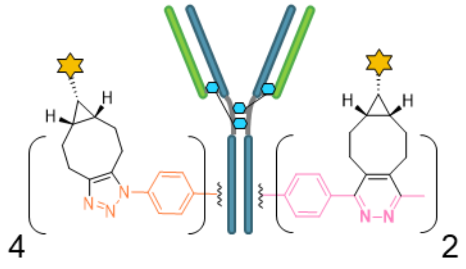

Expected mass: **152425** Da, 76212 Da (modified half-antibody)

Observed mass: **152419** Da, 76223 Da, 34775 Da (➔ PNGase F)

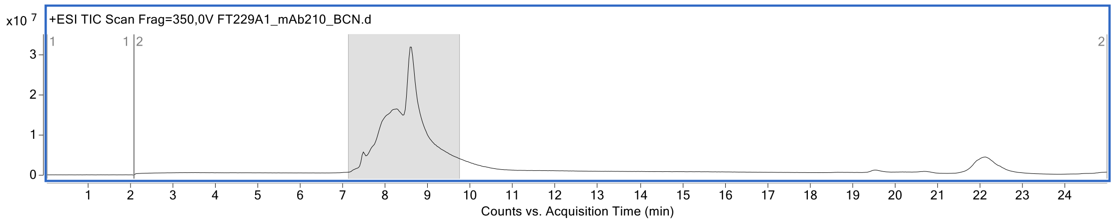

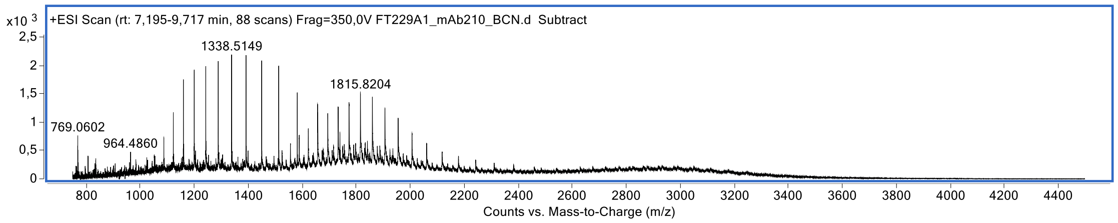

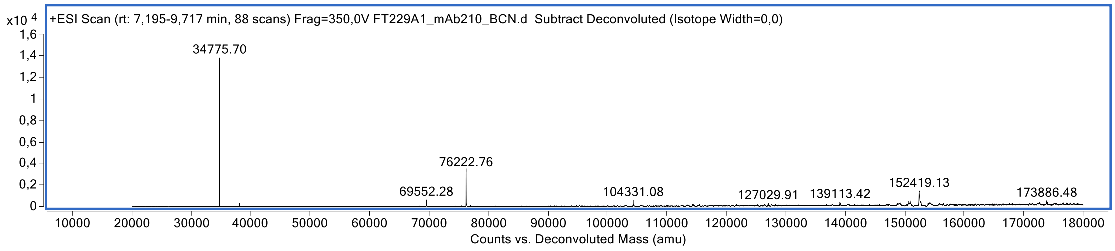

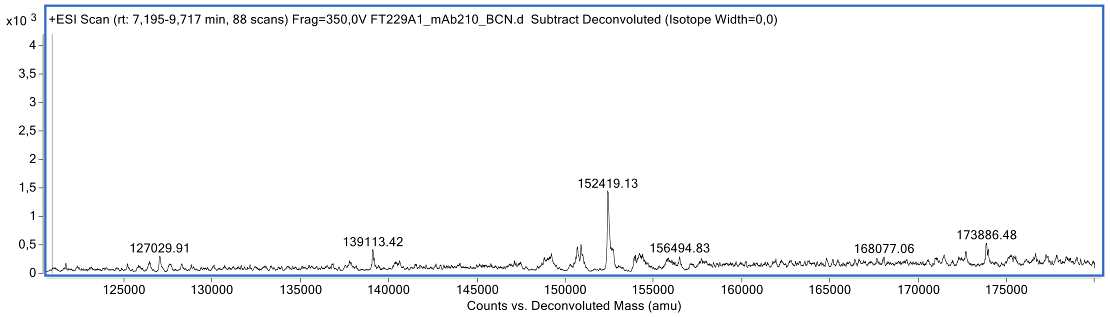

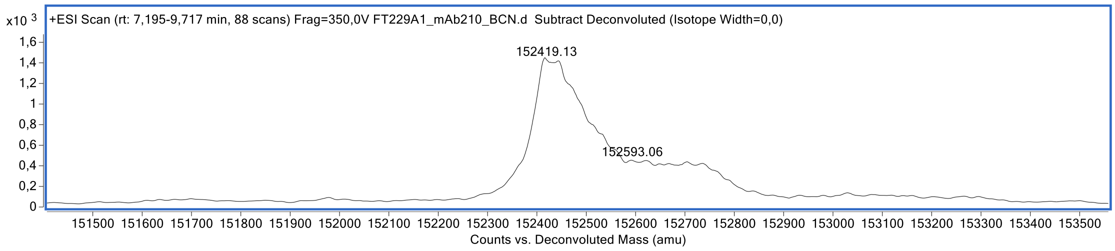

- mAb conjugate **19**

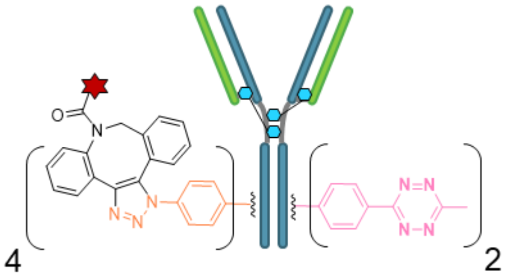

Expected mass: **151941** Da, 75968 Da (modified half-antibody)

Observed mass: **151944** Da, 75976 Da

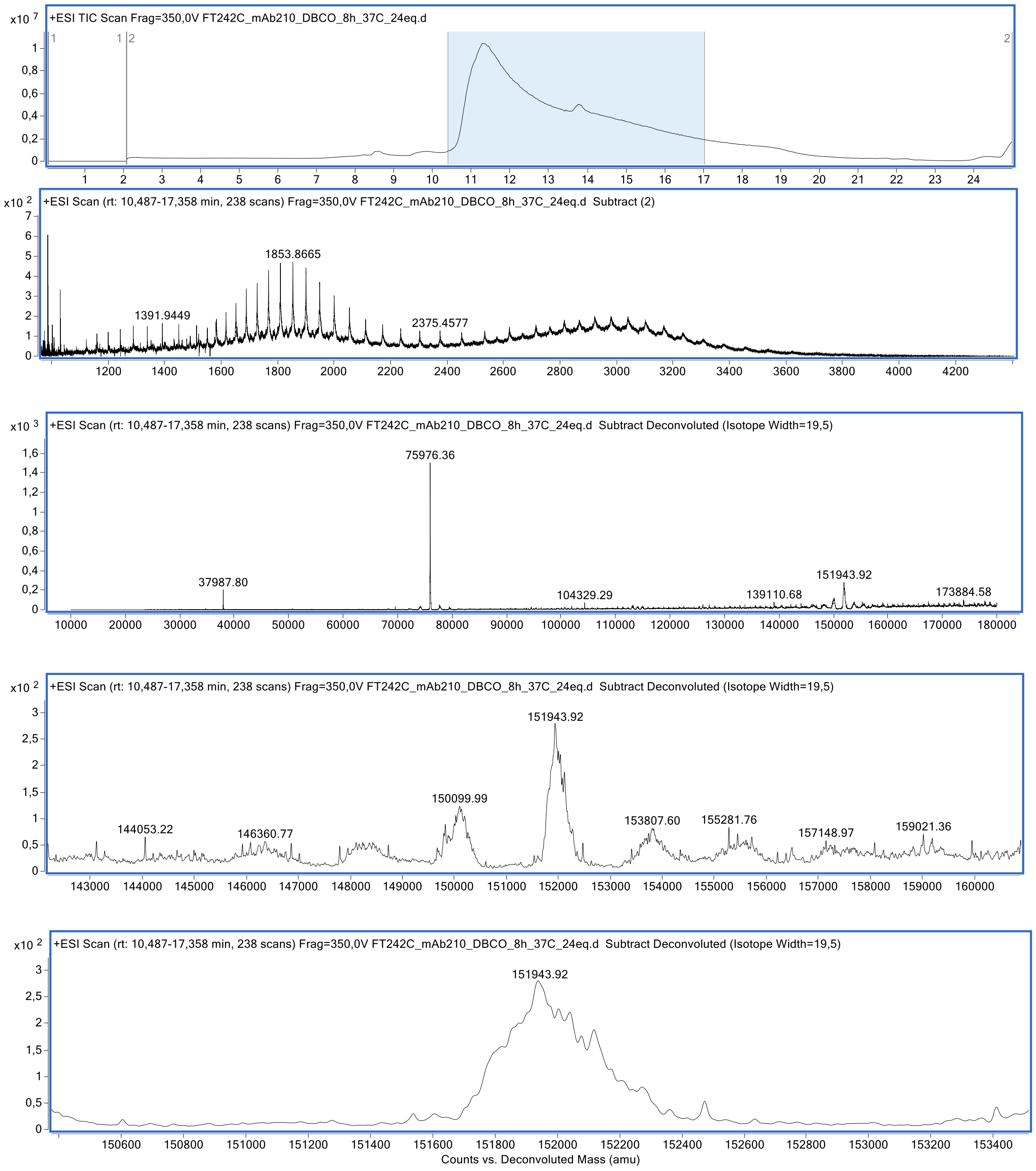

- mAb conjugate **20**

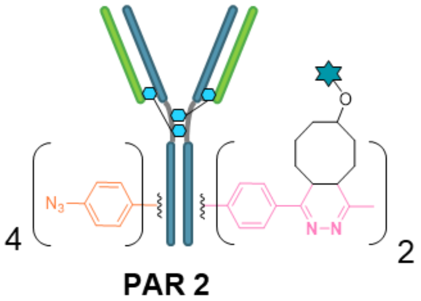

Expected mass: **149375** Da, 74684 Da (modified half-antibody)

Observed mass: **149376** Da, 74684 Da

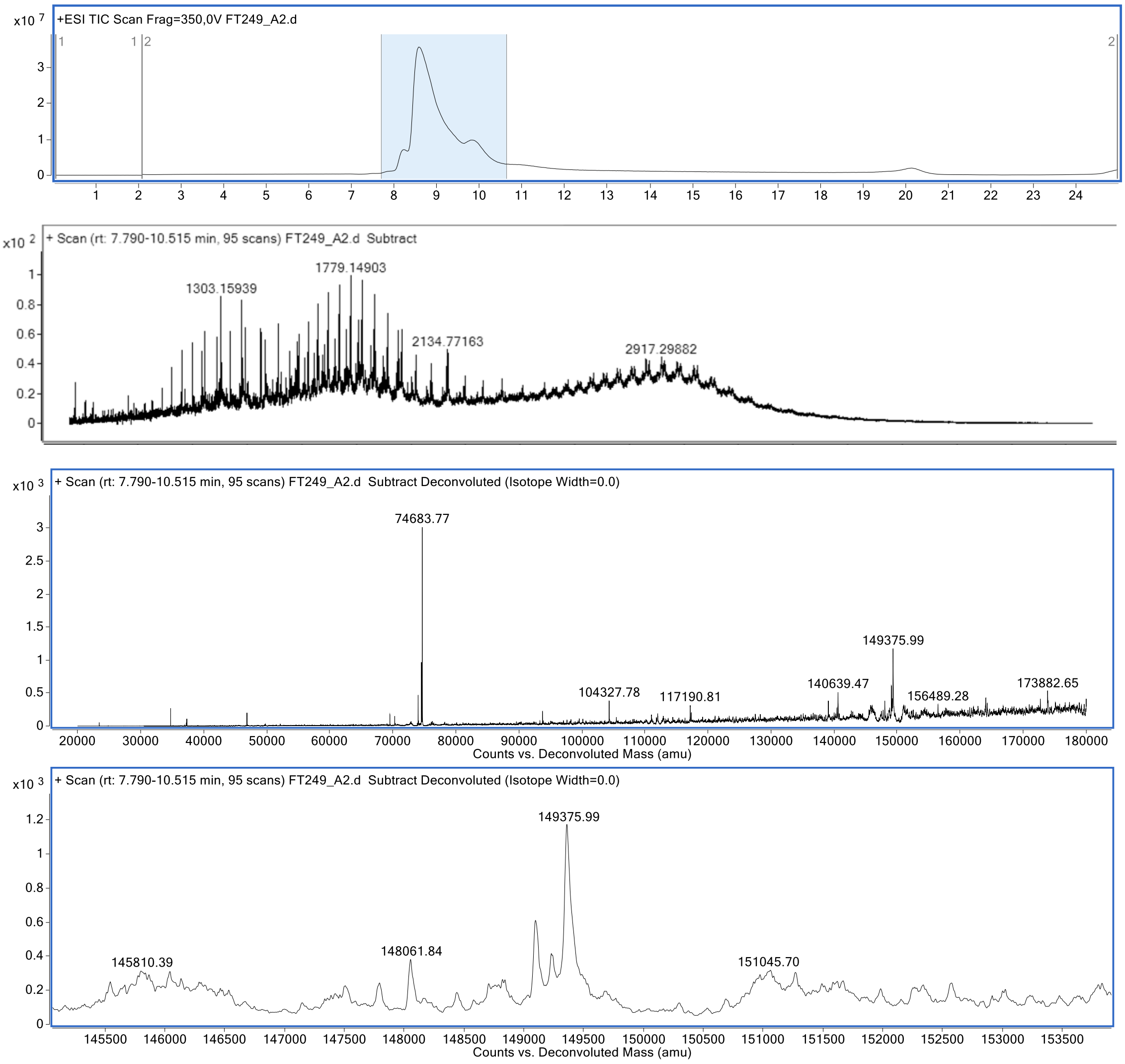

- mAb conjugate **S20**

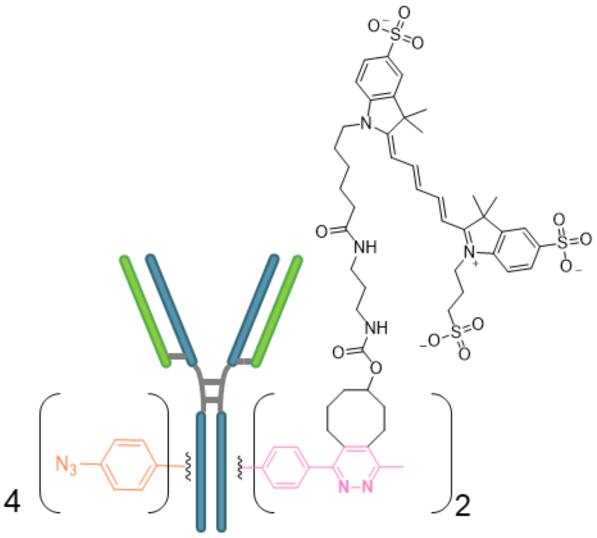

Expected mass: **150059** Da (and up to 4 loss of 131 Da *via* [CH_2-phenyl-N_3] elimination could be observed),[6] 75026 (modified half-antibody, and up to 2 loss of 131 Da *via* [CH_2-phenyl-N_3] elimination could be observed).

Observed mass: **150055** Da, 149803 Da (elimination of 2 x [CH_2-phenyl-N_3]), 149539 Da (elimination of 4 x [CH_2-phenyl-N_3]), 74901 Da (modified half-antibody, with loss of 1 x [CH_2-phenyl-N_3])

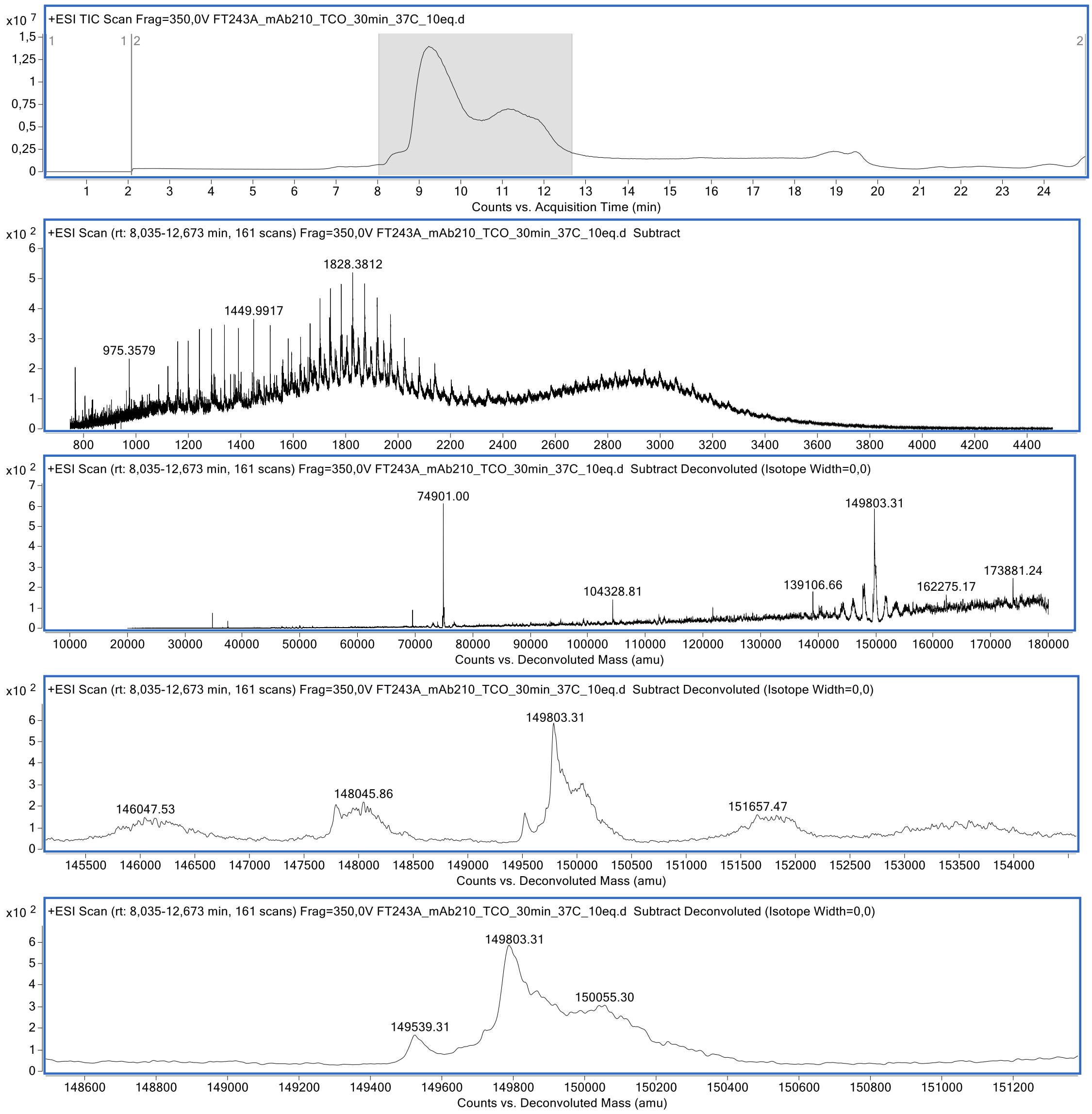

- <u>mAb conjugate **21**</u>

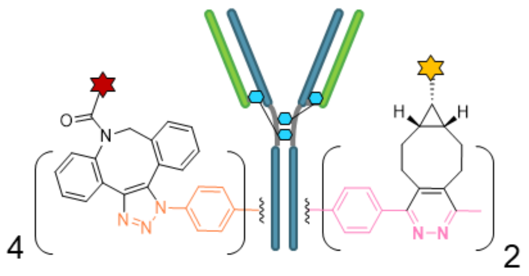

Expected mass: **153313** Da, 76653 Da (modified half-antibody)

Observed mass: **153306** Da, 76657 Da, 34775 Da (PNGase F)

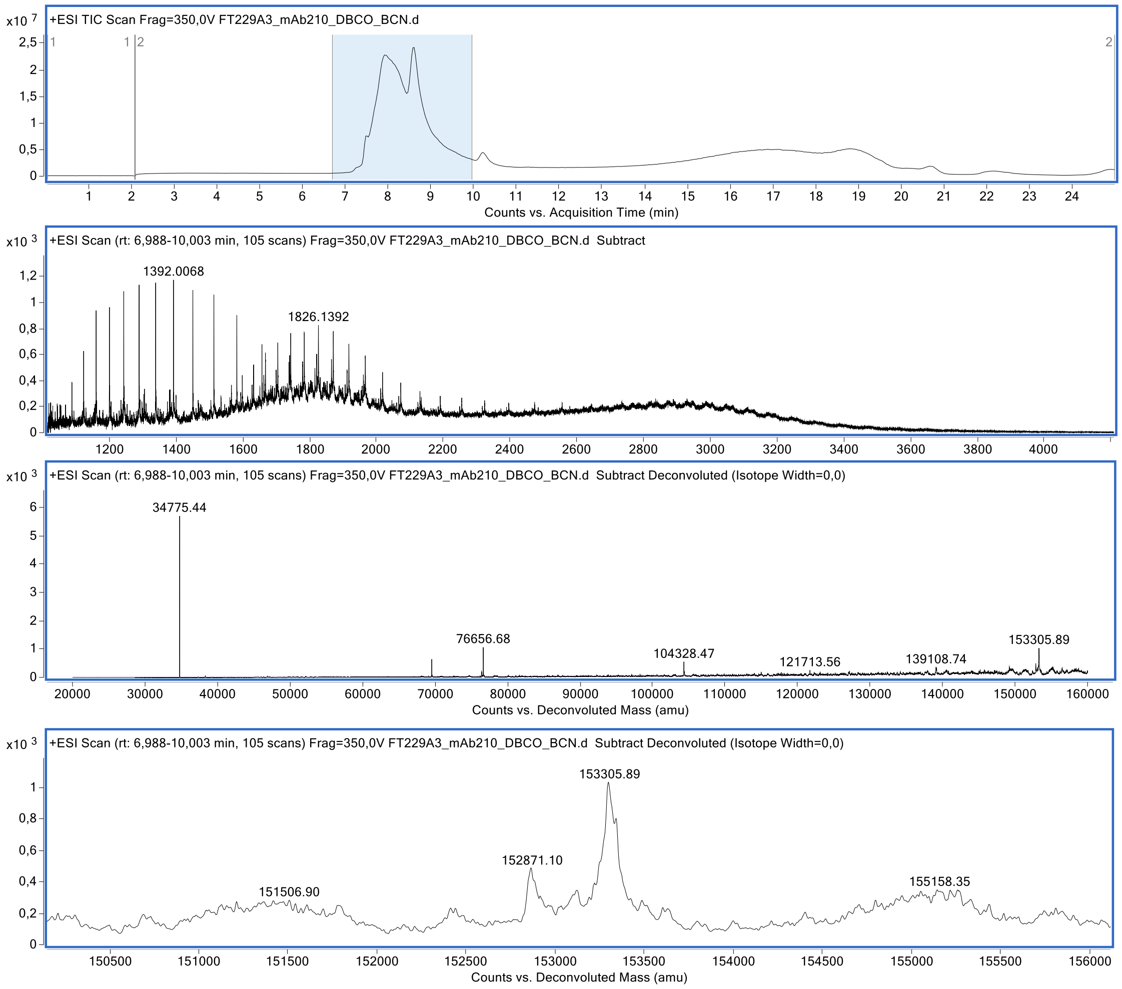

- <u>(Fab')$_2$ **22**</u>

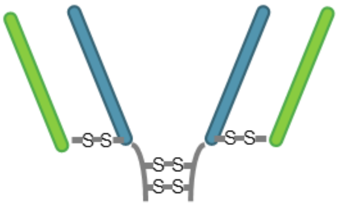

Expected mass: 97303 Da

Observed mass: 97293 Da

Even though not detrimental, two extra peaks are observed (97179 Da and 97067 Da) that correspond to loss of an extra Leucine on one or both HC.[7]

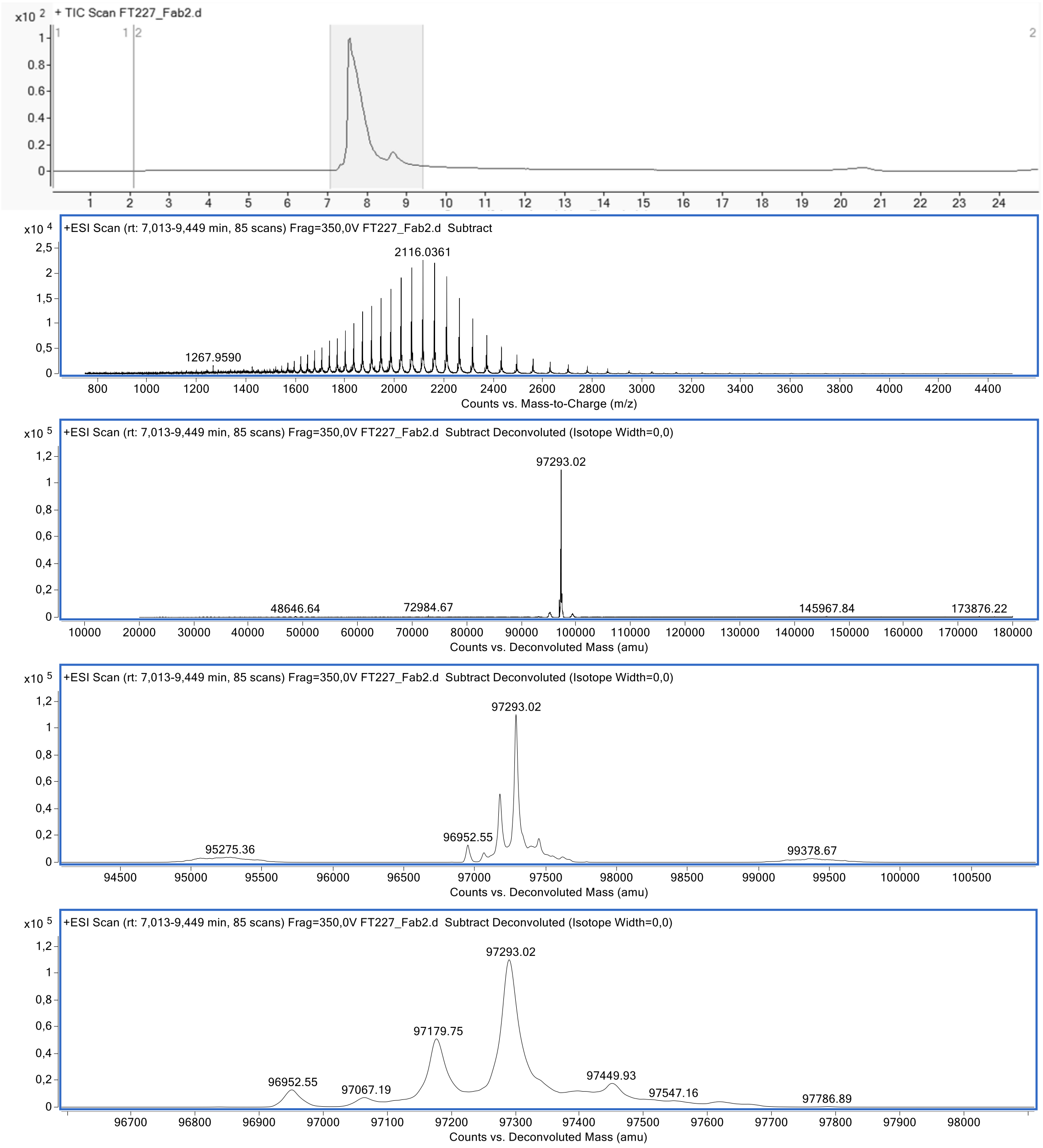

- <u>Fab' conjugate **24**</u>

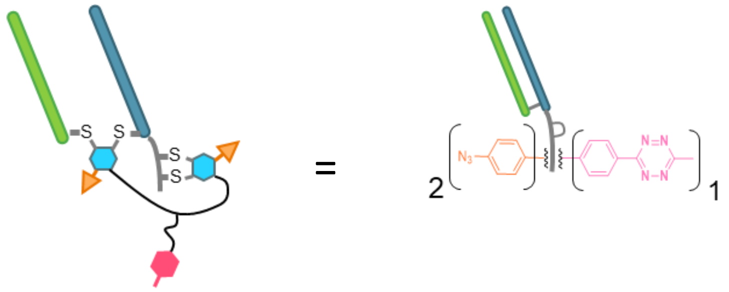

Expected mass: 50154.5 Da

Observed mass: **50156** Da

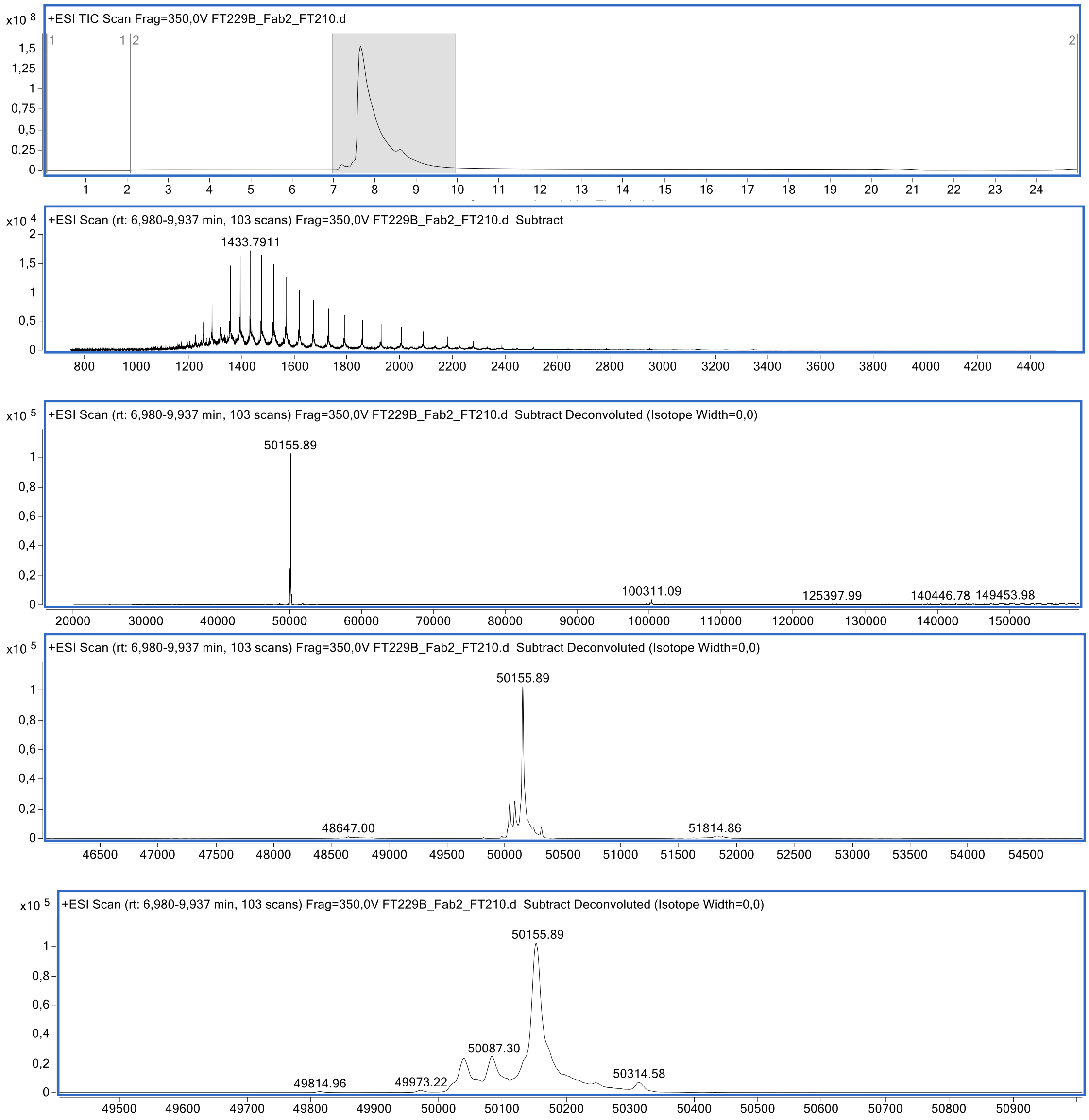

- <u>Fab' conjugate **25**</u>

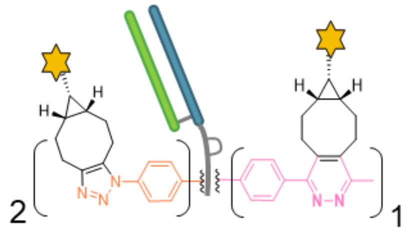

Expected mass: 52267 Da

Observed mass: **52269** Da, 34775 Da (PNGase F, which was added while not needed in this case since deglycosylation is only used when the protein contains an Fc fragment)

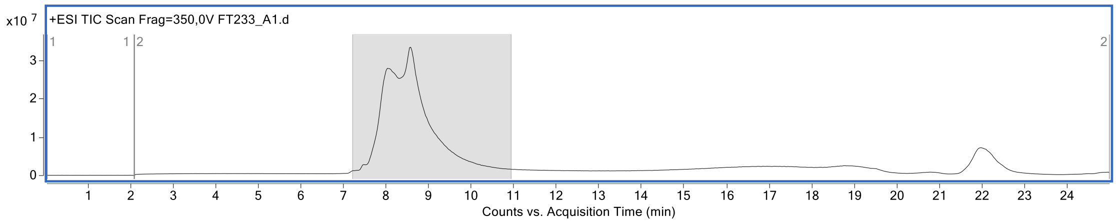

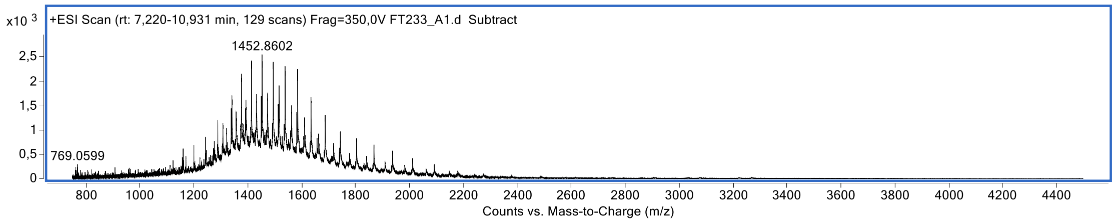

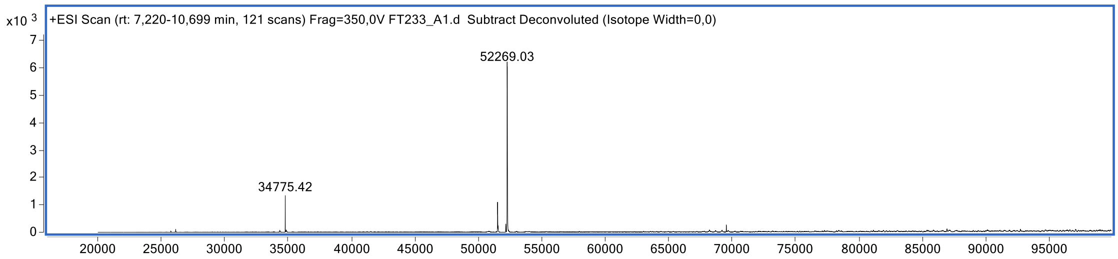

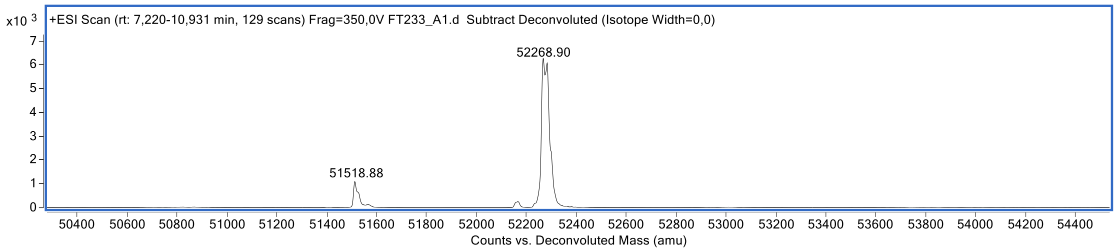

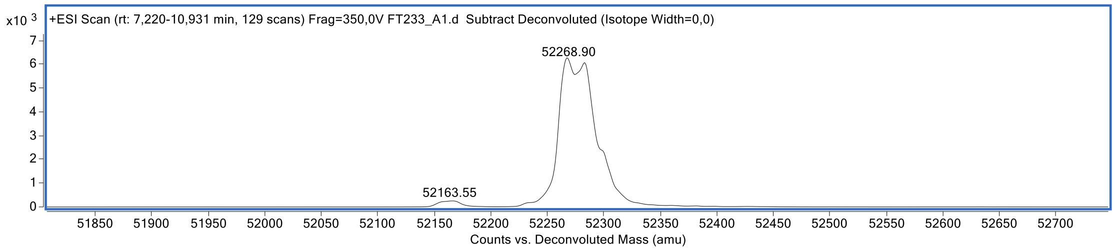

- <u>Fab’ conjugate **26**</u>

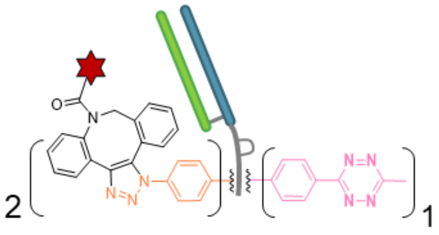

Expected mass: 52025 Da

Observed mass: **52027** Da, 34775 Da (PNGase F, which was added while not needed in this case since deglycosylation is only used when the protein contains an Fc fragment)

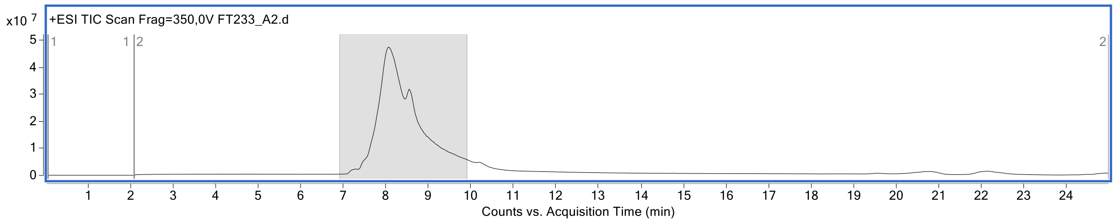

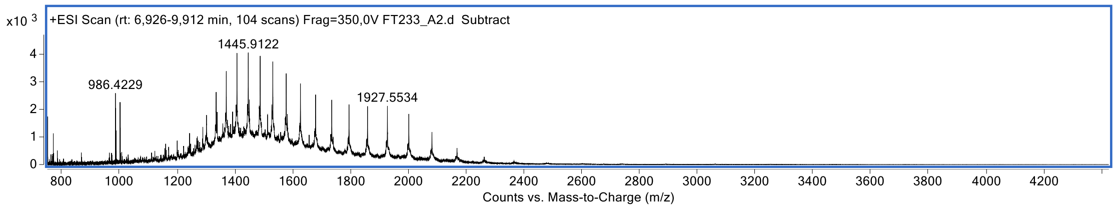

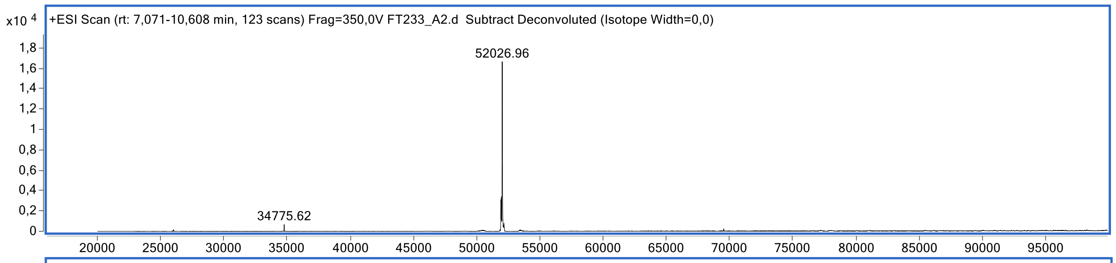

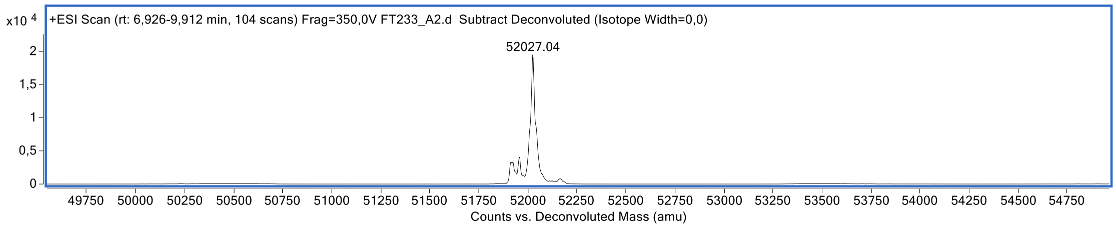

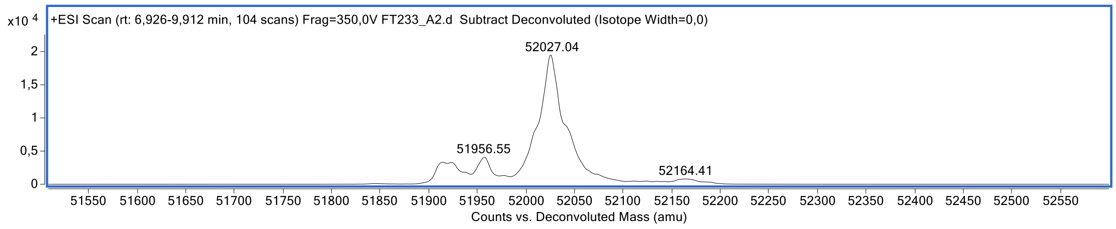

- <u>Fab’ conjugate **27**</u>

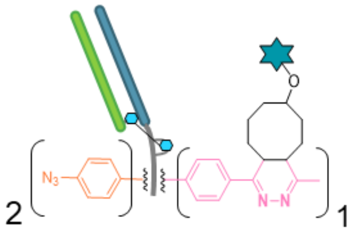

Expected mass: 50740 Da (Up to 2 loss of 131 Da *via* [CH_2-phenyl-N_3] elimination could also be observed)

Observed mass: **50740** Da, 50609 Da (loss of 131 Da through elimination of 1 x [CH_2-phenyl-N_3])

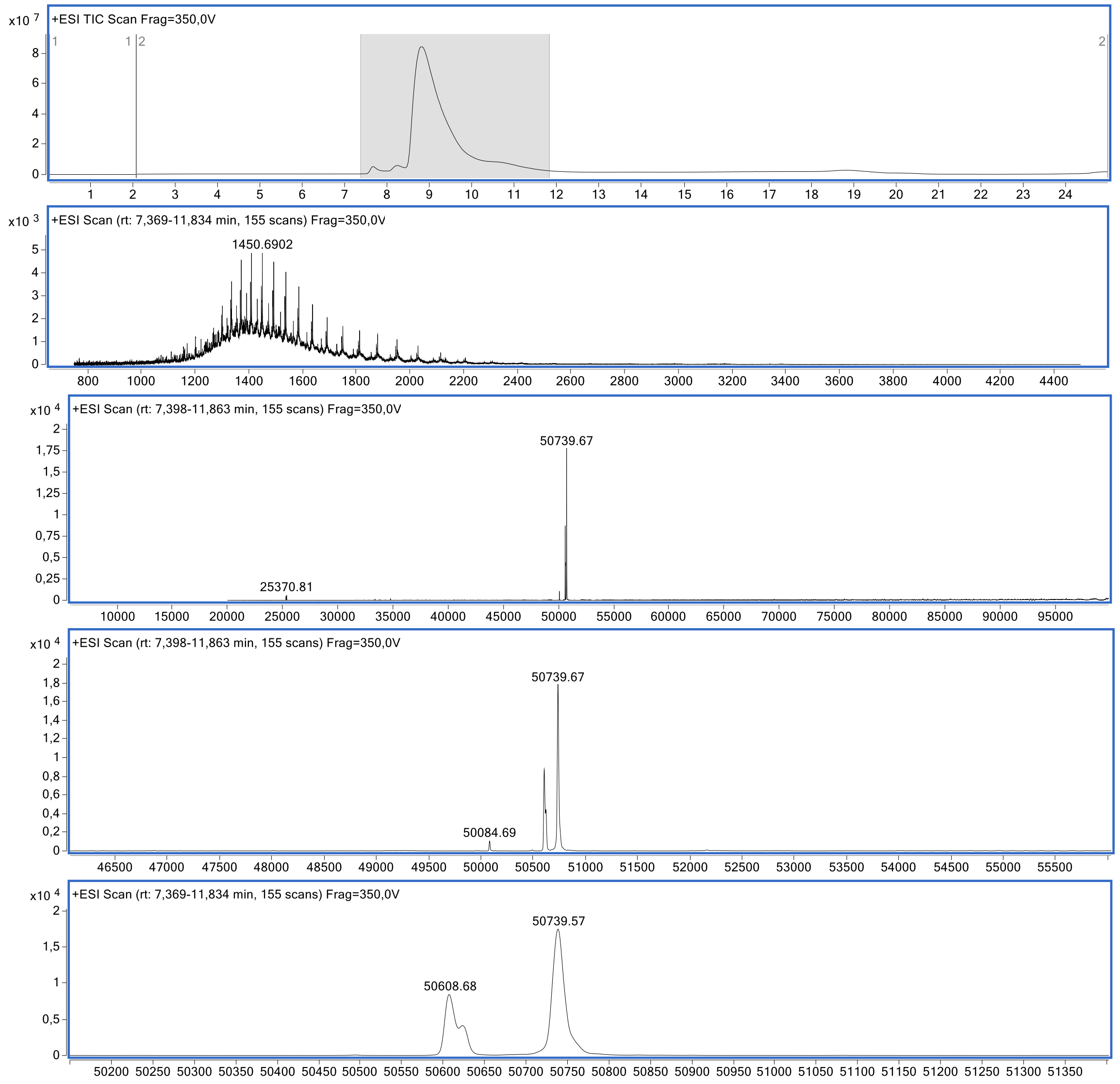

- <u>Fab’ conjugate **S27**</u>

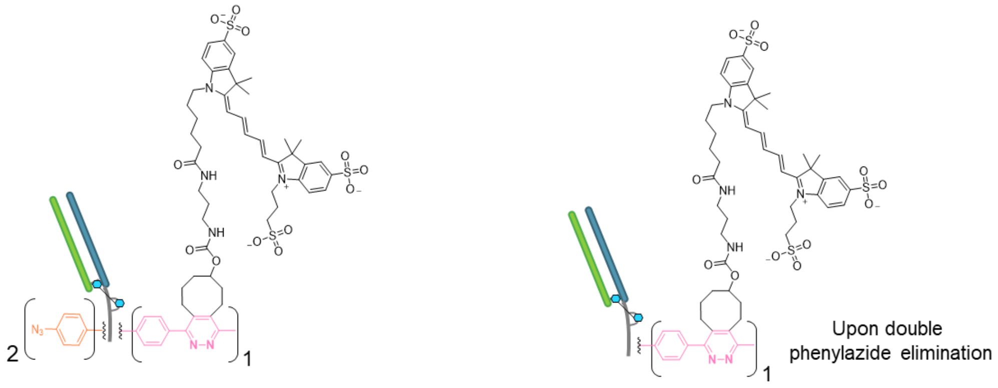

Expected mass: 51081, 50951 (elimination of 1 x [CH_2-phenyl-azide] motif), 50821 (elimination of both [CH_2-phenyl-azide] motifs).

Observed mass: **50823** Da (elimination of 2 x [CH_2-phenyl-N_3] moieties, major product); 50951 Da (elimination of 1 [CH_2-phenyl-N_3]), 51884 Da (elimination of 1 x [CH_2-phenyl-N_3], and addition of TCO-Cy5 on the other phenyl-N_3, not yet eliminated).

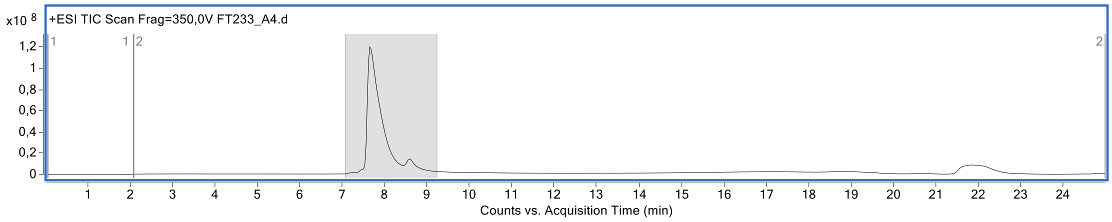

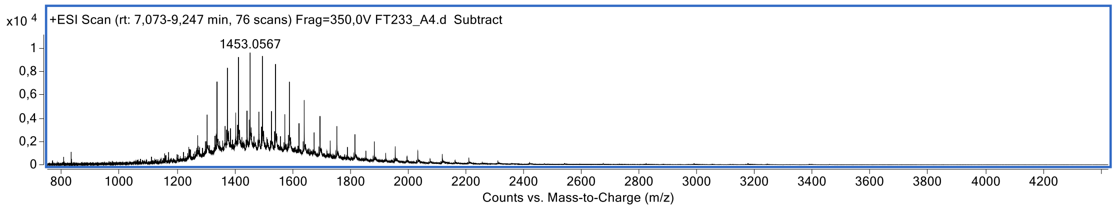

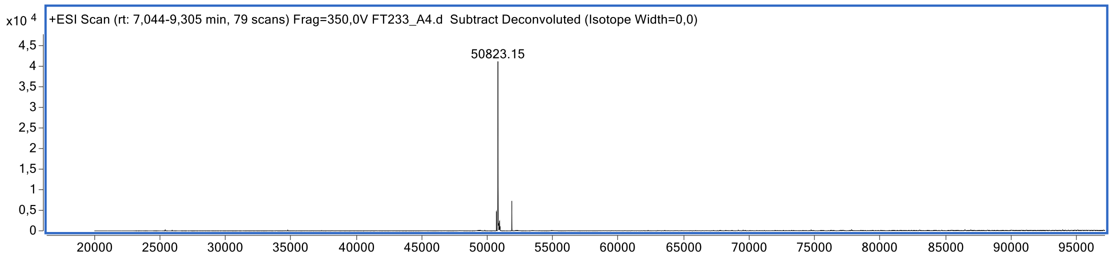

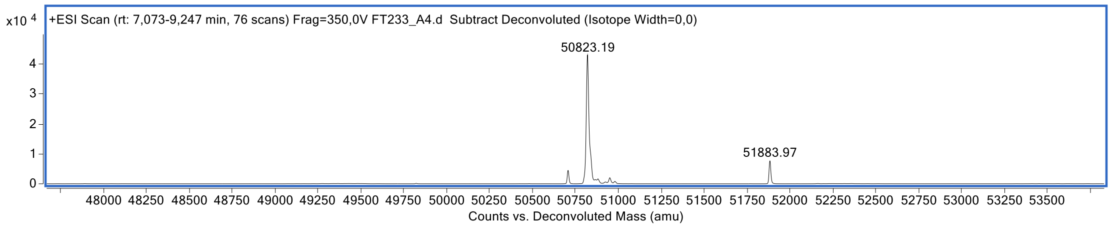

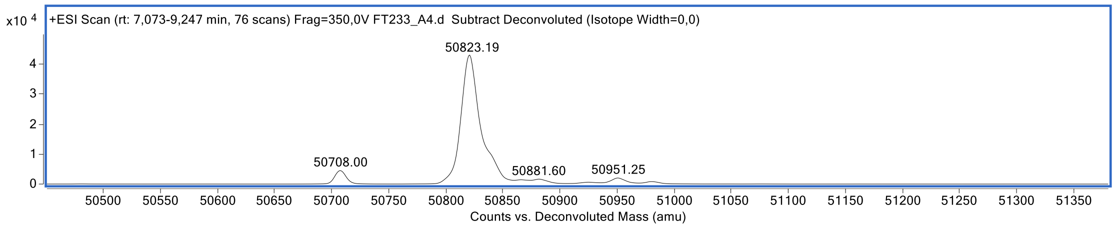

- <u>Fab’ conjugate **28**</u>

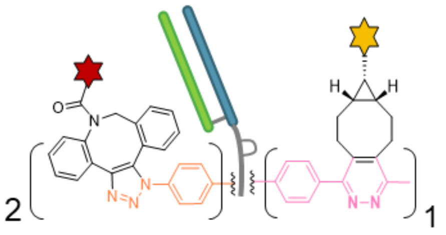

Expected mass: 52711 Da

Observed mass: **52716** Da

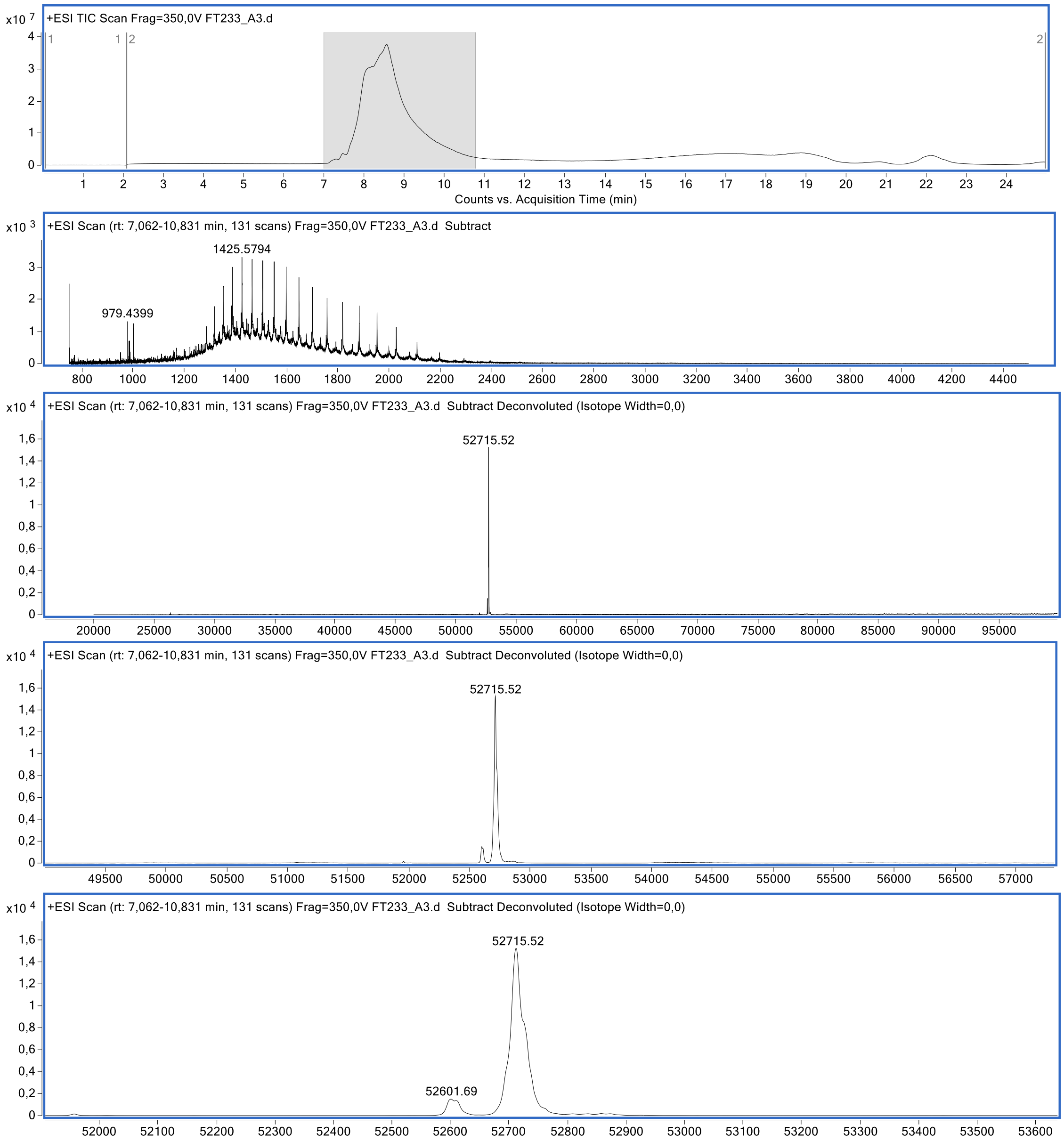

- <u>Fc fragment **29**</u>

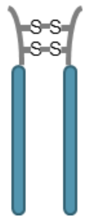

Expected mass: 49868 Da

Observed mass: 49868 Da; 34776 Da (PNGase F)

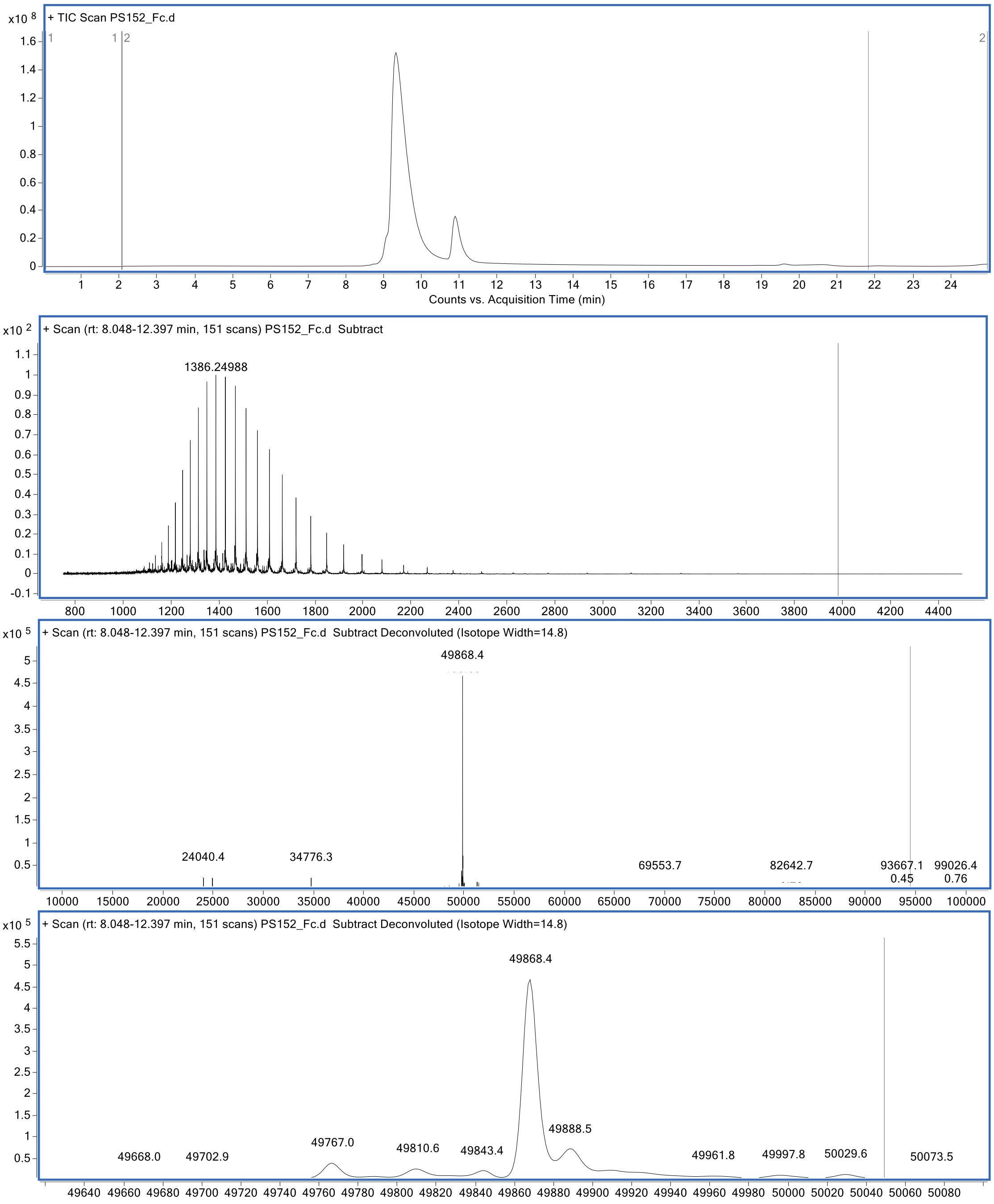

Fc conjugate **30**

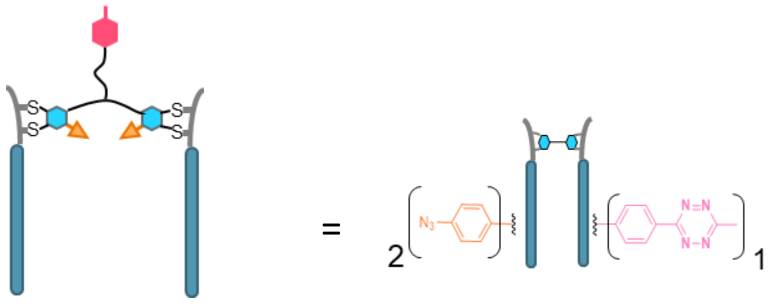

Expected mass: 51372 Da

Observed mass: **51379** Da

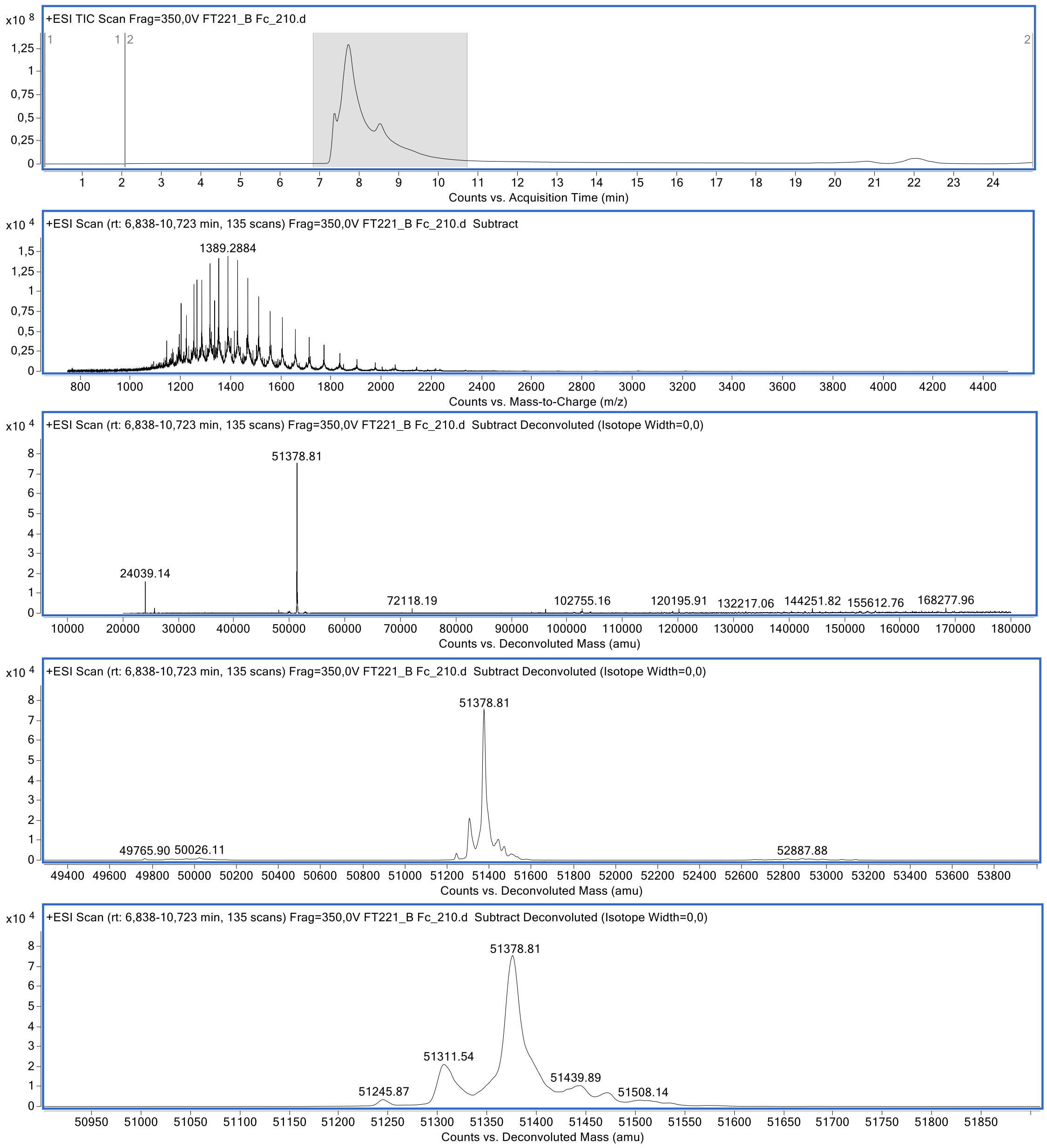

- Fc conjugate **31**

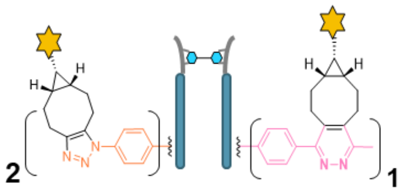

Expected mass: 53490 Da

Observed mass: **53491** Da

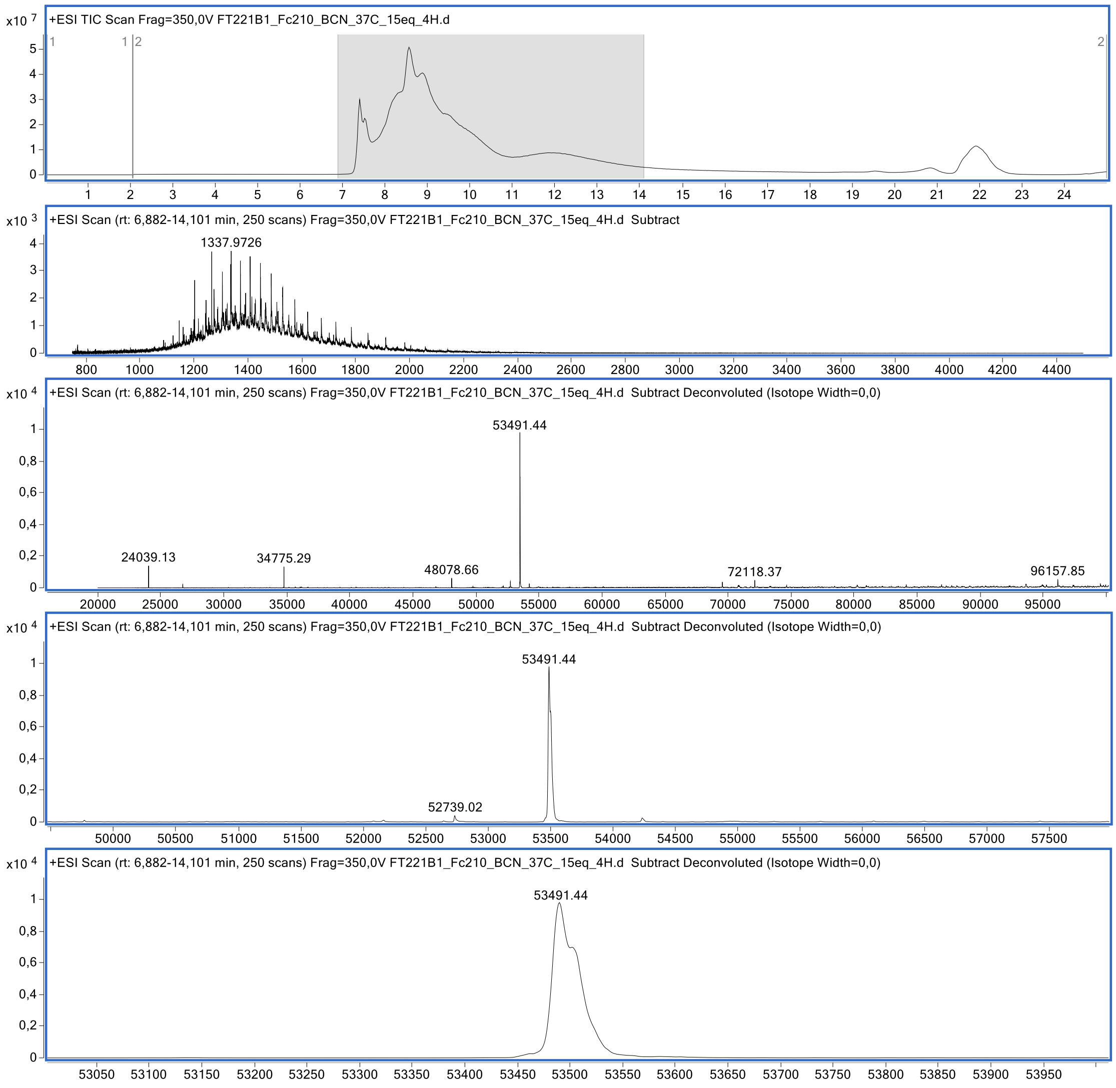

- Fc conjugate **32**

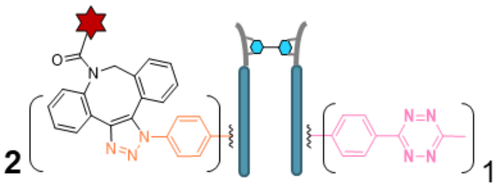

Expected mass: 53251 Da

Observed mass: **53249** Da, 34776 Da (PNGase F)

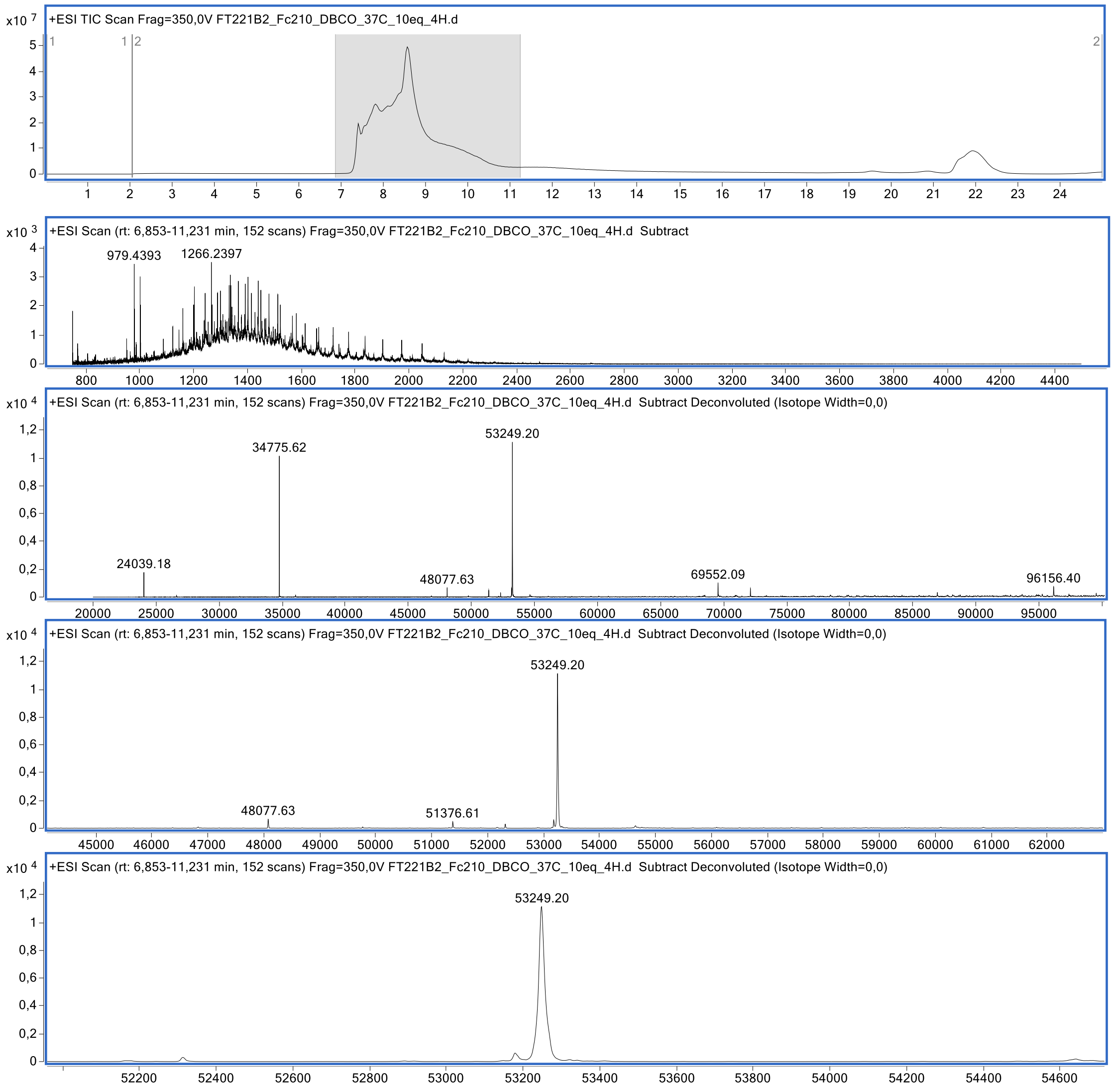

- Fc conjugate **33**

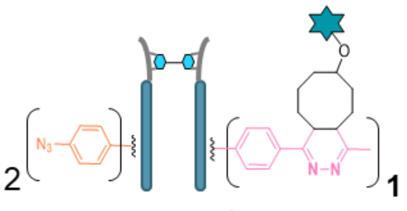

Expected mass: 51965 Da, (up to 2 loss of 131 Da *via* [CH_2-phenyl-N_3] degradation could be observed).

Observed mass: **51963** Da, 51830 Da (loss of 1 x [CH_2-phenyl-N_3]).

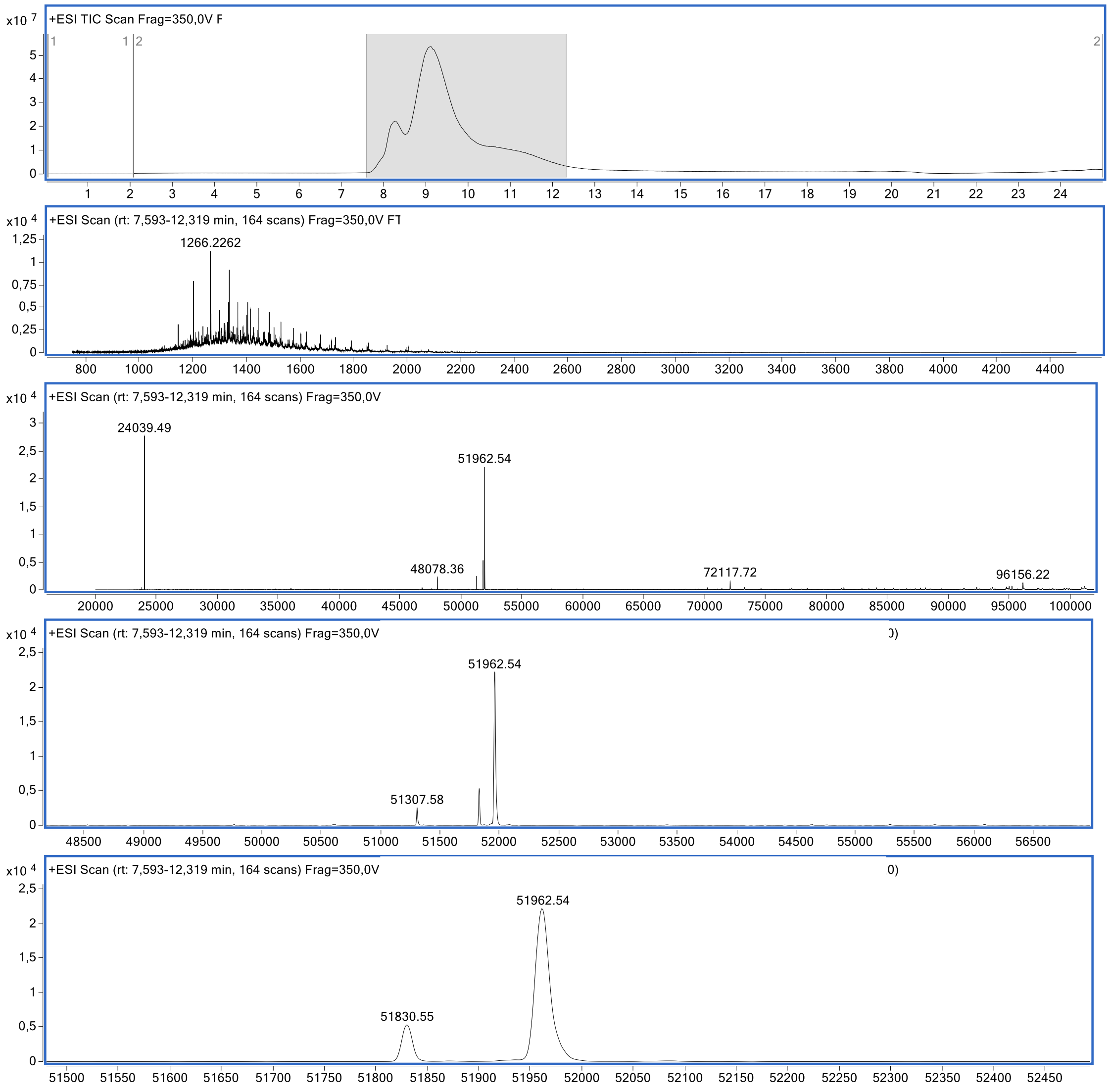

- Fc conjugate **S33**

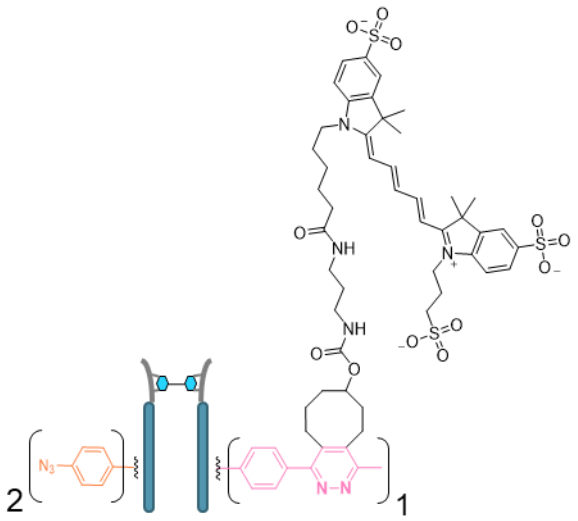

Expected mass: 52304 Da (up to 2 loss of 131 Da *via* [CH_2-phenyl-N_3] elimination could be observed).

Observed mass: 52311 Da**, 52177** Da (loss of 1 x [CH_2-phenyl-N_3])

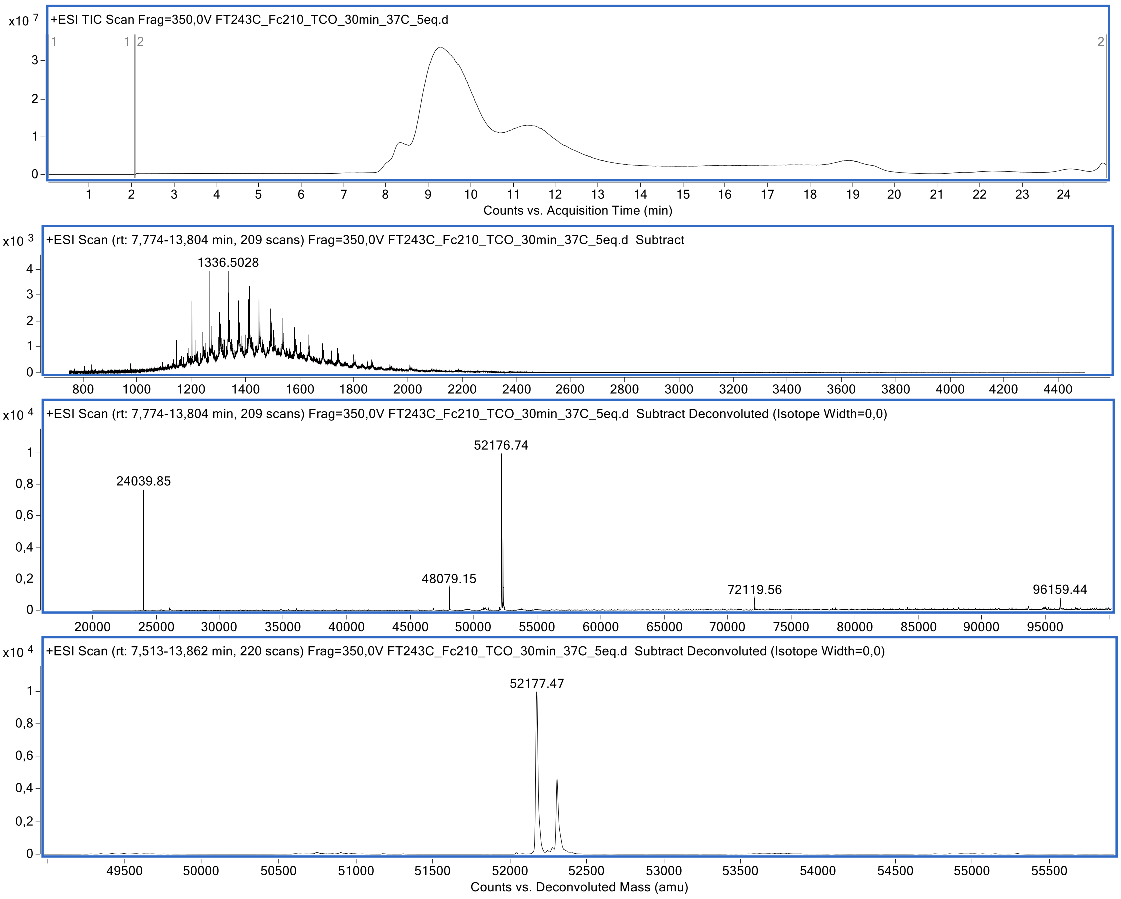

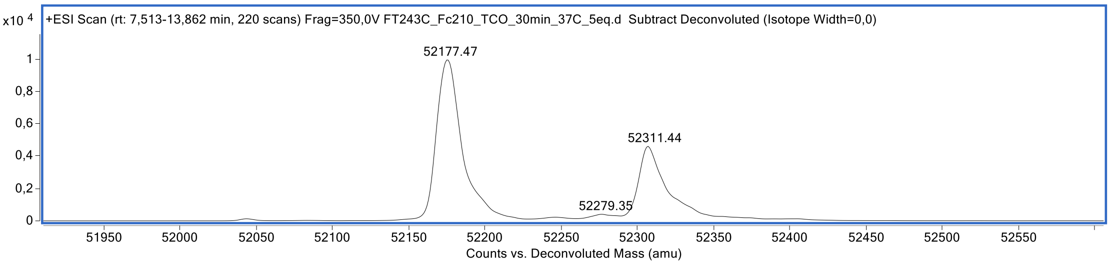

- <u>Fc conjugate **34**</u>

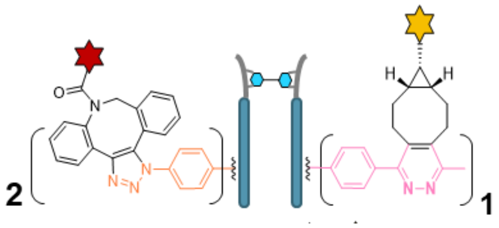

Expected mass: 53936 Da

Observed mass: **53936** Da

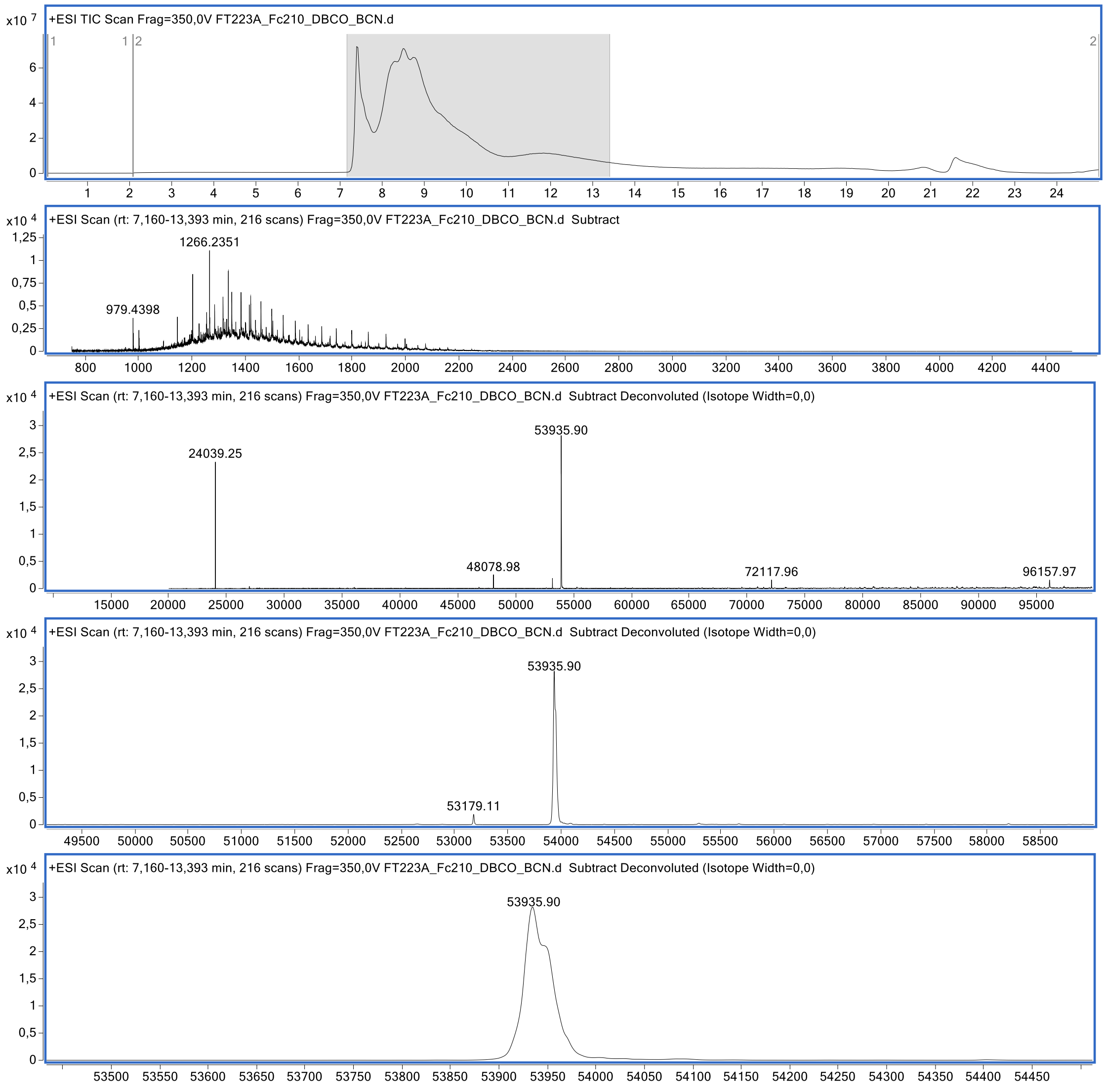

4. Study of the relationship between mAb/Fab molar ratio and mass peak intensity

In order to demonstrate that LC-MS analysis is not a quantative method, as opposed to SDS-PAGE gel (and gel densitometry analysis), a small study was carried out on three samples containing a mixture of Trastuzumab mAb (~150 KDa) and Trastuzumab Fab (~50 KDa) at different mAb/Fab molar ratios (50/50, 75/25 and 95/5), in water, at constant mAb concentration. The prepared mixtures were then deglycosylated and analysed by SDS-PAGE gel and LC-MS. The results demonstrate that the lower mass protein was detected with higher sensitivity than the larger one, as shown below:

Summarised LC-MS and SDS-PAGE data:

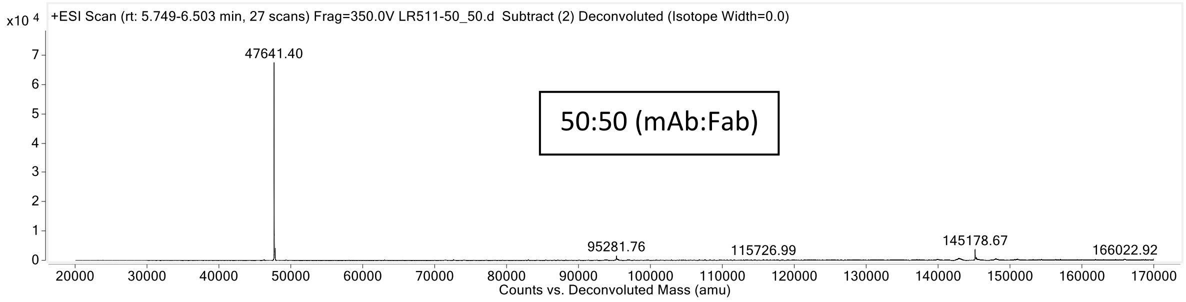

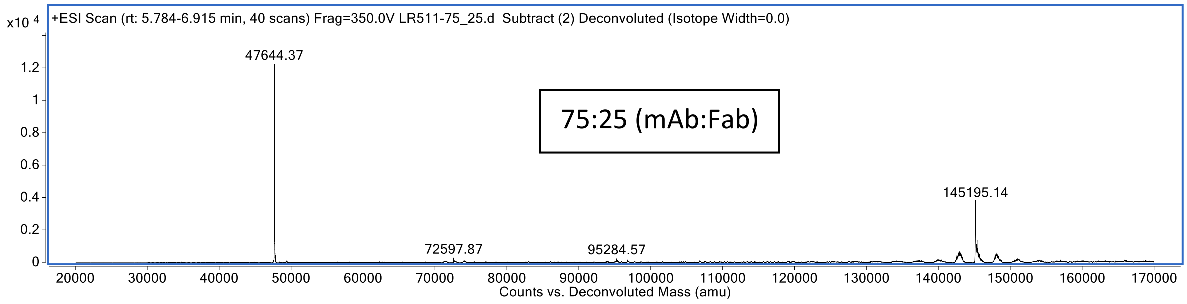

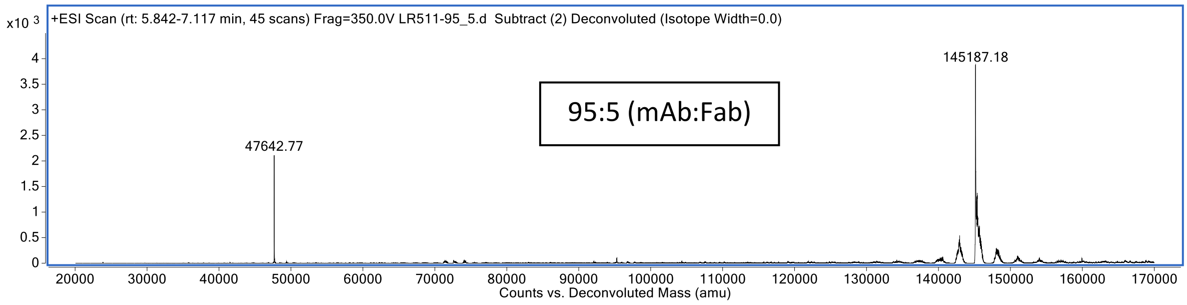

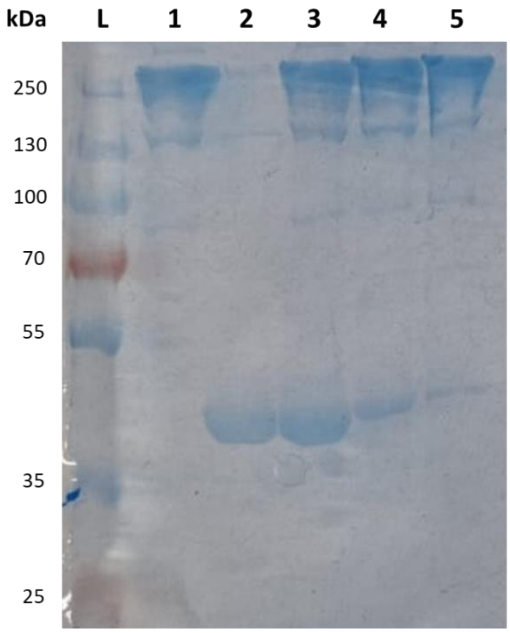

L: ladder;

Lane 1: Trastuzumab mAb (146 kDa);

Lane 2: Trastuzumab Fab (47 kDa);

Lane 3: mixture mAb:Fab (50:50);

Lane 4: mixture mAb:Fab (75:25);

Lane 5: mixture mAb:Fab (95:5)

mAb:Fab (50:50)

Expected masses: 47639 Da (Fab), 145178 Da (mAb)

Observed mass: 47641.4 Da (Fab), 145178.7 Da (mAb)

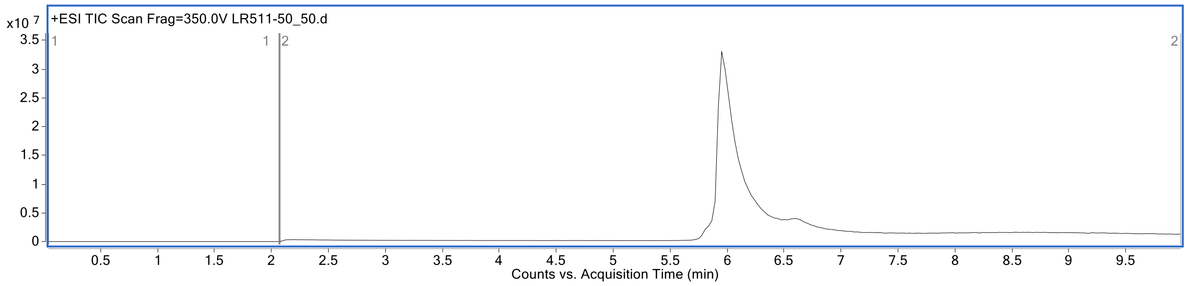

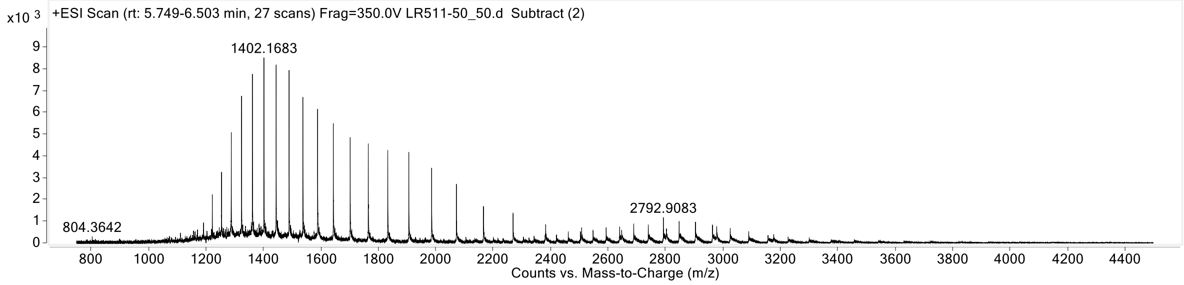

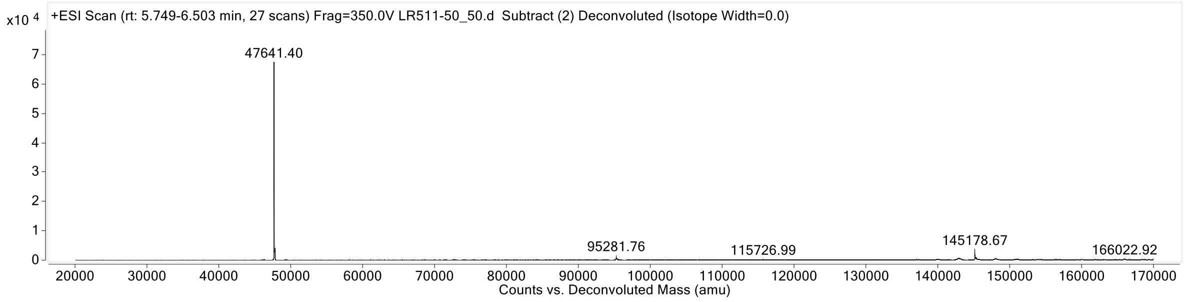

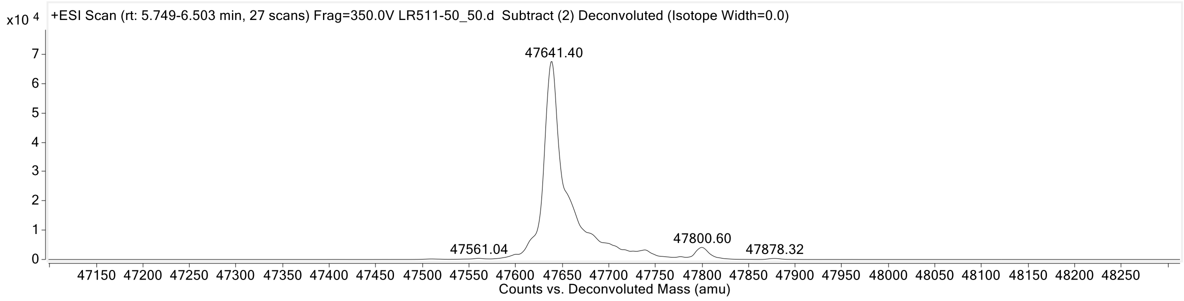

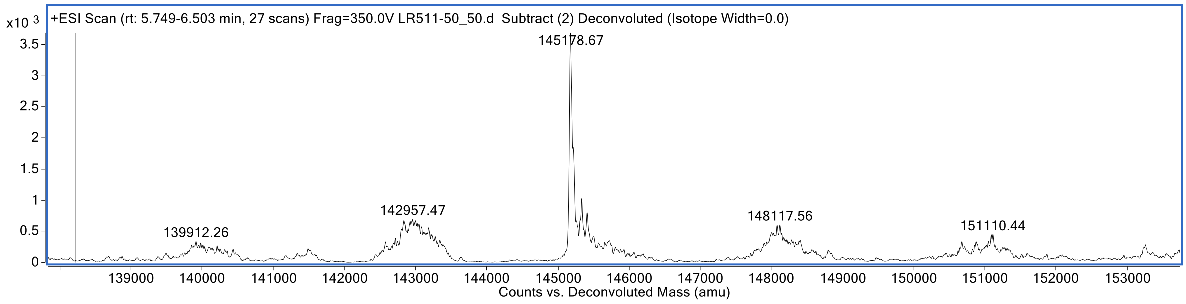

mAb:Fab (75:25)

Expected masses: 47639 Da (Fab), 145178 Da (mAb)
Observed mass: 47644.4 Da (Fab), 145185.1 Da (mAb)

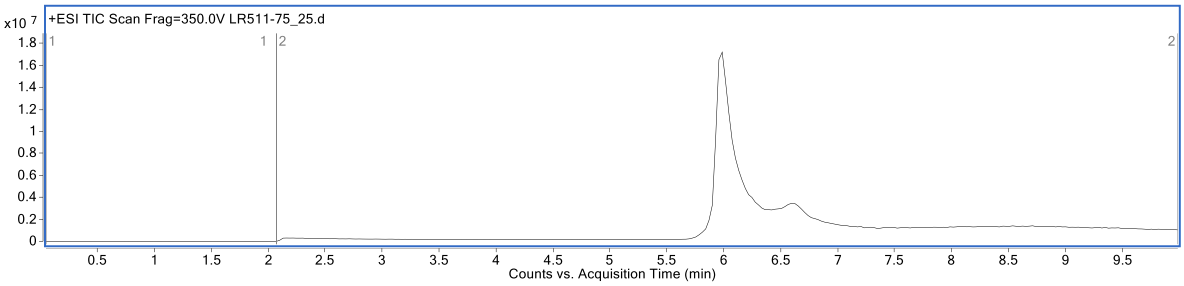

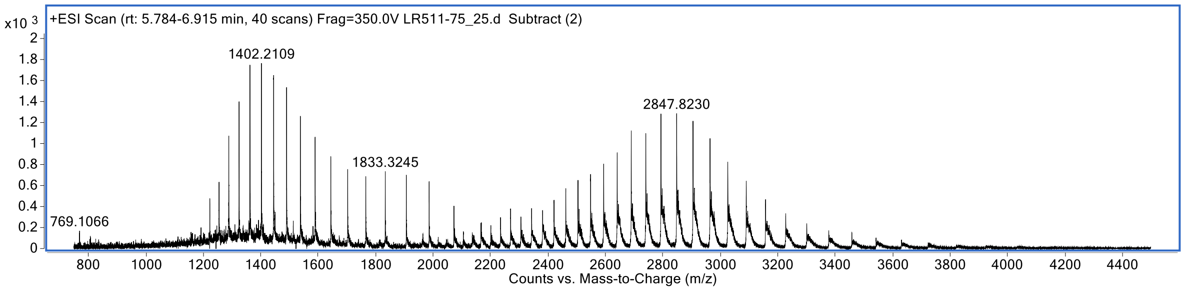

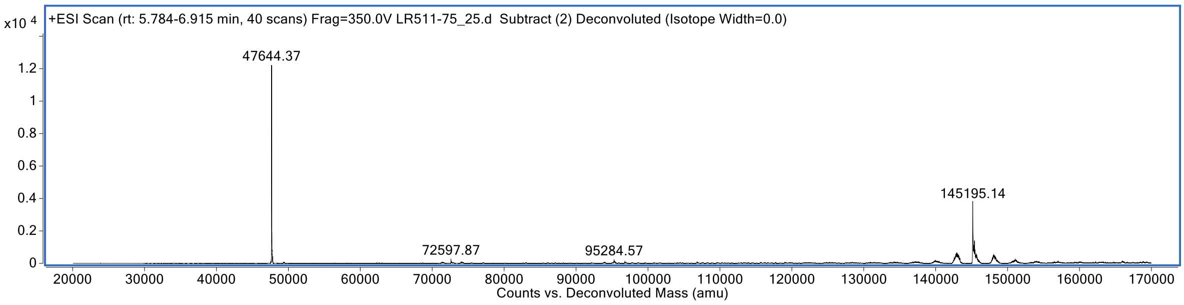

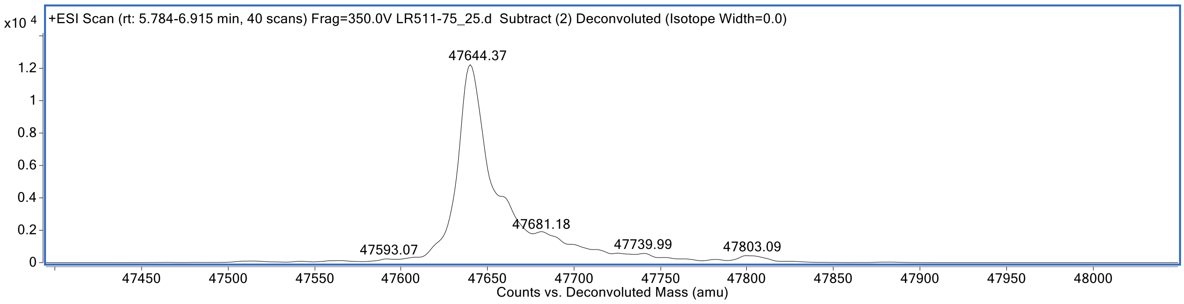

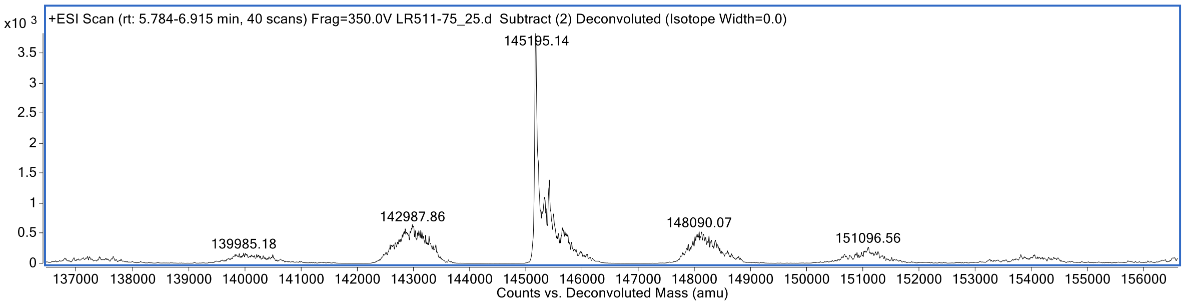

mAb:Fab (95:5)

Expected masses: 47639 Da (Fab), 145178 Da (mAb)

Observed mass: 47642.8 Da (Fab), 145187.2 Da (mAb)

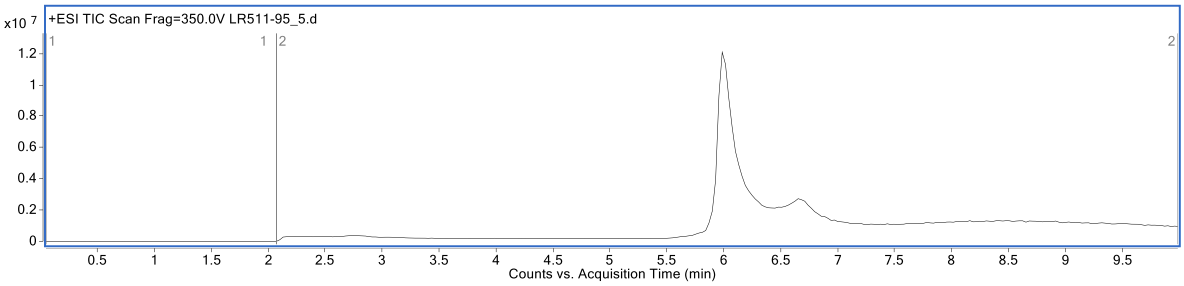

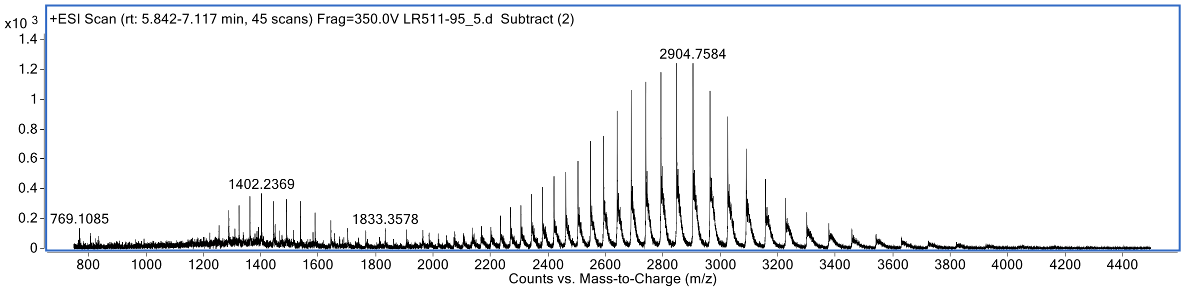

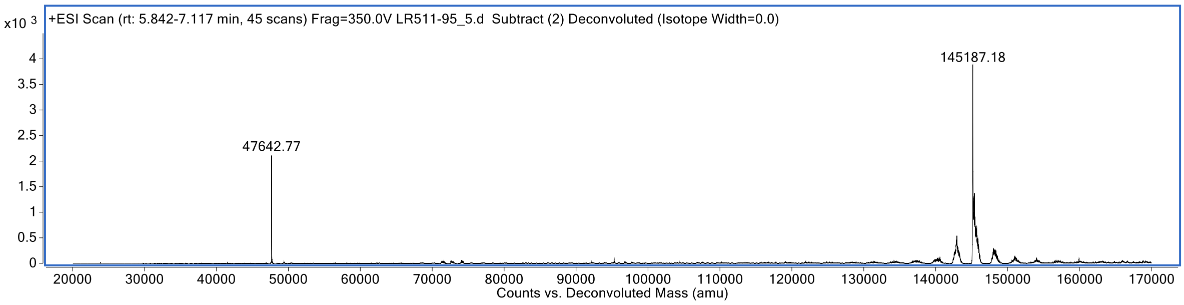

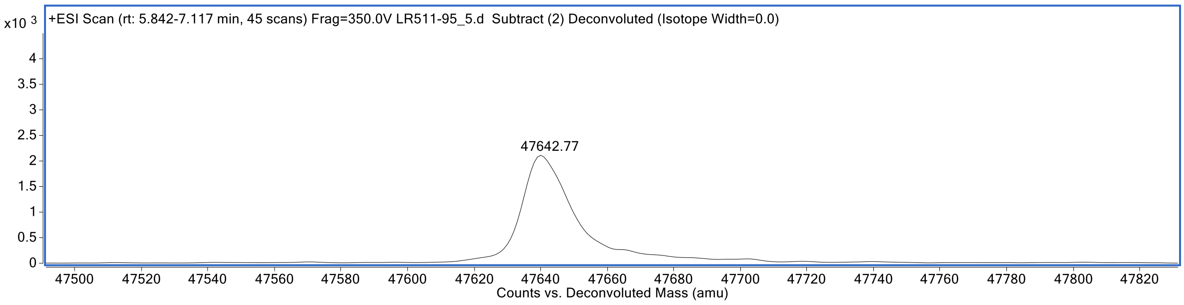

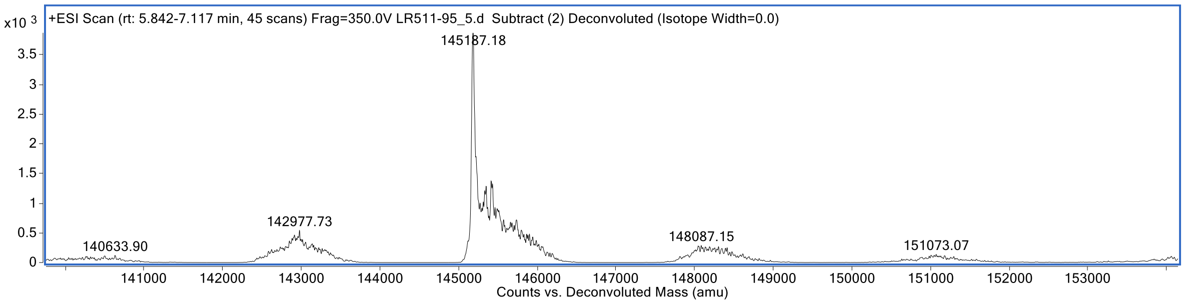

5. ELISA test – HER2 binding activity of conjugates **17** and **24**

ELISA assays were performed using 96-well Nuck™ plate. The plate was coated for 16 h at 4 °C with HER2 (Sino Biological, 100 µL/well, 0.25 µg/mL in PBS pH = 7.4), all wells except row D and H. After washing (3 x 0.1% Tween® 20 in PBS, followed by 3 x PBS), wells were blocked for 1 h at room temperature with 5% Marvel milk powder (Premier foods) in PBS (200 µL/well). The wells were then washed (3 x 0.1% Tween® 20 in PBS, followed by 3 x PBS), and the following dilutions of appropriate trastuzumab Fab'/mAb conjugates were applied: 810 nM, 270 nM, 90 nM, 30 nM, 10 nM, 3.33 nM, 1.11 nM, 0.37 nM, 0.123 nM, 0.0412 nM, 0.0137 nM, prepared in 1% Marvel solutions in PBS (100 µL/well), added to rows A-C (columns 1-11) and E-G (columns 1-11). The plate was then incubated at room temperature for 1 h. After 1 h, the plate was washed (3 x 0.1% Tween® 20 in PBS, followed by 3 x PBS), and the detection antibody (anti-Human IgG, Fab specific horseradish peroxidase (HRP) antibody, Sigma Aldrich, 1:5000 in 1% Marvel solution in 0.1% Tween® 20 in PBS) was added to the whole plate (100 µL/well), and incubated at room temperature for 1 h. After that, the plate was washed (3 x 0.1% Tween® 20 in PBS, followed by 3 x PBS), and *o*-phenylenediamine hydrochloride (Sigma-Aldrich, 100 µL/well, 0.5 mg/mL in a phosphate-citrate buffer with sodium perborate) was added to the whole plate, left for 15-30 min in the dark (monitor the colour development) at room temperature. Once a yellow-orange colour was observed, the reaction was stopped by the addition of HCl (4 M, 50 µL/well) to the whole plate. Absorbance was immediately measured at 450 nm and was corrected by subtracting the average of the negative control absorbances. Each sample was tested in triplicate and errors are shown in the standard deviation of the average. ELISA data was analysed with Graphpad Prism 8.4.2 (using equation log(agonist) vs. response (three parameters)).

HER2 binding activity of PD mAb conjugate **17**:

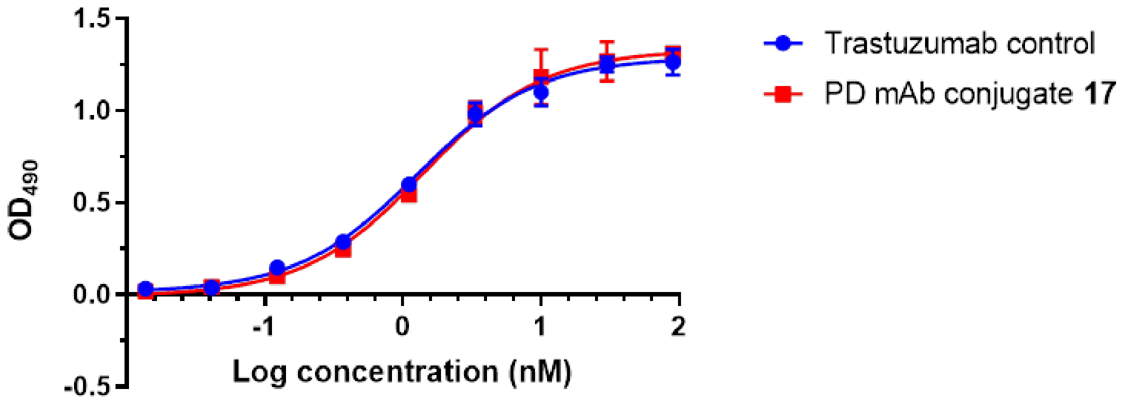

HER2 binding activity of PD Fab' conjugate **24**:

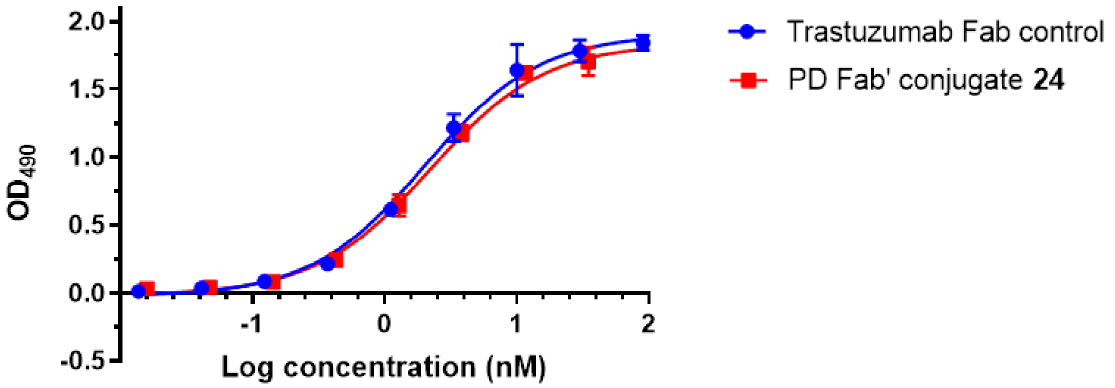

References

1 A. Maruani, S. Alom, P. Canavelli, M. T. W. Lee, R. E. Morgan, V. Chudasama and S. Caddick, *Chem. Commun.*, 2015, **51**, 5279–5282.

2 H. L. Evans, Q.-D. Nguyen, L. S. Carroll, M. Kaliszczak, F. J. Twyman, A. C. Spivey and E. O. Aboagye, *Chem. Commun.*, 2014, **50**, 9557–9560.

3 A. J. C. Sarris, T. Hansen, M. A. R. de Geus, E. Maurits, W. Doelman, H. S. Overkleeft, J. D. C. Codée, D. V. Filippov and S. I. van Kasteren, *Chem. Eur. J.*, 2018, **24**, 18075–18081.

4 M. T. W. Lee, A. Maruani, D. A. Richards, J. R. Baker, S. Caddick and V. Chudasama, *Chem. Sci.*, 2017, **8**, 2056–2060.

5 J. K. W. Chui and T. M. Fyles, *Org. Biomol. Chem.*, 2014, **12**, 3622–3634.

6 S. S. Matikonda, D. L. Orsi, V. Staudacher, I. A. Jenkins, F. Fiedler, J. Chen and A. B. Gamble, *Chem. Sci.*, 2015, **6**, 1212–1218.

7 R. J. Brezski and R. E. Jordan, *mAbs*, 2010, **2**, 212–220.
